# Supplementary material for: Frailty-adjusted therapy in Transplant Non-Eligible patients with newly diagnosed Multiple Myeloma (FiTNEss (UK-MRA Myeloma XIV Trial)): a study protocol for a randomised phase III trial
Source: BMJ Open. 2022 Jun 2;12(6):e056147. doi: 10.1136/bmjopen-2021-056147 (PMC9163533; doi:10.1136/bmjopen-2021-056147)
Supplement: Supplementary data [file bmjopen-2021-056147supp001.pdf]

Myeloma XIV Supplementary Material to Protocol Paper
EudraCT number: **2018-003590-10**

Supplementary Material:

Contents

Glossary of Terms 2
List of Sites in the Fitness Trial 4
Spirit Checklist : Reporting checklist for protocol of a clinical trial. 5
Renal and Liver Function 11
Concomitant Medication 12
Exploratory analysis 13
Subgroup analysis 13
Informed Consent Material 15
Bone marrow and Blood sample consent form 15
Main Trial consent 18
Central Sample Analysis 21
References 23

Coulson AB, *et al. BMJ Open* 2022; 12:e056147. doi: 10.1136/bmjopen-2021-056147

Myeloma XIV Supplementary Material to Protocol Paper
EudraCT number: **2018-003590-10**

Glossary of Terms

AE	Adverse event
ADL	Katz Activity of Daily Living
ALT	Alanine aminotransferase
AR	Adverse reaction
ANC	Absolute Neutrophil Count
AST	Aspartate aminotransferase
β2M	Beta2-microglobulin
CCI	Charlson Comorbidity Index
CI	Chief Investigator
CR	Complete response
CRF	Case report form
CTCAE	Common Terminology Criteria for Adverse Events
CTRU	Clinical Trials Research Unit
DMEC	Data Monitoring and Ethics Committee
eCRF	Electronic Case Report Form
GI	Gastrointestinal
HEAP	Health Economics Analysis Plan
HMDS	Haematological Malignancy Diagnostic Service
IADL	Lawton Instrumental Activity of Daily Living
IMiD	Immunomodulatory Drug
IMP	Investigational medicinal product
IMWG	International Myeloma Working Group
ITT	Intention to treat
mg	Milligram
MM	Multiple myeloma
MRD	Minimal Residual Disease
MR	Minimal response
NCI	National Cancer Institute
ORR	Overall response rate
OS	Overall survival
PD	Progressive disease
PFS	Progression-free survival
PFS2	Time from randomisation to second progression
PK	Pharmacokinetic(s)
PI	Proteasome Inhibitor
PR	Partial response
PS	Performance status
QoL	Quality of life
R1	Randomisation 1
R2	Randomisation 2
REC	Research Ethics Committee
SD	Stable disease
SAE	Serious adverse event
SAR	Serious adverse reaction
SPM	Secondary Primary Malignancy
SmPC	Summary of product characteristics

Coulson AB, *et al. BMJ Open* 2022; 12:e056147. doi: 10.1136/bmjopen-2021-056147

SUSAR	Suspected unexpected serious adverse reaction
TMG	Trial Management Group
TNE	Transplant non-eligible
TSC	Trial Steering Committee
UK-MRA	United Kingdom Myeloma Research Alliance
ULN	Upper Limit of normal
VGPR	Very good partial response
VTE	Venous thromboembolism

Myeloma XIV Supplementary Material to Protocol Paper
EudraCT number: **2018-003590-10**

List of Sites in the Fitness Trial

Sites		
Aberdeen Royal Infirmary	Kettering general	Salford Royal Hospital
Blackpool Victoria	Kidderminster Hospital	Salisbury District Hospital
Bristol Haematology & Oncology Centre	Kings College Hospital, Denmark Hill	South Tyneside Hospital
Calderdale Royal Hospital	Leicester Royal Infirmary	Southampton Hospital
Castle Hill Hospital	Lincoln County Hospital	Southmead Hospital
Cheltenham Hospital	Maidstone Hospital	St Bartholomew Hospital
Clatterbridge Cancer Centre	Manchester Royal Infirmary	St George's Hospital, Tooting
Colchester District	New Cross Hospital	St Helens Hospital
Countess of Chester	Ninewells	St Helier Hospital
County Hospital	Norfolk & Norwich University Hospital	St Richard's Hospital
Croydon University Hospital	Nottingham City Hospital	St. James's University Hospital
Derriford Hospital	Peterborough City Hospital	St. John's. Hospital, Howden
Dewsbury and District Hospital	Pilgrim Hospital	Sunderland Royal Hospital
Dewsbury and District Hospital	Pontefract Hospital	The County Hospital, Wye Valley
Epsom General Hospital	Princess Royal	Torbay Hospital
Freeman Hospital	Queen Alexandra Hospital	TRAFFORD GENERAL HOSPITAL
Friarage Hospital	Queen Elizabeth Hospital the Queen Mother	University College London Hospital
Glan Clywd	Queen Elizabeth Hospital, Birmingham	Western General
Gloucestershire Royal	Queen Elizabeth Hospital, King's Lynn	Whiston Hospital
Goodhope	Queens Hospital, Romford	William Harvey
Grantham & District	Royal Albert Edward Infirmary	Worcestershire Royal Hospital
Guys Hospital	Royal Berkshire	Worcestershire Royal Hospital
Harrogate District Hospital	Royal Bournemouth General	Worthing Hospital
Heartlands	Royal Derby	Wrexham Maelor Hospital
Hillingdon Hospital	Royal Devon & Exeter Hospital	Wythenshaw Hospital
Hinchingbrooke Hospital	Royal Oldham	YSBYTY GWYNEDD
Huddersfield Royal Infirmary	Royal Stoke University Hospital	North Middlesex University Hospital
James Cook University Hospital	Royal United Hospital, Bath	Royal Hallamshire
Kent & Canterbury Hospital	Russells Hall Hospital	

*In total the trial will be conducted in 87 centres around the UK

Coulson AB, *et al. BMJ Open* 2022; 12:e056147. doi: 10.1136/bmjopen-2021-056147

Myeloma XIV Supplementary Material to Protocol Paper
EudraCT number: **2018-003590-10**

Spirit Checklist : Reporting checklist for protocol of a clinical trial.

Based on the SPIRIT guidelines.

Chan A-W, Tetzlaff JM, Gøtzsche PC, Altman DG, Mann H, Berlin J, Dickersin K, Hróbjartsson A, Schulz KF, Parulekar WR, Krleža-Jerić K, Laupacis A, Moher D. SPIRIT 2013 Explanation and Elaboration: Guidance for protocols of clinical trials. BMJ. 2013;346:e7586

		Reporting Item	Page Number
**Administrative information**			
Title	#1	Descriptive title identifying the study design, population, interventions, and, if applicable, trial acronym	1
Trial registration	#2a	Trial identifier and registry name. If not yet registered, name of intended registry	1,2
Trial registration: data set	#2b	All items from the World Health Organization Trial Registration Data Set	NA
Protocol version	#3	Date and version identifier	18
Funding	#4	Sources and types of financial, material, and other support	20
Roles and responsibilities: contributorship	#5a	Names, affiliations, and roles of protocol contributors	1, 20
Roles and responsibilities: sponsor contact information	#5b	Name and contact information for the trial sponsor	20
Roles and responsibilities: sponsor and funder	#5c	Role of study sponsor and funders, if any, in study design; collection, management, analysis, and interpretation of data; writing of the report; and the decision to submit the report for publication, including whether they will have ultimate authority over any of these activities	20,21
Roles and responsibilities: committees	#5d	Composition, roles, and responsibilities of the coordinating centre, steering committee, endpoint adjudication committee, data management team, and other individuals or groups overseeing the trial, if applicable (see Item 21a for data monitoring committee)	16
**Introduction**			

Myeloma XIV Supplementary Material to Protocol Paper
EudraCT number: **2018-003590-10**

Background and rationale	#6a	Description of research question and justification for undertaking the trial, including summary of relevant studies (published and unpublished) examining benefits and harms for each intervention	4,5
Background and rationale: choice of comparators	#6b	Explanation for choice of comparators	4,5,6
Objectives	#7	Specific objectives or hypotheses	6
Trial design	#8	Description of trial design including type of trial (eg, parallel group, crossover, factorial, single group), allocation ratio, and framework (eg, superiority, equivalence, non-inferiority, exploratory)	6
**Methods: Participants, interventions, and outcomes**			
Study setting	#9	Description of study settings (eg, community clinic, academic hospital) and list of countries where data will be collected. Reference to where list of study sites can be obtained	6
Eligibility criteria	#10	Inclusion and exclusion criteria for participants. If applicable, eligibility criteria for study centres and individuals who will perform the interventions (eg, surgeons, psychotherapists)	6
Interventions: description	#11a	Interventions for each group with sufficient detail to allow replication, including how and when they will be administered	9
Interventions: modifications	#11b	Criteria for discontinuing or modifying allocated interventions for a given trial participant (eg, drug dose change in response to harms, participant request, or improving / worsening disease)	9,10
Interventions: adherance	#11c	Strategies to improve adherence to intervention protocols, and any procedures for monitoring adherence (eg, drug tablet return; laboratory tests)	10
Interventions: concomitant care	#11d	Relevant concomitant care and interventions that are permitted or prohibited during the trial	10

Outcomes	#12	Primary, secondary, and other outcomes, including the specific measurement variable (eg, systolic blood pressure), analysis metric (eg, change from baseline, final value, time to event), method of aggregation (eg, median, proportion), and time point for each outcome. Explanation of the clinical relevance of chosen efficacy and harm outcomes is strongly recommended	11
Participant timeline	#13	Time schedule of enrolment, interventions (including any run-ins and washouts), assessments, and visits for participants. A schematic diagram is highly recommended (see Figure)	11,12
Sample size	#14	Estimated number of participants needed to achieve study objectives and how it was determined, including clinical and statistical assumptions supporting any sample size calculations	12,13
Recruitment	#15	Strategies for achieving adequate participant enrolment to reach target sample size	13
**Methods: Assignment of interventions (for controlled trials)**			
Allocation: sequence generation	#16a	Method of generating the allocation sequence (eg, computer-generated random numbers), and list of any factors for stratification. To reduce predictability of a random sequence, details of any planned restriction (eg, blocking) should be provided in a separate document that is unavailable to those who enrol participants or assign interventions	13,14
Allocation concealment mechanism	#16b	Mechanism of implementing the allocation sequence (eg, central telephone; sequentially numbered, opaque, sealed envelopes), describing any steps to conceal the sequence until interventions are assigned	13
Allocation: implementation	#16c	Who will generate the allocation sequence, who will enrol participants, and who will assign participants to interventions	14
Blinding (masking)	#17a	Who will be blinded after assignment to interventions (eg, trial participants, care	14

		providers, outcome assessors, data analysts), and how	
Blinding (masking): emergency unblinding	#17b	If blinded, circumstances under which unblinding is permissible, and procedure for revealing a participant's allocated intervention during the trial	14
**Methods: Data collection, management, and analysis**			
Data collection plan	#18a	Plans for assessment and collection of outcome, baseline, and other trial data, including any related processes to promote data quality (eg, duplicate measurements, training of assessors) and a description of study instruments (eg, questionnaires, laboratory tests) along with their reliability and validity, if known. Reference to where data collection forms can be found, if not in the protocol	14
Data collection plan: retention	#18b	Plans to promote participant retention and complete follow-up, including list of any outcome data to be collected for participants who discontinue or deviate from intervention protocols	14
Data management	#19	Plans for data entry, coding, security, and storage, including any related processes to promote data quality (eg, double data entry; range checks for data values). Reference to where details of data management procedures can be found, if not in the protocol	14,15
Statistics: outcomes	#20a	Statistical methods for analysing primary and secondary outcomes. Reference to where other details of the statistical analysis plan can be found, if not in the protocol	15,16
Statistics: additional analyses	#20b	Methods for any additional analyses (eg, subgroup and adjusted analyses)	16
Statistics: analysis population and missing data	#20c	Definition of analysis population relating to protocol non-adherence (eg, as randomised analysis), and any statistical methods to handle missing data (eg, multiple imputation)	16,17
**Methods: Monitoring**			

Myeloma XIV Supplementary Material to Protocol Paper
EudraCT number: **2018-003590-10**

Data monitoring: formal committee	#21a	Composition of data monitoring committee (DMC); summary of its role and reporting structure; statement of whether it is independent from the sponsor and competing interests; and reference to where further details about its charter can be found, if not in the protocol. Alternatively, an explanation of why a DMC is not needed	16,17
Data monitoring: interim analysis	#21b	Description of any interim analyses and stopping guidelines, including who will have access to these interim results and make the final decision to terminate the trial	16,17
Harms	#22	Plans for collecting, assessing, reporting, and managing solicited and spontaneously reported adverse events and other unintended effects of trial interventions or trial conduct	17,18
Auditing	#23	Frequency and procedures for auditing trial conduct, if any, and whether the process will be independent from investigators and the sponsor	18
**Ethics and dissemination**			
Research ethics approval	#24	Plans for seeking research ethics committee / institutional review board (REC / IRB) approval	18
Protocol amendments	#25	Plans for communicating important protocol modifications (eg, changes to eligibility criteria, outcomes, analyses) to relevant parties (eg, investigators, REC / IRBs, trial participants, trial registries, journals, regulators)	18
Consent or assent	#26a	Who will obtain informed consent or assent from potential trial participants or authorised surrogates, and how (see Item 32)	19
Consent or assent: ancillary studies	#26b	Additional consent provisions for collection and use of participant data and biological specimens in ancillary studies, if applicable	19
Confidentiality	#27	How personal information about potential and enrolled participants will be collected, shared, and maintained in order to protect confidentiality before, during, and after the trial	19
Declaration of interests	#28	Financial and other competing interests for principal investigators for the overall trial and each study site	20,21

Coulson AB, *et al. BMJ Open* 2022; 12:e056147. doi: 10.1136/bmjopen-2021-056147

Myeloma XIV Supplementary Material to Protocol Paper
EudraCT number: **2018-003590-10**

Data access	#29	Statement of who will have access to the final trial dataset, and disclosure of contractual agreements that limit such access for investigators	19
Ancillary and post trial care	#30	Provisions, if any, for ancillary and post-trial care, and for compensation to those who suffer harm from trial participation	19
Dissemination policy: trial results	#31a	Plans for investigators and sponsor to communicate trial results to participants, healthcare professionals, the public, and other relevant groups (eg, via publication, reporting in results databases, or other data sharing arrangements), including any publication restrictions	19
Dissemination policy: authorship	#31b	Authorship eligibility guidelines and any intended use of professional writers	19
Dissemination policy: reproducible research	#31c	Plans, if any, for granting public access to the full protocol, participant-level dataset, and statistical code	19
**Appendices**			
Informed consent materials	#32	Model consent form and other related documentation given to participants and authorised surrogates	15 (Supplementary Material)
Biological specimens	#33	Plans for collection, laboratory evaluation, and storage of biological specimens for genetic or molecular analysis in the current trial and for future use in ancillary studies, if applicable	21 (Supplementary Material)

None The SPIRIT Explanation and Elaboration paper is distributed under the terms of the Creative Commons Attribution License CC-BY-NC. This checklist can be completed online using https://www.goodreports.org/, a tool made by the EQUATOR Network in collaboration with Penelope.ai

Myeloma XIV Supplementary Material to Protocol Paper
EudraCT number: **2018-003590-10**

Renal and Liver Function

As per standard of care, doses of lenalidomide and ixazomib should be adjusted at baseline according to liver and renal function. Regardless of which dosing arm has been assigned the doses indicated in this section should not be exceeded on cycle 1 day 1. Doses of lenalidomide reduced (based on renal function) can only be escalated, if tolerated, to the maximum dose indicated by the participant's assigned dosing arm/strategy after cycle 1 as per the lenalidomide SPC. Ixazomib doses may **not** be escalated, whether reduced based on renal or liver function.

Renal function:

Creatinine clearance should be calculated using the Cockcroft-Gault formula as the estimated GFR produced in most hospitals is not accurate in older patients. For moderate renal impairment, ixazomib should be given 4mg daily and should be reduced be reduced to 3mg in patients with severe renal impairment and end of stage renal disease. For moderate renal impairment, 10mg of Lenalidomide once daily should be given, 15 mg every other day for severe renal impairment and 5mg once daily at end stage renal disease.

	**Calculated creatinine Clearance (using Cockcroft-Gault formula)**	**Lenalidomide dose** (Days 1-21)	**Ixazomib dose** (Day 1, 8, 15)
Moderate renal impairment	≥ 30 to < 50 ml/min	10mg once daily	4mg once daily
Severe renal impairment	< 30 ml/min; not requiring dialysis	15mg every other day	3mg once daily
End stage renal disease	< 30 ml/min; requiring dialysis	5mg once daily (on dialysis days should be administered following dialysis)	3mg once daily

Liver function:

The reduced ixazomib dose of 3 mg is recommended in patients with moderate liver dysfunction characterised by total bilirubin > 1.5-3 x ULN (patients with bilirubin > 3 x ULN are not eligible for the study). No dose reduction based on AST/ALT is required.

Lenalidomide has not formally been studied in patients with impaired hepatic function and there are no specific dose recommendations

Coulson AB, *et al. BMJ Open* 2022; 12:e056147. doi: 10.1136/bmjopen-2021-056147

Myeloma XIV Supplementary Material to Protocol Paper
EudraCT number: **2018-003590-10**

Concomitant Medication

**Permitted Concomitant Medication:**
1) Antiemetics, including 5-HT3 serotonin receptor antagonists, may be used at the discretion of the investigator (however, note that dexamethasone additional to that given as part of the trial treatment schedule is **not** permitted).<br>2) Loperamide or other anti-diarrhoeal should be used for symptomatic non-infective diarrhoea at the discretion of the investigator. The dose and regimen will be according to institutional guidelines. Intravenous fluids should be given to prevent volume depletion.<br>3) Growth factors (e.g. granulocyte colony stimulating factor [G-CSF], thrombopoeitin receptor agonists (TPO) and recombinant erythropoietin) are permitted at the discretion of the PI and/or institutional practice. Due consideration of the increased risk of VTE with TPO agonists and erythropoietin should be given and prophylaxis adjusted accordingly.<br>4) Participants should be transfused with red cells and platelets as clinically indicated and according to institutional guidelines. Platelet transfusions are not allowed within 3 days prior to study drug dosing for any dosing day in order to meet pre-treatment criteria, to ensure that the platelet count is at a safe level to commence further treatment.<br>5) Patients known to be Hep B core antibody positive should receive prophylaxis with an appropriate anti-viral as per local protocols and after consultation with a gastroenterology/hepatology specialist.<br>6) Other supportive measures consistent with optimal participant care may be given throughout the study.
**Excluded concomitant Medication:**
1) Any antineoplastic treatment with activity against MM, other than study drugs and localised radiotherapy for pain relief.<br>2) Platelet transfusions within 3 days prior to study drug dosing for any dosing day in order to meet pre-treatment criteria, or prior to randomisation in order to meet eligibility criteria.<br>3) Additional steroid therapy (not as trial IMP) (except: before commencing trial treatment up to a maximum of 160 mg dexamethasone or equivalent based on glucocorticoid activity per inclusion criteria, or for the management of rash in which case topical, IC or oral steroid at a dose not exceeding prednisolone 10mg per day or equivalent is permitted)<br>4) Radiotherapy for the treatment of symptoms related to disease progression (in the event of disease progression participant should stop trial treatment and further treatment will be at the discretion of the treating clinician).<br>5) Excluded foods and dietary supplements include St. John's Wort.<br>6) Live vaccines are prohibited 30 days prior to R1 and for the duration of the study. They are also prohibited for 3 months after the last dose of study drug.<br>The following treatments should be avoided, unless deemed necessary by the treating clinician:<br>1) Systemic treatment with any of the following metabolising enzyme inducers should be avoided, unless there is no appropriate alternative medication for the participant's use (Rationale: If there were to be a drug-drug interaction with an inducer, ixazomib exposure would be decreased):<br>2) Strong CYP3A inducers: rifampicin, rifabutin, carbamazepine, phenytoin, & phenobarbital<br>3) Non-steroidal anti-inflammatory drugs (NSAIDs) should be avoided especially in those with abnormal renal function unless the clinical benefit outweighs the risk to deteriorating renal impairment.

Coulson AB, *et al. BMJ Open* 2022; 12:e056147. doi: 10.1136/bmjopen-2021-056147

Myeloma XIV Supplementary Material to Protocol Paper
EudraCT number: **2018-003590-10**

Exploratory analysis

Exploratory analysis will consider; the association of molecular subgroups with outcome through the investigation of frailty biomarkers, differences between the Karnofsky Performance Status (PS) and ECOG PS, and the prospective validation the UK-MRA MRP (United Kingdom-Myeloma Research Alliance Myeloma Risk Profile). All exploratory analysis to be conducted by the trial statisticians will be pre-specified in the statistical analysis plan.

Subgroup analysis

A series of subgroup analyses will be conducted to determine whether a selection of patient characteristics, haematology / local serological results, biochemistry results and cytogenetic/molecular results as well as response assessments are prognostic of the endpoints; Progression-Free survival, Progression-free survival 2, overall survival, attainment of ≥VGPR (Very Good Partial Response) and attainment of Minimal Residual Disease (MRD) negativity, as defined for both randomisations. Note that the subgroups MRD response to IRD (Ixazomib, Lenalidomide and Dexamethasone) induction and MRD response at 12m maintenance will not be analysed for the attainment of MRD negativity endpoint.

Patient Characteristics

- Age at R1 baseline (<70,70-80,>80)
- Sex (Male / Female)
- ECOG performance status at R1 baseline (0,1, ≥2, missing)
- Karnofsky performance status at R1 baseline (<80, 80-90,>90, missing)
- ISS at R1 baseline (Stage I, Stage II, Stage III, missing)
- R-ISS at R1 baseline (Stage I, Stage II, Stage III, missing)
- IMWG frailty category at R1 baseline (Fit, Unfit, Frail)
- MRP frailty category at R1 baseline (Fit, Intermediate-Fitness, Frail, missing)

Hematology / Local Serological Results

- Haemaglobin concentration at R1 baseline (<100, ≥100 g/L)
- Platelets at R1 baseline (<150, ≥150 x109/L)
- White blood cells at R1 baseline (<LLN, ≥LLN, missing)
- Neutrophil count at R1 baseline (<LLN, ≥LLN)
- Lymphocyte count at R1 baseline (<LLN, ≥LLN, missing)
- Plasma cells (%) in bone marrow at R1 baseline (<60%, ≥60%)

Biochemistry Results

- β-2 microglobulin concentration at R1 baseline (<3.5, 3.5–<5.5, ≥5.5mg/L)
- Serum creatinine concentration at R1 baseline (<175, ≥175 µmol/L)
- Corrected serum calcium concentration at R1 baseline (<2.75, ≥2.75 mmol/L)
- Serum albumin at R1 baseline (<ULN, ≥ULN, missing)
- Lactate dehydrogenase at R1 baseline (<ULN, ≥ULN, missing)
- C-Reactive protein at R1 baseline (<ULN, ≥ULN, missing)
- Total bilirubin at R1 baseline (<ULN, ≥ULN)
- ALT/AST at R1 baseline (<ULN, ≥ULN)

Coulson AB, *et al. BMJ Open* 2022; 12:e056147. doi: 10.1136/bmjopen-2021-056147

Myeloma XIV Supplementary Material to Protocol Paper
EudraCT number: **2018-003590-10**

MLPA Cytogenetic Results

- t(4,14) at R1 baseline (detected, not detected)
- t(14,16) at R1 baseline (detected, not detected)
- t(14,20) at R1 baseline (detected, not detected)
- del(17p) at R1 baseline (detected, not detected)
- del(13q) at R1 baseline (detected, not detected)
- del(1p) at R1 baseline (detected, not detected)
- gain(1q) at R1 baseline (detected, not detected)
- UK definition risk group at R1 baseline (Standard, high risk, ultra-high risk)
- IMWG definition risk group at R1 baseline (Adverse, Standard)

Response

- Response to IRD induction (<VGPR, ≥ VGPR)
- Response to 12m maintenance (<VGPR, ≥ VGPR)
- MRD response to IRD induction (MRD positive, MRD Negative)
- MRD response to 12m maintenance (MRD positive, MRD Negative)

Further subgroup analyses may be undertaken, as appropriate and all subgroup analysis will be fully documented in the statistical analysis plan. Subgroup and exploratory analyses may, by chance, generate false negative or positive results. Those carried out will be interpreted with caution.

Myeloma XIV Supplementary Material to Protocol Paper
EudraCT number: **2018-003590-10**

Informed Consent Material

Bone marrow and Blood sample consent form

Delete this line, then print on Trust/Hospital headed paper

Participant ID:	Initials:
Date of Birth:	NHS/Hospital Number:
EudraCT Number:	Principal Investigator:

FiTNEss
UK-MRA Myeloma XIV

PARTICIPANT CONSENT FORM FOR BONE MARROW AND BLOOD SAMPLE

Please initial each box

1. I confirm that I have read and understand the information sheet for the bone marrow and blood sample request and have had the opportunity to ask questions.
2. I understand that my providing bone marrow and blood samples is voluntary and that I am free to withdraw at any time without my medical care or legal rights being affected.
3. I understand that the samples will be sent along with information about me, which will include NHS number and may include my name.
4. I agree to these samples being stored and used for research investigations that form part of the FiTNEss (UK-MRA Myeloma XIV) study, and understand that this will include genetic research.
5. I understand that a unique reference number will be allocated to the samples which may allow them to be linked back to me in future for research purposes.
6. I understand that my data may be shared on a collaborative basis with researchers in the UK and potentially, centres abroad, including outside the European Economic Area.

Coulson AB, *et al. BMJ Open* 2022; 12:e056147. doi: 10.1136/bmjopen-2021-056147

7. I agree to allow any information provided to be used for healthcare and/or further medical research upon the understanding that my identity will remain anonymous wherever possible. ☐

8. I understand that results of my laboratory tests may be shared with other research teams if I am taking part in another clinical study being run by the University of Leeds. ☐

9. I agree to a copy of this Consent Form being sent to the Clinical Trials Research Unit. ☐

10. I agree for my details (which will include date of birth and NHS number) to be submitted to NHS Digital so that information about my health, and mortality should this become available, may be obtained by the CTRU if necessary. ☐

11. I agree to provide bone marrow and blood samples. ☐

Please indicate your wishes in the below scenarios:

Please tick or initial yes or no:

Please tick ✓ or initial

	Yes	No
A) If I am diagnosed with a plasma cell dyscrasia, other than myeloma, I give permission for the samples sent to the central laboratories to be stored and used in future research that receives ethical approval. I understand that the samples and data collected from them may be shared with researchers, possibly outside the EEA.	☐	☐
B) If I am diagnosed with myeloma but do not take part in the FiTNEss (UK-MRA Myeloma XIV) study, I give permission for the samples sent to the central laboratories to be stored and used in future research that receives ethical approval. I understand that the samples and data collected from them may be shared with researchers, possibly outside the EEA.	☐	☐

Patient (to be completed by the patient):

Signature……..

Name (block capitals)…………………………………………………………………………………………..

Date………....

Investigator:

I have explained the request to the above named patient and he/she has indicated his/her willingness for samples of bone marrow and blood to be sent to the FiTNEss central laboratories.

Signature……

Name (block capitals)…………………………………………………………………………………………..

Date……

(If used)Translator:

Signature……

Name (block capitals)…………………………………………………………………………………………..

Date……

(1 copy for patient; 1 for the CTRU; 1 held in patient notes, original stored in Investigator Site File)

.

Myeloma XIV Supplementary Material to Protocol Paper
EudraCT number: **2018-003590-10**

Main Trial consent

Delete this line, then print on Trust/Hospital headed paper

Participant ID:	Initials:
Date of Birth:	NHS/Hospital Number:
EudraCT Number:	Principal Investigator:

FiTNEss
UK-MRA Myeloma XIV

PARTICIPANT CONSENT FORM

Please initial each box

1. I confirm that I have read and understand the information sheet for the above study and have had the opportunity to ask questions. ☐

2. I understand that my participation in this study is voluntary and that I am free to withdraw at any time without my medical care or legal rights being affected. I understand that even if I withdraw from the above study, the data and samples collected from me will be used in analysing the results of the study, and in some cases further information about any unwanted effects of my treatment may need to be collected by the study team. ☐

3. I understand that my healthcare records may be looked at by authorised individuals from the study team, regulatory bodies or Sponsor in order to check that the study is being carried out correctly. ☐

4. I understand that I will be asked questions about how independent my lifestyle is. I understand that this information will be used to generate a score of frailty and may affect the doses of treatment that I receive. ☐

5. I am aware that I am required to have samples taken and sent to a central laboratory and I understand that this will include genetic research. I understand that the samples will be sent along with ☐

Coulson AB, *et al. BMJ Open* 2022; 12:e056147. doi: 10.1136/bmjopen-2021-056147

information about me, which will include NHS number and may include my name.

6. I understand that results or reports from imaging scans, blood tests or other investigations (including for additional cancers) may be sent to the Clinical Trials Research Unit for central review by the research team. ☐

7. I agree to allow any information or results arising from this study to be used for healthcare and/or further medical research upon the understanding that my identity will remain anonymous wherever possible. ☐

8. I understand that my data and results of my laboratory tests (blood, urine and bone marrow sample collections) may be shared with other research teams if I am taking part in another clinical study being run by the University of Leeds. ☐

9. I agree for my details (which will include date of birth and NHS number) to be submitted to NHS Digital so that information about my health, and mortality should this become available, may be obtained by the CTRU if necessary. ☐

10. I understand that my GP, or any other doctor treating me, will be notified of my participation in this study. ☐

11. I understand that some of my data may be passed to other organisations, such as the pharmaceutical companies Takeda and Celgene, (possibly in other countries outside the EEA where the data protection standards and laws may be different from the UK) to monitor the safety of the treatment(s) that I am receiving. I understand that my identity will remain anonymous. ☐

12. I agree to a copy of this Consent Form being sent to the Clinical Trials Research Unit ☐

13. I agree to take part in the study. ☐

Myeloma XIV Supplementary Material to Protocol Paper
EudraCT number: **2018-003590-10**

The following parts of the study are OPTIONAL (see part 3 of the Participant Information Sheet). Even if you agree to take part in the FiTNEss study, you do not have to agree to these optional parts. Please tick or initial yes or no.

Please tick ✓ or initial

	Yes	No
A) I agree to take part in the Quality of Life and Healthcare Resource Use studies by completing questionnaires at time points throughout the study.	☐	☐
B) I give permission for samples that I provide to be used in future research that receives ethical approval, and I understand that this includes the blood and bone marrow samples provided at the time of my diagnosis (if consented separately to the bone marrow and blood sample for this study). I understand that my samples and data collected from them may be shared on a collaborative basis with researchers in the UK and potentially, centres abroad, including outside the European Economic Area.	☐	☐

Patient (to be completed by the patient):

Signature………………………………………………………………………………

Name (block capitals)……………………………………………………………………

Date………………………………………………………………………………………

Investigator:

I have explained the study to the above named patient and he/she has indicated his/her willingness to participate.

Signature………………………………………………………………………………

Name (block capitals)……………………………………………………………………

Date………………………………………………………………………………………

(If used)Translator:

Signature………………………………………………………………………………

Name (block capitals)……………………………………………………………………

Date………………………………………………………………………………………

Coulson AB, *et al. BMJ Open* 2022; 12:e056147. doi: 10.1136/bmjopen-2021-056147

Myeloma XIV Supplementary Material to Protocol Paper
EudraCT number: **2018-003590-10**

Central Sample Analysis

The trial has four central laboratories, each receiving samples for trial purposes. The time points and the reason for analysis for each central sample can be found in Figure 4. Details on the analysis of each sample is below. Note that participants can consent for their samples to be used in future research and hence each laboratory can store their trial samples for use in future studies. The storage details are also included below.

Birmingham

Samples are sent in approved packaging via the UK post office service and received in a UKAS accredited clinical laboratory for laboratory assessment and storage under the direction of the TMG. Assessment of prognostic markers, markers of disease activity and M-protein levels. Analyses will include serum levels of beta-2 microglobulin, immunoglobulins IgG, IgA and IgM, kappa and lambda free immunoglobulin light chains. Serum electrophoresis and immunofixation, M-protein quantitation including total protein and densitometry. Urine immunofixation and free light chain quantitation will be performed where appropriate.

HMDS

The presence of minimal residual disease will be assessed in bone marrow aspirates using a validated flow cytometry assay (sensitivity ≤0.004%) performed at a single central laboratory (HMDS, Leeds Teaching Hospitals Trust). A minimum of 500,000 cells will be evaluated with six- or eight-colour antibody combinations including CD138, CD38, CD45, CD19, CD56, CD27, CD81 and CD117.

Sustained MRD-negativity, as defined by the IMWG [1], will not be assessed as it requires the participant to be imaging MRD-negative at the same timepoints that they are flow MRD-negative and imaging is only mandated at baseline and to investigate new systems not at regular intervals for all participants.

Flow MRD will be assessed at the following time points: Baseline (pre-randomisation 1), after 6 cycles of induction therapy, at the end of 12 cycles of induction treatment (or at the end of the final cycle of IRD treatment if sooner), at 12 months post maintenance randomisation 2. Flow MRD will also be assessed at any point a first occurrence of CR, or SCR is suspected.

During final analysis, we will determine the conversion rate of patients who are MRD Positive at the end of induction to MRD Negative at 12 months post Randomisation 2. The analysis to this is specified within our Statistical Analysis Plan (SAP).

Samples will be used for trial purposes only and will not be stored for future research.

ICR

Myeloma tumor material is purified from fresh bone marrow material using CD138 immunomagnetic separation. Tumor DNA and RNA are extracted and recurrent immunoglobulin translocations t(4;14), t(14;16), t(14;20) and copy number aberrations del(1p32), gain(1q21), del(17p) investigated as previously published using qRT-PCR and multiplex ligation-dependent amplification (MLPA), respectively[2]. Excess material is stored, if patient gave written consent for use in future research, at -80C standard DNA and RNA storage conditions.

LIMR

Myeloma XIV Supplementary Material to Protocol Paper
EudraCT number: **2018-003590-10**

Peripheral blood samples for immunosenescent and Senescence-associated secretory proteome will be draw and managed timepoints in the clinical pathway, processed in a central laboratory and stored as aliquots of mononuclear cells and serum for subsequent planned use. Full QA is performed on sample receipt to ensure only viable and valuable samples are processed and stored for research use.

Myeloma XIV Supplementary Material to Protocol Paper
EudraCT number: **2018-003590-10**

References

1. Kumar, S.P., et al., *International Myeloma Working Group consensus criteria for response and minimal residual disease assessment in multiple myeloma.* The lancet oncology, 2016. **17**(8): p. e328-e346.
2. Shah, V., et al., *Prediction of outcome in newly diagnosed myeloma: a meta-analysis of the molecular profiles of 1905 trial patients.* Leukemia, 2018. **32**(1): p. 102-110.

Coulson AB, *et al. BMJ Open* 2022; 12:e056147. doi: 10.1136/bmjopen-2021-056147
