# Supplementary material for: Frailty-adjusted therapy in Transplant Non-Eligible patients with newly diagnosed Multiple Myeloma (FiTNEss (UK-MRA Myeloma XIV Trial)): a study protocol for a randomised phase III trial
Source: BMJ Open. 2022 Jun 2;12(6):e056147. doi: 10.1136/bmjopen-2021-056147 (PMC9163533; doi:10.1136/bmjopen-2021-056147)
Supplement: Author's manuscript [file bmjopen-2021-056147.draft_revisions.pdf]

BMJ Open is committed to open peer review. As part of this commitment we make the peer review history of every article we publish publicly available.

When an article is published we post the peer reviewers' comments and the authors' responses online. We also post the versions of the paper that were used during peer review. These are the versions that the peer review comments apply to.

The versions of the paper that follow are the versions that were submitted during the peer review process. They are not the versions of record or the final published versions. They should not be cited or distributed as the published version of this manuscript.

BMJ Open is an open access journal and the full, final, typeset and author-corrected version of record of the manuscript is available on our site with no access controls, subscription charges or pay-per-view fees (http://bmjopen.bmj.com).

If you have any questions on BMJ Open's open peer review process please email info.bmjopen@bmj.com

BMJ Open

Frailty-adjusted therapy in Transplant Non-Eligible patients with newly diagnosed Multiple Myeloma (FiTNEss (UK-MRA Myeloma XIV Trial)): A study protocol for a randomised phase III trial

Journal:	*BMJ Open*
Manuscript ID	bmjopen-2021-056147
Article Type:	Protocol
Date Submitted by the Author:	18-Aug-2021

Complete List of Authors:	Coulson, Amy; University of Leeds Clinical Trials Research Unit, Leeds Institute of Clinical Trials Research<br>Royle, Kara-Louise; University of Leeds Clinical Trials Research Unit, Leeds Institute of Clinical Trials Research<br>Pawlyn, Charlotte; Cancer Research UK London Research Institute<br>Cairns, David; University of Leeds Clinical Trials Research Unit, Leeds Institute of Clinical Trials Research<br>Hockaday, Anna; University of Leeds Clinical Trials Research Unit, Leeds Institute of Clinical Trials Research<br>Bird, Jennifer; University Hospitals Bristol and Weston NHS Foundation Trust, Department of Haematology<br>Bowcock, Stella; Princess Royal Hospital, Department of Haematology, Kings college Hospital NHS Foundation Trust<br>Kaiser, Martin; Institute of Cancer Research; Royal Marsden Hospital NHS Trust, The Department of Haemato-oncology<br>de Tute, Ruth; University of Leeds, Haematology Malignancy Diagnostic Service (HMDS)<br>Rabin, Neil; University College London Hospitals NHS Foundation Trust, Department of Haematology<br>Boyd, Kevin; Royal Marsden Hospital NHS Trust, The Department of Haemato-oncology<br>Jones, John; King's College Hospital; Eastbourne District General Hospital<br>Parrish, Christopher; Leeds Teaching Hospitals NHS Trust, Department of Haematology<br>Gardner, Hayley; University Hospitals Birmingham NHS Foundation Trust, Department of Haematology and Stem Cell Transplantation<br>Meads, David; University of Leeds Leeds Institute of Health Sciences, Academic Unit of Health Economics<br>Dawkins, Bryony ; University of Leeds Leeds Institute of Health Sciences, Academic Unit of Health Economics<br>Olivier, Catherine; University of Leeds Clinical Trials Research Unit, Leeds Institute of Clinical Trials Research<br>Henderson, Rowena; University of Leeds Clinical Trials Research Unit, Leeds Institute of Clinical Trials Research<br>Best, Phillip; University of Leeds Clinical Trials Research Unit, Leeds Institute of Clinical Trials Research<br>Owen, Roger; St James's University Hospital, Haematology Malignancy

	Diagnostic Service (HMDS)<br>Jenner, Matthew ; Southampton General Hospital<br>Kishore, Bhuvan; University Hospitals Birmingham NHS Foundation Trust, Department of Haematology and Stem Cell Transplantation,<br>Drayson, Mark ; University of Birmingham, Institute of Immunology and Immunotherapy University of Birmingham Department of Haematology<br>Jackson, Graham; Newcastle University, Department of Haematology<br>Cook, Gordon; University of Leeds Clinical Trials Research Unit, Leeds Institute of Clinical Trials Research; St James's University Hospital, Leeds Cancer Centre

Keywords:	Myeloma < HAEMATOLOGY, Myeloma < ONCOLOGY, CHEMOTHERAPY, Clinical trials < THERAPEUTICS

SCHOLARONE™
Manuscripts

Frailty-adjusted therapy in Transplant Non-Eligible patients with newly diagnosed Multiple Myeloma (FiTNEss (UK-MRA Myeloma XIV Trial)): A study protocol for a randomised phase III trial

Amy Beth Coulson[1], Kara-Louise Royle[1], Charlotte Pawlyn[2], David A Cairns[1], Anna Hockaday[1], Jennifer Bird[3], Stella Bowcock[4], Martin Kaiser[2,5], Ruth De Tute[6], Neil Rabin[7], Kevin Boyd[5], John Jones[15,16,17], Christopher Parrish[14], Hayley Gardner[9], David Meads[10], Bryony Dawkins[10], Catherine Olivier[1], Rowena Henderson[1], Phillip Best[1], Roger Owen[6], Matthew Jenner[11], Bhuvan Kishore[9], Mark Drayson[12], Graham Jackson[13] and Gordon Cook[1,8*]

1. Clinical Trials Research Unit, Leeds Institute of Clinical Trials Research, University of Leeds, Leeds, United Kingdom
2. The Institute of Cancer Research, London, United Kingdom
3. Department of Haematology, University Hospitals Bristol and Weston NHS Trust , Bristol, UK
4. Department of Haematology, King's College Hospital NHS Foundation Trust, Princess Royal Hospital, London, UK
5. The Department of Haemato-oncology, The Royal Marsden NHS Foundation Trust, London, UK
6. HMDS, St James's University Hospital, Leeds, UK
7. The Department of Haematology, University College London Hospitals NHS Trust, London
8. Leeds Cancer Centre, St James's University Hospital, Leeds, United Kingdom
9. Department of Haematology and Stem Cell Transplantation, University Hospitals Birmingham NHS Foundation Trust, Birmingham
10. Academic Unit of Health Economics, Leeds Institute of Health Sciences, University of Leeds, Leeds, UK
11. University Hospital Southampton NHS Foundation Trust
12. Institute of Immunology and Immunotherapy University of Birmingham Department of Haematology
13. Department of Haematology, Newcastle University, Newcastle-upon-Tyne, United Kingdom
14. Department of Haematology, Leeds Teaching Hospitals NHS Trust, Leeds, UK
15. Brighton and Sussex Medical School, Brighton, UK
16. King's College Hospital, London, UK.
17. Eastbourne District General Hospital, UK.

*Corresponding Author:

Gordon Cook, MB.ChB, PhD, FRCP(Glas), FRCPath

Leeds Cancer Centre

St James's University Hospital

Beckett Street, Leeds, UK, LS9 7TF

Tel: +44 113 206 7940 Fax: +44 113 206 8177

E-mail: g.cook@leeds.ac.uk

Word count: 6558

For peer review only - http://bmjopen.bmj.com/site/about/guidelines.xhtml

Abstract

Introduction

Multiple myeloma is a bone marrow cancer which predominantly affects older people. The incidence is increasing in an ageing population.

Over the last ten years patient outcomes have improved. However, this is less apparent in older, less fit patients, who are ineligible for stem cell transplant. Research is required in this patient group, taking into account frailty and aiming to improve: treatment tolerability, clinical outcomes and quality of life.

Methods and analysis

FiTNEss is a national, phase III, multicentre, randomised controlled trial comparing standard (reactive) and frailty-adjusted (adaptive) induction therapy delivery with ixazomib, lenalidomide and dexamethasone (IRD), and to compare maintenance lenalidomide to lenalidomide plus ixazomib, in patients with newly diagnosed multiple myeloma not suitable for stem cell transplant. Seven-Hundred and Forty participants will be registered into the trial to allow 720 and 478 to be randomised at induction and maintenance, respectively.

All participants will receive IRD induction with the dosing strategy randomised (1:1) at trial entry. Patients randomised to the standard, reactive arm will commence at the full dose followed by toxicity dependent reactive modifications. Patients randomised to the adaptive arm will commence at a dose level determined by their International Myeloma Working Group (IMWG) frailty score. Following 12 cycles of induction treatment participants alive and progression-free will undergo a second (double-blind) randomisation on a 1:1 basis to maintenance treatment with lenalidomide plus placebo versus lenalidomide plus ixazomib until disease progression or intolerance.

Ethics and dissemination

Ethical approval has been obtained from the North East - Tyne & Wear South Research Ethics Committee (19/NE/0125) and capacity and capability confirmed by local research and development departments for each participating centre prior to opening to recruitment. Participants are required to provide written informed consent prior to trial registration. Trial results will be disseminated by conference presentations and peer-reviewed publications.

Trial registration

ISRCTN17973108

ClinicalTrials.gov:NCT03720041.

Key Words: Multiple Myeloma, Transplant-ineligible, Frailty-adjusted, Randomised, Blinded

For peer review only - http://bmjopen.bmj.com/site/about/guidelines.xhtml

Article Summary

Strengths and limitations of this Study

- FiTNEss will provide the first prospective data investigating the use of the International Myeloma Working Group (IMWG) frailty score to define appropriate dose delivery strategies for older patients.
- FiTNEss will explore the impact of dual agent maintenance compared to the single agent standard of care using a gold standard placebo controlled design.
- The trial has the potential to meet a high unmet need in older myeloma patients for whom the impact of recent therapeutic have been less marked.
- Wide inclusion criteria have been designed to maximise the recruitment of older, more frail patients who may not previously have been included in clinical trials.
- Owing to the nature of assessments and dose adaptations blinding in the induction phase of the trial is infeasible.

For peer review only

For peer review only - http://bmjopen.bmj.com/site/about/guidelines.xhtml

Introduction

Multiple Myeloma

Multiple Myeloma is the second most common haematological malignancy with over 5500 patients diagnosed in the UK each year.[1] Myeloma is predominantly a disease of older people, with two thirds of patients aged over 70 years at diagnosis. The incidence is increasing as the population ages.

Over the last ten years the development of proteasome inhibitors (PI), immunomodulatory drugs (IMiD® agents) and improved supportive care, have ameliorated outcomes for myeloma patients such that the median overall survival is now more than 6 years for younger, fitter patients (Figure 1A).[2] However, the impact of these therapies has been less marked in the older or less fit population, particularly those over 75 years of age and/or ineligible for stem cell transplant (Figure 1B). Whilst outcomes in younger patients are largely driven by molecular risk factors present in the myeloma cell clone, there is no evidence that older myeloma patients have more biologically high-risk disease.[3] Differences in outcomes are likely to be accounted for by changes in patient physiology and/or increased treatment-related toxicity. Data from our previous trial, Myeloma XI (ISRCTN49407852) shows that as age increases the number of participants ceasing treatment due to choice or toxicity increases (Figure 2). This group therefore has a high unmet need for new, less toxic treatments and improved treatment delivery approaches.

Existing Evidence: Induction Therapy

Treatment

Lenalidomide is an immunomodulatory agent and thalidomide derivative available as an oral preparation, which is more potent *in vitro* and with a different adverse effect profile than thalidomide. For example, a major benefit of lenalidomide is the absence of associated neurotoxicity or sedation seen with thalidomide, making it more tolerable; however, there is a significant rate of myelosuppression (20%), which is not seen with thalidomide. Lenalidomide is licensed, in combination with dexamethasone, for use in newly diagnosed multiple myeloma patients who are not eligible for transplant (TNE).

With the caveats of cross-trial comparison, our recent analysis of the Myeloma XI trial suggests that the combination CRDa (cyclophosphamide, lenalidomide and attenuated dexamethasone) is not superior to Rd-continuous used in the FIRST study (NCT00689936) in terms of progression-free survival (PFS). In addition, Myeloma XI also showed that patients in the non-intensive pathway with only a minimal or partial response to induction therapy who were randomised to receive cyclophosphamide, bortezomib and dexamethasone (CVD) consolidation had better outcomes than those who did not (an increase in median PFS of 11 months (HR: 0.73; P=0.061)).[4] However, a significant proportion of patients (394/610; 64.6%) did not make it to this randomisation due to participant withdrawal (211; 53.6% of 394), death (96; 24.4%), progression (45; 11.4%) and other reasons (42; 10.7%). These data suggest that combining IMiD® agents and PI upfront may improve outcomes further, avoiding the early loss of patients and leading to improved responses, as we have seen with the carfilzomib, cyclophosphamide, lenalidomide and dexamethasone (KCRD) arm in the transplant-eligible pathway of the Myeloma XI study.[5,6]

Ixazomib (MLN9708) is an orally bioavailable, small molecule inhibitor of the 20S proteasome, and has shown single agent activity in phase I/II studies alongside combination therapy with dexamethasone and more recently IMiD agents and alkylating agents.[7-9] The oral formulation provides convenience

For peer review only - http://bmjopen.bmj.com/site/about/guidelines.xhtml

for patients, and the slower pharmacokinetic (PK) profile reduces the neurotoxicity seen with bortezomib, suggesting the use of this new PI in combination regimens might be better tolerated.[10] In the front-line setting the combination of ixazomib with lenalidomide and dexamethasone (IRD) has been reported in phase I/II studies demonstrating high response rates and good tolerability.[11]

With a favourable toxicity profile compared to either carfilzomib or bortezomib, and the benefits of oral dosing, the IRD combination represents a tolerable regimen to achieve the same combination of IMiD® agents and PI in TNE patients. The Tourmaline MM-1 study demonstrated excellent efficacy and tolerability of IRD in the relapsed setting in TNE patients, supporting this hypothesis.[12] The Tourmaline MM-02 study, a randomised, double blind, placebo controlled study evaluating IRd vs placebo Rd has recently been reported[13] and showed a clinically meaningful but non-significant 13.5 month improvement in PFS in the IRd group (35.3 vs 21.8 months; HR 0.83; P = 0.073). This trial used reactive dosing strategies.

Treatment Delivery

The International Myeloma Working Group (IMWG) proposed a scoring system for myeloma patient frailty that predicts survival, adverse events (AEs) and treatment tolerability,[14] which can help to account for the considerable heterogeneity in outcome for TNE patients. This score combines age and the outcomes of 3 patient assessment tools; the Katz Activity of Daily Living (ADL),[15] Lawton's Instrumental Activity of Daily Living (IADL),[16] and the Charlson Comorbidity Index (CCI)[17 18] to categorise patients into three groups: fit, unfit and frail. The IMWG frailty score was subsequently shown to be predictive of both PFS and toxicity. An increase in frailty score was associated with an increased risk of death, progression, non-haematological AEs and treatment discontinuation that was independent of classical definitions of risk, including ISS stage and cytogenetic risk, and also independent of treatment regimen. As such it was suggested to be useful in determining the feasibility of treatment regimens and appropriate dose reductions, but this remains to be prospectively validated.

Existing Evidence: Maintenance Therapy

Four published studies have demonstrated an important clinical benefit for the use of maintenance lenalidomide in newly diagnosed multiple myeloma in patients of all ages. Data from our previous study Myeloma XI contributes to this evidence base and in TNE patients, lenalidomide maintenance demonstrated a significant improvement in PFS compared to observation, of 24 months vs 11 months from maintenance randomisation.[19] This improvement was seen across all subgroups of multiple myeloma patients including all cytogenetic risk groups and at all ages. Overall survival data demonstrated a benefit for lenalidomide once the effect of subsequent therapies have been taken in to account.[20]

Overall, the data for maintenance lenalidomide until disease progression in patients not eligible for stem cell transplant suggest that there is a clear and significant improvement in PFS and a possible OS benefit. The crucial question to answer, going forward, is whether the results seen with lenalidomide as a single agent for maintenance can be enhanced further by the use of a combination regimen.

The use of ixazomib monotherapy in the maintenance setting demonstrated efficacy and tolerability in previously untreated patients [21] and this has recently been confirmed in the Phase III Tourmaline-MM4 trial in the non-transplant-eligible setting,[22] Adding ixazomib to lenalidomide maintenance has not been studied in a randomised phase III study.

For peer review only - http://bmjopen.bmj.com/site/about/guidelines.xhtml

Existing Evidence: Summary

The recently developed, less-toxic PI ixazomib [11] with novel IMiD® agents/PI combinations for induction and maintenance treatment needs to be evaluated in the context of the IMWG frailty score where frailty-adjusted dosing was recommended,[14] but emphasised the need for prospective validation of their approach.

Aims and Objectives

The FiTNEss trial aims to improve outcomes for TNE patients by investigating whether using prospective dose adjustments dependent on patient frailty will improve patients' ability to remain on therapy, reduce toxicity, and improve outcomes from randomisation 1 (R1). The trial also aims, from randomisation 2 (R2), to investigate whether doublet maintenance therapy improve outcomes compared to single agent lenalidomide without prohibitive toxicity.

Trial Design

The FiTNEss trial is a phase III, multi-centre, randomised, parallel group trial in newly diagnosed patients with MM, who are assessed to be TNE by their treating clinician. Following R2 the trial is also double-blind placebo-controlled with the participant and treating clinician blind to treatment allocation. The following report details the trial protocol and follows the structure of the SPIRIT statement.[23] The SPIRIT checklist[24] can be found within the supplementary material.

Methods

Setting

The trial will be conducted at 87 centres around the United Kingdom (See supplementary material), as identified via a feasibility assessment to determine the most appropriate to participate in the trial. The majority of potential participants will be identified by the research team at the time they are referred to the haematology outpatient department with suspected multiple myeloma. A smaller number of participants may be identified during inpatient admissions. Invitation to participate in the trial and provision of information will be made either during their first consultation, when routine diagnostic tests will be performed and potential treatment options discussed, or at the time they receive their diagnostic test results.

Eligibility criteria

Adults (18 years and older) with newly diagnosed MM, by IMWG 2014 diagnostic criteria, [25] who are TNE and who are capable of giving written informed consent will be assessed for eligibility. Eligibility will be confirmed prior to each randomisation by the principal investigator or authorised delegate and will be recorded in the participant's medical records and on the relevant case report form (CRF). The participant will be registered into the trial prior to undergoing procedures that are specifically for the purposes of the trial and are above NHS standard of care.

To be eligible for R1, participants must meet all the inclusion criteria and none of the exclusion criteria outlined in Table 1. Following 12 cycles of induction therapy, participants who achieve at least a minimal response (MR), according to IMWG uniform response criteria, and fulfill all the inclusion criteria and none of the exclusion criteria outlined in Table 2, will proceed to R2.

For peer review only - http://bmjopen.bmj.com/site/about/guidelines.xhtml

Table 1: Randomisation 1 Inclusion and Exclusion Criteria

Inclusion Criteria:

1. Newly diagnosed as having MM according to the updated IMWG diagnostic criteria 2014 (see Appendix 1 for criteria) requiring treatment.

2. Not eligible for stem cell transplant.

3. Aged at least 18 years.

4. Meet all of the following blood criteria within 14 days before R1:

Haematological:

a) Absolute neutrophil count (ANC) ≥ 1 x 109/L. Unless the participant has a known/suspected diagnosis of familial or racial neutropenia in which case an ANC ≥ 0.75 x 109/L is allowed. The use of growth factor support is permitted.

b) Platelet count ≥ 50 x 109/L, or, in the case of heavy bone marrow infiltration (≥ 50%) which in the opinion of the investigator is the cause of the thrombocytopenia and provided appropriate supportive measures and patient monitoring are in place, platelet count ≥ 30x 109/L is permitted. Please note: Platelet transfusions are not allowed ≤ 3 days prior to randomisation in order to meet these values.

c) Haemoglobin ≥ 80 g/L. The use of red blood cell transfusions is permitted.

Biochemical:

d) Total bilirubin ≤ 3 x upper limit of normal (ULN).

e) Alanine aminotransferase (ALT) and/or aspartate aminotransferase (AST) [illegible] 3 [illegible] ULN.

5. Meet the pregnancy prevention requirements:

Female participants who:

a) Are not of childbearing potential (Appendix 8), OR

b) If they are of childbearing potential, agree to practice 2 effective methods of contraception (Appendix 8), at the same time, from the time of signing the informed consent form until 90 days after the last dose of study drug, OR

c) Agree to practice true abstinence when this is in line with the preferred and usual lifestyle of the subject. (Periodic abstinence [e.g. calendar, ovulation, symptothermal, post-ovulation methods] and withdrawal are not acceptable methods of contraception.)

Male participants, even if surgically sterilised (i.e. status post-vasectomy), must agree to one of the following:

a) Agree to practice effective barrier contraception during the entire study treatment period and through 90 days after the last dose of study drug, OR

b) Agree to practice true abstinence when this is in line with the preferred and usual lifestyle of the subject. (Periodic abstinence (e.g. calendar, ovulation, symptothermal, post-ovulation methods] and withdrawal are not acceptable methods of contraception).

Contraception for female and male participants must be in accordance with (and participants must consent to) the Celgene-approved Pregnancy Prevention Programme.

If female and of childbearing potential (see Appendix 8), they must have a negative pregnancy test performed by a healthcare professional in accordance with the Celgene Pregnancy Prevention Programme.

6. Able to provide written informed consent.

Exclusion Criteria:

1. Smouldering MM, MGUS, solitary plasmacytoma of bone, or extramedullary plasmacytoma (without evidence of MM).

2. Received previous treatment for MM, with the exception of local radiotherapy to relieve bone pain or spinal cord compression, prior bisphosphonate treatment, or corticosteroids as long as the total dose does not exceed the equivalent of 160 mg dexamethasone.

3. Known resistance, intolerance or sensitivity to any component of the planned therapies.

4. Prior or concurrent invasive malignancies except the following:

- Adequately treated basal cell or squamous cell skin cancer;
- Incidental finding of low grade (Gleason 3+3 or less) prostate cancer requiring no intervention;
- Adequately treated carcinoma in situ of the breast or cervix no longer requiring medical or surgical intervention;
- Any cancer from which the subject has been disease-free for at least 3 years.

5. Pregnant, lactating or breastfeeding female participants.

6. Major surgery within 14 days before randomisation. This would include surgical intervention for relief of cord compression but does not include vertebroplasty or kyphoplasty.

For peer review only

7. Systemic treatment, within 14 days before the first dose of ixazomib with strong CYP3A inducers (e.g. rifampicin, rifabutin, carbamazepine, phenytoin, phenobarbital), or use of St. John's wort.

8. Any concomitant drug therapy which, in the opinion of the investigator, may lead to an unacceptable interaction with any of the agents ixazomib, lenalidomide, dexamethasone, and that cannot be safely stopped prior to trial entry. Full details of interactions can be found in the SPCs.

9. Known gastrointestinal (GI) disease or GI procedure that could interfere with the oral absorption or tolerance of trial treatment, including difficulty swallowing.

10. ≥ Grade 2 peripheral neuropathy.

11. Known HIV positive

12. Participant has current or prior hepatitis B surface antigen positive or hepatitis C antibody positive. Participants must have screening conducted within 14 days before R1.

13. Active systemic infection.

14. Any other medical or psychiatric condition which, in the opinion of the investigator, contraindicates the participant's participation in this study.

15. Receipt of live vaccination within 30 days prior to R1.

Table 2: Randomisation 2 Inclusion ad Exclusion Criteria

R2 Inclusion Criteria:

1. Randomised into the FiTNEss (Myeloma XIV) trial and received induction chemotherapy with ixazomib and lenalidomide continued for 12 cycles.

2. Achieved at least MR at the end of IRD induction according to the IMWG Uniform Response Criteria for Multiple Myeloma (see Appendix 2), with no evidence of progression prior to R2.

3. Meet all of the following blood criteria within 14 days before R2:
Haematological:
a) Absolute neutrophil count (ANC) ≥ 1 x 109/L. Unless the participant has a known/suspected diagnosis of familial or racial neutropenia in which case an ANC ≥ 0.75 x 109/L is allowed. The use of growth factor support is permitted.
b) Platelet count ≥ 50 x 109/L. Please note: Platelet transfusions are not allowed ≤ 3 days prior to randomisation in order to meet these values.
c) Haemoglobin ≥ 80 g/L. The use of red blood cell transfusions is permitted.

Biochemical:
d) Total bilirubin ≤ 3 x upper limit of normal (ULN).
e) Alanine aminotransferase (ALT) and/or aspartate aminotransferase (AST) ⍰ 3 ⍰ ULN.

R2 Exclusion Criteria:

1. Received any anti-myeloma therapy other than their randomised trial treatment, with the exception of local radiotherapy to relieve bone pain (in the absence of disease progression), or bisphosphonate treatment.

2. SD or disease progression according to the IMWG Uniform Response Criteria for Multiple Myeloma (see Appendix 2).

3. Known resistance, intolerance or sensitivity to ixazomib or lenalidomide that required cessation of either agent during induction.

4. Developed any malignancy since R1 except the following:

- Adequately treated basal cell or squamous cell skin cancer;
- Incidental finding of low grade (Gleason 3+3 or less) prostate cancer requiring no intervention;
- Adequately treated carcinoma in situ of the breast or cervix no longer requiring medical or surgical intervention.

5. Pregnant, lactating or breastfeeding female participants.

6. Major surgery within 14 days before randomisation. This does not include vertebroplasty or kyphoplasty.

7. Systemic treatment, within 14 days before the first dose of ixazomib with strong CYP3A inducers (e.g. rifampicin, rifabutin, carbamazepine, phenytoin, phenobarbital), or use of St. John's wort.

8. Known gastrointestinal (GI) disease or GI procedure that could interfere with the oral absorption or tolerance of trial treatment, including difficulty swallowing.

9. ≥ Grade 2 peripheral neuropathy, or grade 1 with pain.

10. Known HIV positive

For peer review only

11. Current or known hepatitis B surface antigen positive or hepatitis C antibody positive
12. Active systemic infection.
13. Any other medical or psychiatric condition which, in the opinion of the investigator, contraindicates the participant's continued participation in this study.
14. Receipt of live vaccination within 30 days prior to R1 or receipt of live vaccination at any point during the trial prior to R2.

Interventions and Dosing

Intervention Schedule

The control and experimental interventions for induction (R1) and maintenance (R2) therapy include: lenalidomide, ixazomib, placebo and dexamethosone. At R1, eligible patients will be allocated to one of two interventions with IRD induction; standard up-front dosing followed by toxicity dependent dose modification (reactive), or; frailty score-adjusted up-front dose reductions (adaptive). At R2, eligible patients will be randomised between lenalidomide + ixazomib (R+I) or lenalidomide and placebo maintenance therapy. Table 3 summarises the dosing schedules for R1 and R2.

Table 3: Dosing Schedule[1]

Randomisation 1			
**Treatment**	**Induction – FIT + Induction – standard dosing**	**Induction- Unfit**	**Induction – Frail**
**Lenalidomide (Days = 1-21)**	25mg	15mg	10mg
**Ixazomib (Days = 1,8, 15)**	4mg	4mg	4mg
**Dexamethasone **(Days = 1,8.15, 22)**	40mg in participants <= 75yrs<br>20 mg in participants >75yrs	20mg	10mg
**Randomisation 2**			
**Treatment**	**R+ Placebo Maintenance**	**R + I maintenance**	
**Lenalidomide (Days=1-21)**	10mg**	10mg**	
**Ixazomib (Days=1,8.15)**	N/A	4mg**	
**Placebo (Days=1,8, 15)**	4mg**	N/A	

*Age is consistently used as the participant's age at cycle 1 day 1.
**or final dose administrated at the end of induction treatment if lower

As part of their induction therapy, participants in the adaptive arm of R1 will have their dose adjusted according to changes in frailty category at the start of cycles 3, 5 and 7. Doses can also be escalated for suboptimal responders under certain criteria. If after cycle 2, a participant on the unfit or frail dosing strategy has not achieved at least an MR, or required a dose reduction due to toxicity, a request can be made to increase any of their doses to the next highest level at the start of cycle 4. A similar

[1] As per standard care, irrespective of R1 allocation, doses of Lenalidomide and Ixazomib will be adjusted at R1 baseline according to liver and renal function. Ixazomib is recommended to be reduced to 3mg in patients with moderate liver dysfunction and with severe renal impairment and end of stage renal disease. No specific dose recommendation have been made for Lenalidomide, however the dose will be as follows: 10mg once daily for moderate renal impairment, 15 mg every other day for severe renal impairment and 5mg once daily at end stage renal disease. Should the dose be tolerated, only the dose of Lenalidomide may be escalated to the maximum dose indicated by the participant's assigned dosing arm/strategy after cycle 1 as per the Lenalidomide SPC.

Ixazomib was not used in general multiple myeloma practice at the time of the EMN (European Myeloma Network) publication. The following licensed dose of Ixazomib will be at induction used for both randomisation arms: 4mg, at days 1, 8 and 15. This has been studied in TNE patients and was well tolerated. There have been no studies examining lower doses of Ixazomib so dose reductions are not permitted out of concern for loss of efficacy. The same dose is used irrespective of frailty.

For peer review only - http://bmjopen.bmj.com/site/about/guidelines.xhtml

request can be made after cycle 4 for the start of cycle 6, but this requires at least a partial response (PR). This criteria applies for all participants irrespective of changes in frailty at cycles 3 and 5.

Intervention Adherence

Throughout the trial lenalidomide and ixazomib will be taken orally and swallowed whole at the same time on the scheduled days. Dexamethasone will be administrated in accordance with the relevant Summary of Product Characteristics (SPC). To monitor treatment adherence, participants will complete a daily medication diary which will be reviewed at trial visits. Unused capsules will be returned to pharmacy.

Dose Modification and Discontinuation

Both R1 and R2 treatment cycles will be 28 days in length. Response will be assessed at the end of each cycle according to the IMWG 2016 Uniform Response Criteria [26 27]. In the absence of progression or treatment intolerance, participants will receive a maximum of 12 cycles of induction therapy and continuous maintenance therapy. Those who receive 12 cycles of induction therapy will be assessed for R2 eligibility, with non-eligible participants being treated off trial.

Toxicity and hence treatment intolerance will be assessed throughout each treatment cycle, according to the National Cancer Institute (NCI) common terminology criteria for AE (CTCAE) Version 5.

For a new cycle of treatment to begin (induction and maintenance), the participant must meet the haematological and biochemical criteria (at day 1 or ≤3 days prior) outlined in the eligibility criteria (R1: Table 2, R2: Table 3). Non-haematological toxicities (except for alopecia) must have resolved to less than or equal to Grade 1 or to the participants baseline condition in order for treatment to resume. If the participant does not meet this criteria their dose will be delayed for one week before they are re-assessed. This will continue for a maximum of three weeks before treatment discontinuation or eight weeks at the discretion of the Chief Investigator.

In the event that a dose is reduced due to toxicity, as per the lenalidomide SPC the dose may be reintroduced to the next higher dose level upon improvement. Ixazomib, once reduced cannot be re-escalated.

Concomitant medication

Concomitant medication, disease and other malignancies will be recorded at eligibility.

All participants may receive additional care during the treatment period as deemed appropriate by the treating clinician. Local support care protocols, including anti-emetic schedules, tumor lysis syndrome prevention, VTE (Venous thromboembolism) prophylaxis and prophylactic antimicrobial therapy, will be followed for both randomisations. Permitted and excluded concomitant medications and procedures can be found in the supplementary material.

Outcomes

Primary outcome

For R1 the primary outcome is early treatment cessation (defined at a binary endpoint) for reactive versus adaptive dosing in participants defined to be "unfit" or "frail" at baseline. Participants will be defined to have experienced an event if they die, progress, or are withdrawn from treatment (by the treating clinician) or withdraw consent for treatment, within 60 days of R1.

For peer review only - http://bmjopen.bmj.com/site/about/guidelines.xhtml

For R2 the primary outcome is PFS for R+Placebo versus R+I, and is defined as the time from R2 to the time of first documented disease progression or death from any cause. Individuals lost to follow up or progression-free at time of analysis will be censored at their last known alive and progression-free date.

Secondary outcomes

The secondary outcomes of this trial are to assess PFS for reactive versus adaptive dosing, time to progression, time to 2nd PFS event (PFS2), overall survival (OS), survival after progression, deaths within 12 months of R1, overall response rate (ORR), attainment of ≥VGPR, attainment of MRD negativity, duration of response, time to improved response, time to next treatment, treatment compliance and total amount of therapy delivered, toxicity and safety including the incidence of Second Primary Malignancies (SPMs), Quality of Life (QoL), cost-effectiveness of reactive versus adaptive dosing of IRD and cost-effectiveness of R+I versus R.

Exploratory outcomes

Exploratory outcomes are to prospectively validate the UK-MRA Myeloma Risk Profile, to assess the usefulness of the Karnofsky performance status (PS), and consider the association of molecular subgroups with response, PFS and OS.

Participants Timelines

The full trial schema can be seen in Figure 3. The schedule of assessments at each time point are presented in Figure 4.

Trial Entry

Participants will enter the trial at one of two points in their patient pathway, this will either at bone marrow registration or main trial registration.

Trial consent

Participants who enter the trial at bone marrow registration will provide consent to having bone marrow and blood samples taken and sent to central laboratories for analysis. If the participant is diagnosed with a plasma cell dyscrasia, other than myeloma, or they have myeloma, but decide not to take part in the FiTNEss trial, they will also have the option of consenting to their samples being used in future research.

All participants, will provide written informed consent for the trial prior to trial registration. Optional consent for QoL resources and the use of samples for future research will also be obtained.

Trial Registration

Following trial consent participants will be registered onto the main trial and assessed for eligibility. Consenting patients will complete the baseline QoL and Healthcare resource use questionnaires. Trial samples for blood and urine will be taken for all participants and bone marrow samples will be taken for those who did not enter the trial through bone marrow registration.

Trial Treatment

Following trial registration participants will be randomised into R1 and treated as described in the intervention schedule on a monthly basis. In absence of disease progression or intolerance, those participants with at least an MR following 12 cycles of maintenance and whom meet all of the R2 eligibility criteria will proceed to R2 maintenance treatment.

For peer review only - http://bmjopen.bmj.com/site/about/guidelines.xhtml

Participants will be followed up monthly (at each cycle) whilst receiving maintenance treatment, until death or until the final analysis of the trial (whichever happens sooner).

Trial Follow-up

Participants who discontinue treatment during induction and before the point of R2 will be followed up to the point of assessment for eligibility for R2, unless they withdraw consent for this. Thereafter, participants will continue to be followed-up for data pertaining to safety, progression (including second progression), and survival. Frailty scores will be completed for all participants at 2, 4, 6 and 12 months post-R1, irrespective of whether they have discontinued treatment. Similarly, QoL and Healthcare resources use questionnaires (if the participant has consented to these) will continue to be completed at 2, 6 and 12 months post-R1.

If treatment has been stopped without progression following R2, e.g. due to toxicity, then participants will be followed up two monthly until disease progression. Follow-up will include local investigations, central investigations, frailty index at 6 and 12 months post-R2 and QoL and Healthcare resource use at 6 and 12 months post-R2 (if consented).

Sample Size

In total 740 participants will be enrolled into the trial at R1 to ensure that at least 478 participants remain on trial and are randomised to R2. It is assumed that 65% of those randomised at R1 will be progression-free and, therefore, eligible for R2, hence 740 participants are required to be enrolled.

Based on data from the Myeloma XI non-intensive pathway we hypothesise the frail and unfit patients in R1 will be similar to the older patients in Myeloma XI (>75 years) who have an early treatment cessation rate (within 60 days of R1) of 20%. This hypothesis is based on the expectation that the frailty score is heavily driven by patient age. Younger patients (≤75 years) in the Myeloma XI non-intensive pathway have an early treatment cessation rate of 9% and it is our hypothesis that our frailty-based dosing schedule has the potential to reduce the rate amongst the unfit and frail patients to the proportion observed in fit patients [28 29] .

To demonstrate a decrease of 11 percent points in the proportion of early treatment cessation from 20% in the standard dosing schedule arm to 9% in the frailty score-adjusted dose arm amongst those patients scored at baseline to be unfit or frail would require the recruitment of 324 patients with an allocation ratio of 1:1. These calculations are based on a Pearson's chi-squared test without continuity correction, assume a 2-sided 5% level of significance, 80% power, and allow for a 1% dropout prior to 60 days post-randomisation. Given that we anticipate that 45% of patients will be scored as unfit or frail, by assuming this trial will have a similar underlying population as in Myeloma XI non-intensive pathway and the age distributions in the IMWG report proposing the frailty score [14], we would anticipate that we will require 720 patients to enter the trial at R1 to have sufficient unfit and frail patients available to answer this question.

For R2, in the non-intensive pathway of Myeloma XI, the median PFS for patients on R following CRDa induction was approximately 33 months [19], where approximately 65% of individuals were progression-free 12 months post-randomisation. As R2 is approximately 12 months following R1 in FiTNEss we assume that the median PFS for patients receiving R maintenance therapy will be 21 months from R2. Tourmaline-MM1 [12] demonstrated a hazard ratio for PFS of 0.74 when comparing IRD and RD in patients with relapsed and refractory multiple myeloma. Thus a similar hazard ratio would be the minimum clinically relevant difference for our comparison in patients with newly diagnosed multiple myeloma. Assuming a median PFS of 29 months for those in the R+I maintenance group in addition to the assumption of 21 months in the R group equates to a hazard ratio of 0.72.

For peer review only - http://bmjopen.bmj.com/site/about/guidelines.xhtml

The above assumptions require the recruitment of 478 participants over a 30 month recruitment period with a further 24 months of follow-up. These calculations also assume a 2-sided 5% significance level, 80% power and allow for a 3% dropout rate prior to a PFS event being experienced. Note that 80% power is attained when 302 events have been observed. A total of 740 participants should be allocated to R1 to ensure that 478 participants are available at the second randomisation.

Overall survival is considered to be a key secondary endpoint for R2, 180 events with a minimum of 2 years follow up for all participants are required for 80% power.

Recruitment

It is planned that 740 participants will be recruited over a 30 month recruitment period from 87 UK centres. Once all centres are open, the recruitment target is 30 participants a month. The trial opened to recruitment on the 4th August 2020. As of May 2021 85 sites are open to recruitment and 137 participants have been randomised to the first stage of the trial.

In order to ensure the trial will meet the target sample size within the recruitment period, site set-up was prioritised whilst the trial was preparing to open to recruitment. In addition, the trial team are actively engaging with Principal Investigators at sites who support the trial to ensure that those sites open quickly and are maintaining regular communication with open sites to ensure that they continue to recruit to the trial. Finally, whilst the trial was originally delayed due to the COVID-19 pandemic, efforts were made to ensure that it opened as soon as possible when research restarted. One factor being the positive risk to benefit ratio of the number of hospital visits required for the trial interventions as compared to standard of care.

Assignment of treatment allocations

Each of the registration and randomisation procedures will be conducted centrally using the Leeds CTRU (Clinical Trial Research Unit) automated 24 hour web based and telephone system.

Registrations

If a patient is suspected to have myeloma they will enter the trial at the time of routine diagnostic tests, through bone marrow registration. Once diagnosis is confirmed locally and the research team consider the patient potentially eligible for the trial, patients will be provided with full verbal explanation of the trial and the full participant information sheet and informed consent documents to consider. Once the participant has provided informed consent, they will be registered onto the trial.

Other participants who have myeloma confirmed prior to entering trial, will enter at trial registration.

Randomisations

Following trial registration patients will be assessed for R1 eligibility. Eligible participants will be randomised on a 1:1 basis into R1, using the stratification factors; centre, IMWG frailty category, Beta-2 microglobulin concentration (<3.5, 3.5-<5.5, $\geq$5.5 mg/L), Haemaglobulin concentration (<100, $\geq 100g/L$), serum crestinine concentration (<175, $\geq$ 175 µmol/L), corrected serum calcium concentration (<2.75, $\geq$ 2.75mmol/L), platelets (<150, $\geq$ 150 x $10^9/L$).

Following R1, and as soon as the end of induction response is known, R2 eligible participants will be randomised on a 1:1 basis into R2, using the stratification factors; centre, allocated induction arm (reactive, adaptive); final response to induction treatment (<VGPR, $\geq$ VGPR).

For peer review only - http://bmjopen.bmj.com/site/about/guidelines.xhtml

For both R1 and R2, a computer generated minimization program that incorporates a random element will be used to ensure treatment groups are well balanced for the specified stratification factors.

Blinding Methods

For R2 treatment allocation will be concealed from participants, treatment provider and the trial team. The placebo and ixazomib capsules will be identical in colour, size, packaging and labelling.

To maintain the overall integrity of the trial design, unblinding will only be permitted in exceptional circumstances; for example valid medical or safety reasons where assuming that the patient is receiving active treatment and/ or stopping the blinded medication is insufficient.

Unblinding will be conducted automatically using an online system accessed by an authorised member of the site research team.

If unblinding is performed at any stage during the trial, decisions around further trial treatment will be the responsibility of the principal investigator or delegate. In either case unblinded participants will be followed up as per the protocol.

At the completion of the trial and after final analysis, participants will be given the opportunity to be informed of their allocation by their research site.

Data Collection

Clinical data will be collected both electronically and on paper by staff at research site completing CRFs provided by CTRU. QoL and Healthcare Resource Use questionnaires will be completed on paper CRFs by the participant. All paper CRFs will be sent to CTRU, by the research site, usually via standard post and entered onto an electronic database. This data along with the data entered electronically by staff at each research sites will be validated and monitored for completeness and quality by the CTRU.

Missing data will be chased until it is received, confirmed as not available or the trial is at analysis. Missing QoL data items will not be chased from participants, although missing questionnaires may be chased from sites.

Data management

Validation checks will be incorporated into the trial database to verify the data, and discrepancy reports will be generated for resolution by the trial site. Priority validations will be incorporated to ensure that any discrepancies related to participant rights, or the safety of participants, are expedited to sites for resolution. The CTRU/Sponsor will reserve the right to intermittently conduct source data verification exercises on a sample of participants, which will be carried out by staff from the CTRU/Sponsor. Source data verification will involve direct access to participant notes at the participating hospital sites and the ongoing central collection of copies of consent forms and other relevant investigation reports.

Statistical methods

Statistical analysis is the responsibility of the CTRU statisticians, with the exception of the analysis for cost-effectiveness of delivery of IRD and R/R+I, which will be undertaken by Health Economists at the University of Leeds. A full statistical analysis plan (SAP) and Health Economics Analysis Plan (HEAP) will be written and approved before any analysis is undertaken.

All analyses will be conducted on the intention-to-treat (ITT) population, where participants will be included according to their randomisation allocation regardless of eligibility, whether they

For peer review only - http://bmjopen.bmj.com/site/about/guidelines.xhtml

prematurely discontinued treatment, or did not comply with the regimen. A per-protocol (PP) analysis, where participants will be included if they received their allocated intervention according to the protocol, will be considered for the primary endpoints if there are a considerable number of major protocol violators. The safety population will consist of all participants who received at least one dose of the trial treatment and participants will be summarised as per their treatment received rather than their allocation.

An overall two-sided 5% significance level will be used for all efficacy endpoint comparisons. For the primary endpoints, this will be adjusted to account for the formal interim analyses.

Primary Endpoint Analysis

Randomisation 1

For R1, the number and proportion of participants, categorised as unfit or frail at baseline, experiencing an early treatment cessation event will be summarised by randomisation allocation and exact 95% confidence intervals will be calculated.

A logistic regression model will regress early treatment cessation on randomisation allocation (reactive / adaptive dosing) adjusting for the stratification factors of the trial. A statistically significant induction treatment effect will be suggested if the p-value for the resulting odds ratio is <0.047. Parameter estimates, odds ratios and corresponding 95% confidence intervals, degrees of freedom, test statistics and p-values will be presented for each variable in the model. Residuals and predicted values produced from the models will be examined to assess the assumptions of the statistical models.

Randomisation 2

For R2, PFS between the two maintenance therapies (R+Placebo / R+I) will be compared using a Cox regression model adjusting for the stratification factors of the trial. A statistically significant maintenance treatment effect will be suggested if the p-value for the hazard ratio corresponding to randomisation allocation is <0.047. Parameter estimates, hazard ratios and corresponding 95% confidence intervals, degrees of freedom, test statistics and p-values will be presented for each variable in the model.

The proportional hazards assumptions will be assessed by plotting the hazards over time (i.e. the log cumulative hazard plot) for each treatment arm and using appropriate statistical tests. If evidence is found to support the violation of the proportional hazards assumption then alternative appropriate analysis methods will be investigated.

No imputation strategy is planned for the primary endpoints.

Secondary Endpoint Analysis

Secondary endpoint analysis of OS and other time-to-event endpoints will be analysed using similar methods to those described for PFS.

MRD negativity and other binary endpoints will be analysed using similar methods to those described for the early treatment cessation primary endpoint. The number and proportion of participants in each response category (sCR, CR, VGPR, etc.) will be summarised by allocated treatment and exact

For peer review only - http://bmjopen.bmj.com/site/about/guidelines.xhtml

95% confidence intervals will be calculated. The difference in proportions for each response category will be presented with corresponding 95% confidence intervals.

The domains of the QoL questionnaires will be summarised using mean scores adjusted for baseline and 95% CIs at each assessment time-point. Similar summaries will be produced for quality-adjusted life years (QALYs) derived using the EQ-5D-3L questionnaire.

Exploratory and subgroup analyses

An overview of the planned exploratory and subgroup analysis can be found in the supplementary material. These include genetic and molecular analysis of patient samples conducted by the respective central laboratories.

Health economics

A full HEAP will be written and approved before any analysis is undertaken.

Economic evaluations will be conducted at R1 and R2, using within-trial and decision-model based analyses. The analysis will be guided by the NICE reference case, applying the cost-utility framework from the perspective of the health and social care provider over a life-time horizon. Base case QALYs will be based on EQ-5D-3L responses, and costs on patient completed resource use forms and hospital records. Results will be presented in terms of incremental cost-effectiveness ratios, cost-effectiveness acceptability frontiers and net benefit.

Trial Oversight

The trial management group (TMG), comprises of the Chief and Co-Chief Investigators, CTRU team and co-investigators and are responsible for the clinical set up, ongoing management, promotion of the trial and for the interpretation of the results. The trial steering committee (TSC), consisting of independent clinicians and statisticians, along with a patient representative, will provide overall supervision on the trial, including trial progress, adherence to protocol, participant safety and consideration of new information.

Data monitoring

An independent data monitoring and ethics committee (DMEC) will review the safety and ethics of the trial by reviewing unblinded interim data prepared by the CTRU in strict confidence at approximately yearly intervals. Unblinded safety updates are also prepared at 6 monthly intervals. After each annual review of safety data, the DMEC will make their recommendations to the TSC about the continuation of the trial who will make their recommendations known to the TMG.

Interim Analyses

Two formal interim analyses will be undertaken for early efficacy, one for each of the randomisations. The first will occur when 50% of required participants (370 participants) have reached 60 days post-R1. The second will occur when 50% of required PFS events have been observed (151 events) following R2. In order to maintain an overall two-sided 5% significance level for the primary endpoint analysis the O'Brien and Fleming alpha spending function [30] will be used. This results in a 0.05% significance level for the interim analysis. The analysis itself will reflect that detailed in the statistical analysis section. For the second interim analysis, only the DMEC, safety statistician and supervising statistician will see the unblinded results, as is standard procedure for double blind trials.

For peer review only - http://bmjopen.bmj.com/site/about/guidelines.xhtml

No other formal analysis of the trial is planned before the participants have attained the primary endpoints.

The DMEC, in the light of the interim data, and any advice or evidence they wish to request, will advise the TSC if there is proof beyond reasonable doubt that one treatment is better and recommend appropriate changes to the trial protocol.

Harms

Adverse Events

Adverse Events (AEs) are any untoward medical occurrence in a patient or clinical trial subject administrated a medicinal product and which does not necessarily have a causal relationship with this treatment. AEs can be defined as any unfavourable and unintended sign (including an abnormal laboratory finding, for example), symptom or disease. Due to the nature of myeloma and its treatment, participants are likely to experience several AEs throughout the course of the disease.

All AEs, both related and unrelated to myeloma treatment will be collected on the relevant CRF from R1 until 60 days after the last dose of protocol treatment and will be evaluated and summarised in accordance with the NCI-CTCAE V5.

Serious Adverse Events

Serious Adverse Events (SAEs) are defined as any untoward medical occurrences or effects that at any dose result in death; or are life threatening (at the time of event); or require in patient hospitalisation or prolongation of existing hospitalisation; or result in persistent or significant disability of incapacity; or result in a congenital abnormality or birth defect; or jeopardise the participant or may require an intervention to prevent one of the above outcomes/consequences (other important medical event). SAEs will be reported from R1 until 60 days post the last dose of trial drug.

Serious Adverse Reactions (SARs) are SAEs that are deemed to be possibly related to any dose administrated of any trial treatment. Suspected unexpected serious adverse reactions (SUSARs) are SARs, of which the nature and severity is not consistent with the applicable reference safety information. SUSARs and SARs will be reported from the date of the first trial drug for the duration of the trial.

Presenting Safety Data

Safety analyses will summarise all SUSARs, SARs, SAEs, ARs, AEs and treatment-related mortality rates. Safety data will be presented by treatment group for the safety population in addition to suspected relationship to trial treatment.

Secondary Primary Malignancies

All new secondary primary malignancies (SPMs) or suspected malignancies will be recorded from R1 for the duration of the trial and will be summarised and reviewed by an appointed member of the TMG, who will determine whether trial treatment should continue.

Pregnancies

For peer review only - http://bmjopen.bmj.com/site/about/guidelines.xhtml

For peer review only

The Celgene approved pregnancy programme will be followed as per usual clinical practice. Pregnancies in participants on trial treatment will be prevented as effectively as possible. Pregnancies and suspected pregnancies in a female or male participant's partner occurring at any time until 90 days post cessation of trial treatment will be reported.

Auditing

The CTRU and the trial Sponsor have procedures in place to ensure that serious breaches of GCP or the trial protocol are identified and reported. A triggered monitoring plan will ensure that sites at risk are monitored accordingly.

Patient and public involvement

FiTNEss has been developed following extensive discussion within the UK myeloma community, including with the NCRI Myeloma Subgroup (the UK Myeloma Research Alliance (UK-MRA)) and the NCRI Haematological Oncology Group. Both groups include patient and public representatives who work with clinical members of the group. To develop studies which address key questions for induction and maintenance.The protocol was reviewed in depth by a patient representative in order to ensure that the interventions and proposed schedule of assessments would be acceptable to patients. In addition, the trial consent and participant information document was reviewed for clarity by the same patient representative. Furthermore, to ensure that the patient perspective is considered throughout the trial, a patient representative sits on the TSC.

Ethics and Dissemination

Ethics approval statement

Ethical approval has been obtained from the North East - Tyne & Wear South Research Ethics Committee (reference 19/NE/0125). In addition approval was granted by the appropriate local research and development department for each participating centre prior to opening to recruitment. Participants will be required to provide written informed consent before joining the trial.

Protocol Amendments

The trial opened to recruitment on 4th August 2020 using protocol version 2, dated 10th October 2019. An amendment to protocol version 3 is anticipated in June 2021, which will include the addition of the secondary endpoint Event-Free Survival, clarification on the requirements around when face-to-face assessments should be conducted, and adding in the recommendation of 5 mg once daily of lenalidomide for participants with severe renal impairment, as opposed to 15mg every other day.

Consent

The Principal Investigator retains overall responsibility for the informed consent of participants at their site and must ensure that any medically qualified person delegated responsibility to participate in the informed consent process is duly authorized, trained and competent to participate according to ethical approved protocol, principles of Good Clinical Practice (GCP) and Declaration of Helsinki. Written consent will be obtained and signed by a medically qualified member of the site research team. A record of the consent process for both bone marrow and blood sample consent and full trial consent, including the date of consent and all those present, will be kept in the participant's notes. At any stage, participants can withdraw consent without repercussion.

Confidentiality

For peer review only - http://bmjopen.bmj.com/site/about/guidelines.xhtml

All information collected during the course of the trial will be kept strictly confidential. Information will be held securely on paper at Leeds CTRU. In addition, the CTRU will hold electronic information on all trial participants. The CTRU will have access to the entire database for monitoring, coordination, and analysis purposes.

Access to data

The CTRU will control the final trial datasets, and any requests for data will be reviewed by the trial management group in the first instance. Only requests that have a methodologically sound proposal and whose proposed use of the data has been approved by the independent trial steering committee, based on the principles of a controlled access approach, and subject to existing contractual agreements with the funder, will be considered. Proposals should be directed to the corresponding author in the first instance; to gain access, data requestors will need to sign a data access agreement.

Ancillary and post-trial care

Participants who stop trial treatment due to progression or any point prior to the end of trial will be treated off-trial at the discretion of their treating clinician. Following disease progression, participants will be followed up annually until death, or until the end of the trial for post-progression endpoints.

Dissemination policy

Authorship of clinical and translational outputs will be in keeping with the UK-MRA Publication Policy and due acknowledgement to participants, local investigators, funders and NCRI Haematological-Oncology Group support made. The success of the trial depends upon the collaboration of all trial members. For this reason, credit for the main results will be given to all those who have collaborated in the trial, through authorship and contributorship. Uniform requirements for authorship for manuscripts submitted to medical journals will guide authorship decisions alongside the guidance of the UK-MRA.

To maintain the scientific integrity of the trial, data will not be released prior to the end of the trial or a primary endpoint being reached, either for trial publication or oral presentation purposes, without the permission of the Trial Steering Committee and the (Co-)Chief Investigators. In addition, individual collaborators must not publish data concerning their participants that is directly relevant to the questions posed in the trial until the main results of the trial have been published and following written consent from the Sponsor.

Appendices

Informed consent material

The consent forms which are to be completed by the participant at Bone Marrow Registration and/or Trial Registration are included in supplementary material.

Biological specimens

The collection of central samples for laboratory analysis is summarised in Figure 4. The analysis to be conducted for trial purposes is stated in the supplementary material. Additional analysis may be carried out by each central laboratory provided the appropriate consent for sample use in future research has been provided by the participant at trial entry.

<u>**Author Contributions**</u>

For peer review only - http://bmjopen.bmj.com/site/about/guidelines.xhtml

The FiTNEss trial was conceived by GC and GJ and designed by them in collaboration with CPaw, DAC, AH, JB, SB, MK, RO, MJ, BK, MD. All authors inputted into the development of the protocol and patient information sheet. The first draft of the manuscript was written by ABC and KLR. All authors reviewed and approved the final manuscript.

Funding

This trial is funded by Cancer Research UK (C37712/A21282) with further unrestricted funding from Millennium: The Takeda Oncology Company and Celgene: A Bristol Myers Squibb Company. This work was also supported by Core Clinical Trials Unit Infrastructure from Cancer Research UK (C7852/A25447). The funders had no role in the design, collection, analysis or collection of data; in writing the manuscript; or in the decision to submit the manuscript for publication.

The trial Sponsor is responsible for the overall conduct of the trial as defined by Directive 2001/20/EC is University of Leeds, UoL/LTHT Joint Sponsor QA office (CTIMPs); Research & Innovation Centre/Faculty of Medicine & Health; Leeds Teaching Hospitals NHS Trust/University of Leeds; St James University Hospital; Leeds LS9 7TF.

Competing Interest Statement

ABC, KLR, DAC, AH, CO, RH, PB report grants and non-financial support from BMS/Celgene, grants and non-financial support from Merck Sharpe & Dohme, grants and non-financial support from Amgen, grants and non-financial support from Takeda, during the conduct of the trial. DAC also reports travel support from Celgene Corporation.

Cpaw reports receiving honoraria and/or travel support from Amgen, BMS/Celgene, Janssen, Sanofi, Takeda.

MK consultancy: AbbVie, Amgen, BMS/Celgene, GSK, Janssen, Karyopharm, Seattle Genetics, Takeda; honoraria: BMS/Celgene, Janssen, Takeda; Research funding: BMS/Celgene; Travel/educational support: BMS/Celgene, Janssen, Takeda.

Stella Bowcock reports receiving research funding from Takeda.

KB – Advisory Boards Janssen: BMS/Celgene, Takeda, Novartis. Speaker Honoraria: Janssen, BMS/Celgene, Sanofi, Takeda. Support to attend educational meetings: Janssen, BMS/Celgene, Takeda, GSK.

GJ reports research funding from Takeda, Onyx, MSD & BMS/Celgene with consultancy from Janssen, Takeda, Sanofi, Oncopeptides, Karyopharm, Pfizer, Roche & BMS/Celgene.

GC reports research funding from Janssen, Takeda, Amgen & BMS/Celgene with consultancy from Janssen, Takeda, Sanofi, Oncopeptides, Karyopharm, Pfizer, Roche & BMS/Celgene.

MD reports Stock held in Abingdon Health Ltd

JB, RDT, NR, JJ, CPar, HG, DM, BD, RO, MJ and BK have no declared competing interests.

For peer review only

References

1. CANCER RESEARCH UK. Myeloma Incidence Statistics [online]: CANCER RESEARCH UK; 2015 [Available from: http://www.cancerresearchuk.org/health-professional/cancer-statistics/statistics-by-cancer-type/myeloma/incidence accessed June.
2. Morgan GJ, Davies FE, Gregory WM, et al. Long-term Follow-up of MRC Myeloma IX Trial: Survival Outcomes with Bisphosphonate and Thalidomide Treatment. *Clinical cancer research* 2013;19(21):6030-38. doi: 10.1158/1078-0432.CCR-12-3211
3. Pawlyn C, Cairns D, Kaiser M, et al. The relative importance of factors predicting outcome for myeloma patients at different ages: results from 3894 patients in the Myeloma XI trial. 2020
4. Jackson GH, Davies FE, Pawlyn C, et al. Response-adapted intensification with cyclophosphamide, bortezomib, and dexamethasone versus no intensification in patients with newly diagnosed multiple myeloma (Myeloma XI): a multicentre, open-label, randomised, phase 3 trial. *The Lancet Haematology* 2019;6(12):e616-e29. doi: 10.1016/S2352-3026(19)30167-X
5. Pawlyn C, Davies FE, Cairns DA, et al. Quadruplet Vs Sequential Triplet Induction Therapy Approaches to Maximise Response for Newly Diagnosed, Transplant Eligible, Myeloma Patients. *Blood* 2015;126(189)
6. Jackson GH, Pawlyn C, Cairns DA, et al. Carfilzomib, lenalidomide, dexamethasone, and cyclophosphamide (KRdc) as induction therapy for transplant-eligible, newly diagnosed multiple myeloma patients (Myeloma XI+): Interim analysis of an open-label randomised controlled trial. *PLoS medicine* 2021;18(1):e1003454-e54. doi: 10.1371/journal.pmed.1003454
7. Richardson PG, Baz R, Wang M, et al. Phase 1 study of twice-weekly ixazomib, an oral proteasome inhibitor, in relapsed/refractory multiple myeloma patients. *Blood* 2014;124(7):1038-46. doi: 10.1182/blood-2014-01-548826
8. Offidani M, Corvatta L, Caraffa P, et al. An evidence-based review of ixazomib citrate and its potential in the treatment of newly diagnosed multiple myeloma. *OncoTargets and therapy* 2014;7:1793-800. doi: 10.2147/OTT.S49187
9. Assouline SE, Chang J, Cheson BD, et al. Phase 1 dose-escalation study of IV ixazomib, an investigational proteasome inhibitor, in patients with relapsed/refractory lymphoma. *Blood cancer journal (New York)* 2014;4(10):e251-e51. doi: 10.1038/bcj.2014.71
10. Gupta N, Zhao Y, Hui AM, et al. Switching from body surface area-based to fixed dosing for the investigational proteasome inhibitor ixazomib: a population pharmacokinetic analysis. *British journal of clinical pharmacology* 2015;79(5):789-800. doi: 10.1111/bcp.12542
11. Kumar SK, Berdeja JG, Niesvizky R, et al. Safety and tolerability of ixazomib, an oral proteasome inhibitor, in combination with lenalidomide and dexamethasone in patients with previously untreated multiple myeloma: an open-label phase 1/2 study. *The Lancet Oncology* 2014;15(13):1503-12.
12. Moreau P, Masszi T, Grzasko N, et al. Ixazomib, an investigational oral proteasome inhibitor (PI), in combination with lenalidomide and dexamethasone (IRd), significantly extends progression-free survival (PFS) for patients (Pts) with relapsed and/or refractory multiple myeloma (RRMM): the phase 3 Tourmaline-MM1 study (NCT01564537): Am Soc Hematology, 2015.
13. Facon T, Venner CP, Bahlis NJ, et al. MM-347: Ixazomib Plus Lenalidomide-Dexamethasone (IRd) vs. Placebo-Rd for Newly Diagnosed Multiple Myeloma (NDMM) Patients Not Eligible for Autologous Stem Cell Transplant: The Double-Blind, Placebo-Controlled, Phase 3 TOURMALINE-MM2 Trial. *Clinical lymphoma, myeloma and leukemia* 2020;20:S307-S08. doi: 10.1016/S2152-2650(20)30955-1
14. Palumbo A, Bringhen S, Mateos M-V, et al. Geriatric assessment predicts survival and toxicities in elderly myeloma patients: an International Myeloma Working Group report. *Blood* 2015;125(13):2068-74.

For peer review only - http://bmjopen.bmj.com/site/about/guidelines.xhtml

15. Katz S, Ford AB, Moskowitz RW, et al. Studies of illness in the aged: The index of adl: a standardized measure of biological and psychosocial function. *JAMA* 1963;185(12):914-19. doi: 10.1001/jama.1963.03060120024016
16. Lawton MP, Brody EM. Assessment of older people: self-maintaining and instrumental activities of daily living. *The gerontologist* 1969;9(3_Part_1):179-86.
17. Charlson ME, Pompei P, Ales KL, et al. A new method of classifying prognostic comorbidity in longitudinal studies: development and validation. *Journal of chronic diseases* 1987;40(5):373-83.
18. Delforge M, Minuk L, Eisenmann J-C, et al. Health-related quality-of-life in patients with newly diagnosed multiple myeloma in the FIRST trial: lenalidomide plus low-dose dexamethasone versus melphalan, prednisone, thalidomide. *Haematologica* 2015;100(6):826-33. doi: 10.3324/haematol.2014.120121
19. Jackson GH, Davies FE, Pawlyn C, et al. Lenalidomide Is a Highly Effective Maintenance Therapy in Myeloma Patients of All Ages; Results of the Phase III Myeloma XI Study. *Blood* 2016;128(22):1143-43.
20. Jackson G, Davies FE, Pawlyn C, et al. Lenalidomide Maintenance Significantly Improves Outcomes Compared to Observation Irrespective of Cytogenetic Risk: Results of the Myeloma XI Trial. *Blood* 2017;130(Supplement 1):436-36. doi: 10.1182/blood.V130.Suppl_1.436.436 %J Blood
21. Kumar S, Berdeja JG, Niesvizky R, et al. Long-Term Ixazomib Maintenance Is Tolerable and Improves Depth of Response Following Ixazomib-Lenalidomide-Dexamethasone Induction in Patients (Pts) with Previously Untreated Multiple Myeloma (MM): Phase 2 Study Results. *Blood* 2014;124(21):82-82.
22. Dimopoulos MA, Spicka I, Quach H, et al. Ixazomib as Postinduction Maintenance for Patients With Newly Diagnosed Multiple Myeloma Not Undergoing Autologous Stem Cell Transplantation: The Phase III TOURMALINE-MM4 Trial. *Journal of clinical oncology* 2020;38(34):4030-41. doi: 10.1200/JCO.20.02060
23. Chan A-W, Tetzlaff JM, Altman DG, et al. SPIRIT 2013 Statement: Defining Standard Protocol Items for Clinical Trials. *Annals of internal medicine* 2013;158(3):200-07. doi: 10.7326/0003-4819-158-3-201302050-00583
24. Chan A-W, Tetzlaff JM, Gøtzsche PC, et al. SPIRIT 2013 explanation and elaboration: guidance for protocols of clinical trials. *BMJ (Online)* 2013;346(jan08 15):907-e7586. doi: 10.1136/bmj.e7586
25. The International Myeloma Working Group (IMWG). updated criteria for the diagnosis of multiple myeloma. *The Lancet 2014*;15 (12):538-48 (43).
26. Rajkumar SV, Harousseau JL, Durie B, et al. Consensus recommendations for the uniform reporting of clinical trials: report of the International Myeloma Workshop Consensus Panel 1. *Blood* 2011;117(18):4691-5. doi: 10.1182/blood-2010-10-299487
27. Kumar S, Paiva B, Anderson KC, et al. International Myeloma Working Group consensus criteria for response and minimal residual disease assessment in multiple myeloma. *The Lancet Oncology* 2016;17(8):e328-46. doi: 10.1016/S1470-2045(16)30206-6
28. Pawlyn C, Davies, F.E., Cairns, D., Striha, A., Best, P., Sigsworth, R., Jones, J.R., Kishore, B., Garg, M., Williams, C. and Karunanithi, K.,. Continuous Treatment with Lenalidomide Improves Outcomes in Newly Diagnosed Myeloma Patients Not Eligible for Autologous Stem Cell Transplant: Results of the Myeloma XI Trial. *ASH 2017* 2017
29. Jackson GH, Pawlyn C, Cairns DA, et al. Optimising the value of immunomodulatory drugs during induction and maintenance in transplant ineligible patients with newly diagnosed multiple myeloma: results from Myeloma XI, a multicentre, open-label, randomised, Phase III trial. *Br J Haematol* 2020 doi: 10.1111/bjh.16945 [published Online First: 2020/07/14]
30. O'Brien PC, Fleming TR. Multiple Testing Procedure for Clinical-Trials. *Biometrics* 1979;35(3):549-56. doi: Doi 10.2307/2530245

For peer review only - http://bmjopen.bmj.com/site/about/guidelines.xhtml

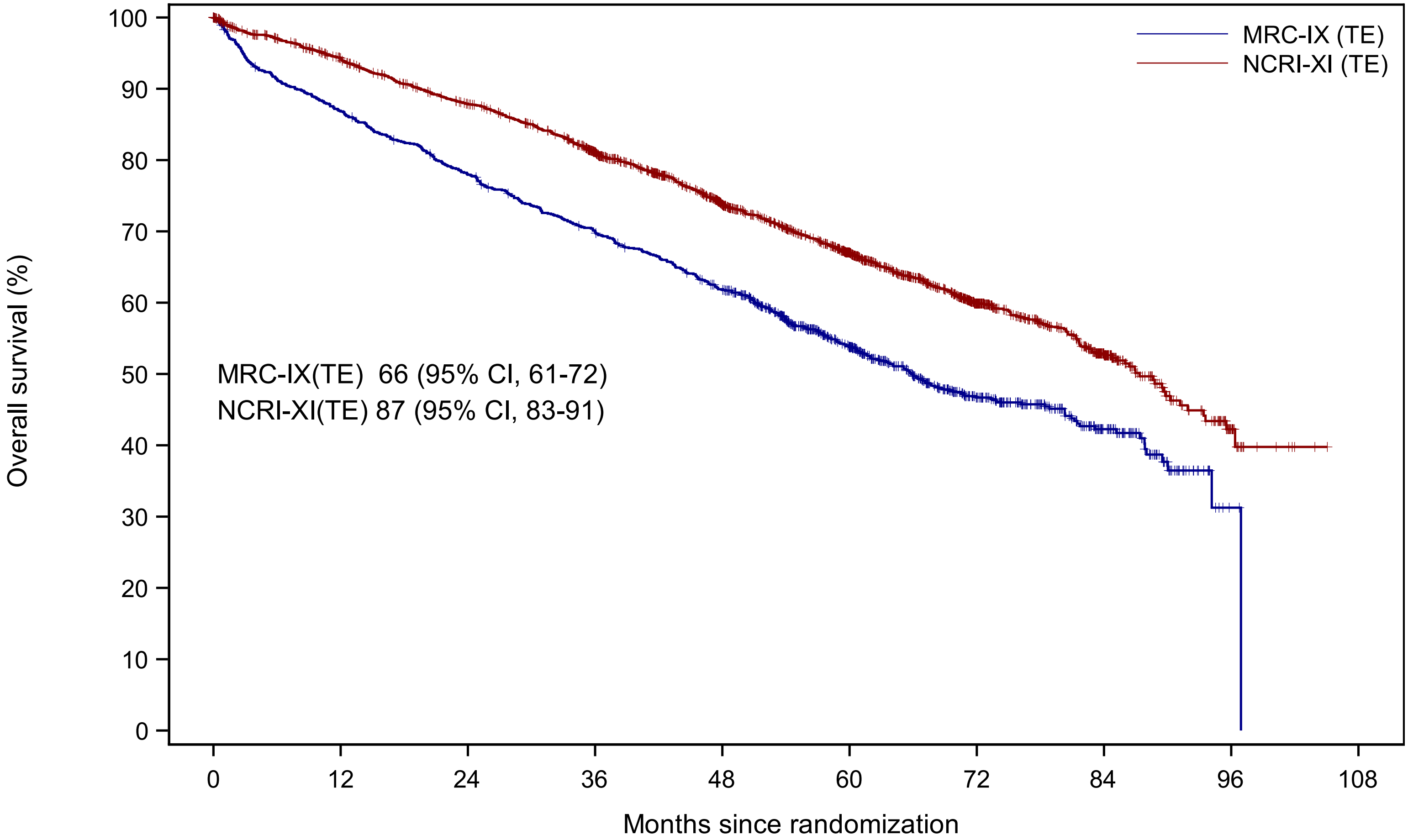

Number at risk (number censored)

MRC-IX (TE)	1111 (0)	961 (4)	860 (7)	768 (12)	670 (21)	436 (178)	224 (345)	89 (466)	2 (545)	0 (546)
NCRI-XI (TE)	2042 (7)	1848 (80)	1689 (114)	1478 (197)	1168 (383)	868 (584)	484 (891)	196 (1E3)	24 (1E3)	0 (1E3)

For peer review only - http://bmjopen.bmj.com/site/about/guidelines.xhtml

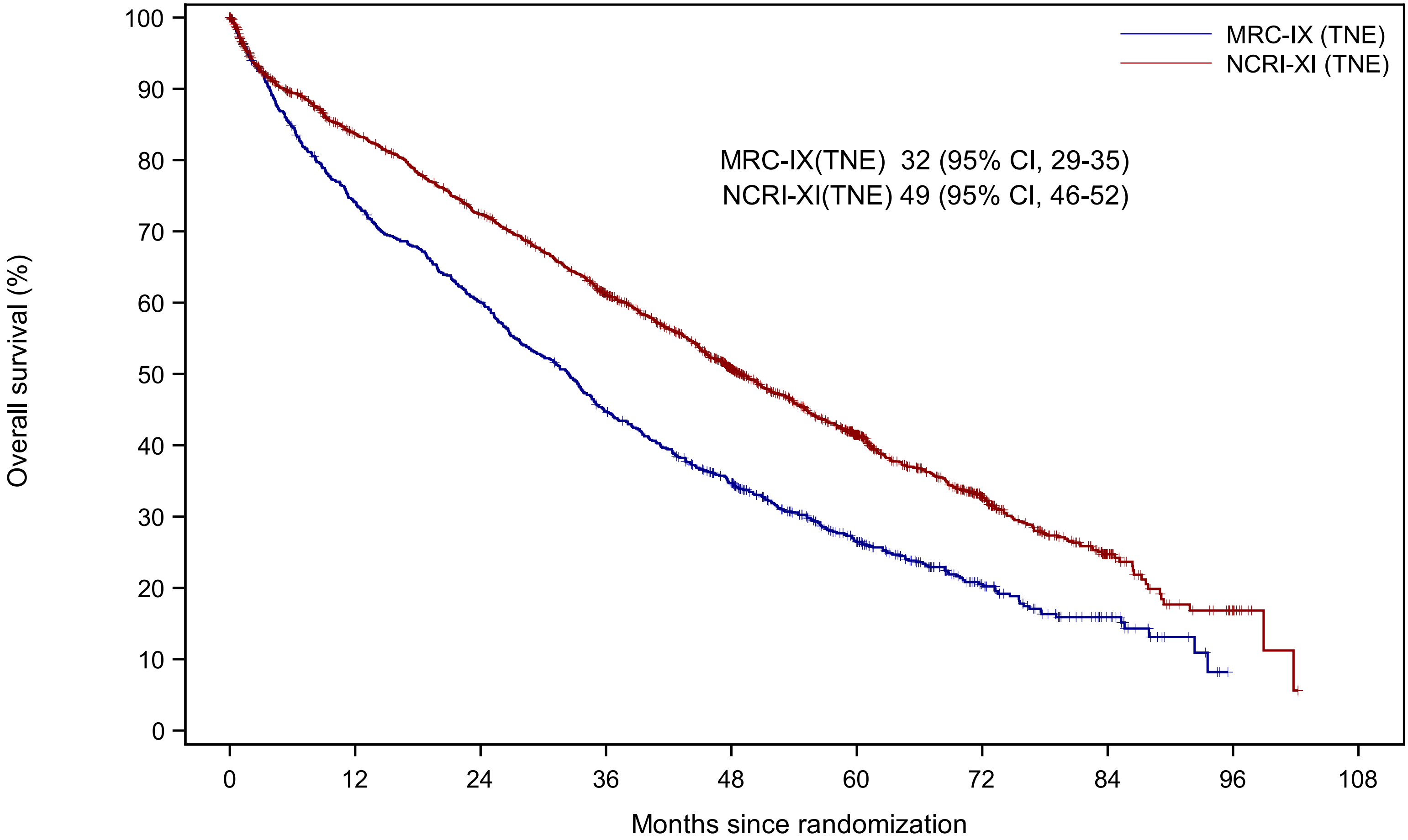

Number at risk (number censored)

	0	12	24	36	48	60	72	84	96	108
MRC-IX (TNE)	849 (1)	621 (10)	502 (11)	372 (14)	270 (34)	151 (99)	66 (158)	25 (186)	0 (206)	
NCRI-XI (TNE)	1852 (7)	1452 (108)	1244 (121)	1011 (163)	708 (304)	409 (489)	197 (631)	60 (731)	10 (769)	0 (777)

For peer review only - http://bmjopen.bmj.com/site/about/guidelines.xhtml

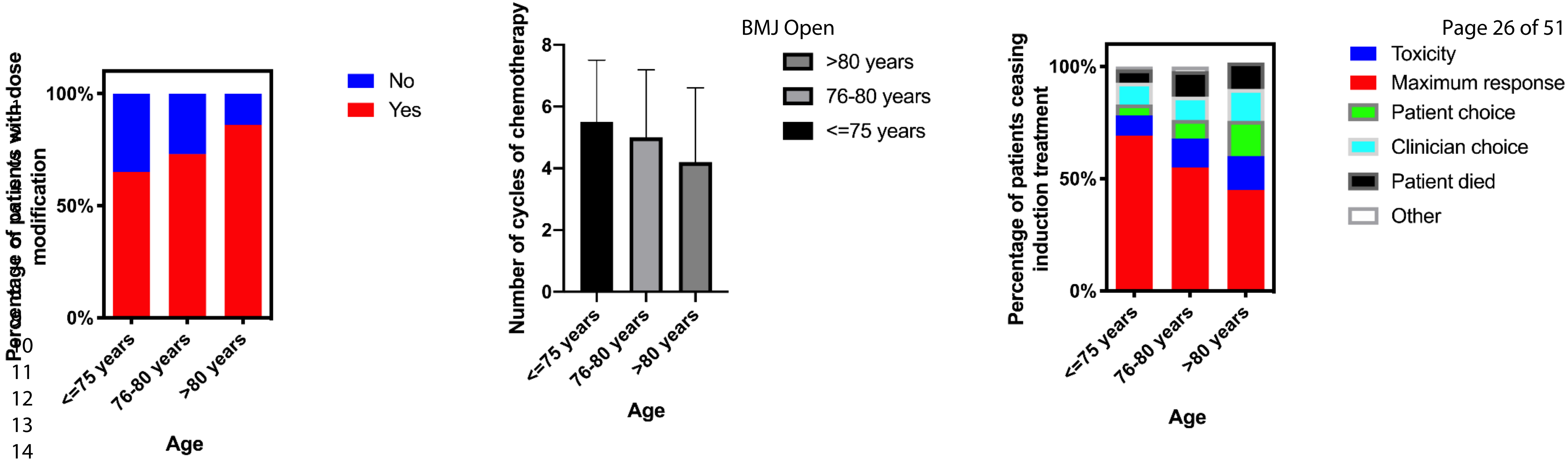
Percentage of patients with dose modification
100%
50%
0%
<=75 years
76-80 years
>80 years
Age
No
Yes
Number of cycles of chemotherapy
8
6
4
2
0
<=75 years
76-80 years
>80 years
Age
>80 years
76-80 years
<=75 years
Percentage of patients ceasing induction treatment
100%
50%
0%
<=75 years
76-80 years
>80 years
Age
Toxicity
Maximum response
Patient choice
Clinician choice
Patient died
Other

Bone marrow and blood sample consent and **registration** at the time of diagnostic bone marrow procedure, where possible*

*NB: If considering patient for inclusion in the trial, please obtain consent to send diagnostic bone marrow and a peripheral blood sample to trial central laboratories at the time of the procedure using the trial specific bone marrow and blood sample consent form .

Following confirmation of myeloma diagnosis:
Full **informed consent** and **trial registration**

Eligibility confirmation, frailty indexing (age, Charlson index, ADL and IADL), and (for consenting participants) QoL and Healthcare resource use questionnaires

Randomisation 1 (R1) (n = 740)
Standard dosing (reactive) *vs* Frailty score-adjusted (adaptive) (1:1 randomisation)

Standard dosing IRD
28-day cycle

	STANDARD DOSE	Days
I	4mg	1, 8, 15
R	25mg	1-21
D	40mg in participants ≤75yrs<br>20mg in participants >75 yrs	1, 8, 15, 22

Frailty score-adjusted dosing IRD
28-day cycle

	FIT	UNFIT	FRAIL	Days
I	4mg	4mg	4mg	1, 8, 15
R	25mg	15mg	10mg	1-21
D	40mg	20mg	10mg	1, 8, 15, 22

Response assessed after each cycle in line with IMWG Uniform Response Criteria

Frailty Score repeated after cycle 2, 4, 6 and 12*

QoL & HE questionnaires after cycles 2, 6 and 12* (for consenting participants)

*Or months from R1 if off trial prior to progression

In the absence of disease progression or intolerance, treat for 12 cycles, then assess end of induction

≥ Minimal response

Stable Disease or Disease

Treat off-trial
Annual follow-up

Randomisation 2 (R2) (n = 478)
R + placebo *vs* R + I maintenance (1:1 randomisation)

R + placebo maintenance
28-day cycle
R – 10 mg† D1 – 21
Placebo – 4 mg† D1, 8, 15
† or final doses administered at the end of induction treatment if lower

R + I maintenance
28-day cycle
R – 10 mg† D1 – 21
I – 4 mg† D1, 8, 15
† or final doses administered at the end of induction treatment if lower

Response assessed after each cycle in line with IMWG Uniform Response Criteria

Frailty Score repeated after maintenance cycles 6 and 12*

QoL & HE questionnaires after maintenance cycles 6 and 12* (for consenting participants)

*Or months from R2 if off trial prior to progression

Continue until disease progression or intolerance

Post-progression annual follow-up for survival

Figure 3: Flow Diagram of the trial

Figure 4: Schedule of assessments

Local Investigations							
	**Consent & trial registration**	**Pre-R1 (assessments for eligibility within 14 days prior to R1, unless otherwise stated)**	**Day 1 (or ≤3 days prior) of each induction treatment cycle**	**End of 12 cycles of IRD induction treatment**	**Pre-Randomisation 2 (R2) (assessments for eligibility within 14 days prior to R2, do not need repeating if end of IRD assessments are within this timeframe)**	**Day 1 (or ≤3 days prior) of each maintenance treatment cycle**	**Disease Progression**
Pre-trial Consent for Bone Marrow (at time of diagnostic bone marrow) and bone marrow registration	✓						
Full written informed consent and (main) trial registration	✓						
Medical history (including comorbidities, concomitant medications and previous malignancies)		✓					
Assessment of cardiac and thyroid function (as part of standard care)		✓	Monitor throughout treatment				
Physical examination (including height/weight, BP, performance status [f], vital signs [baseline only])		✓	✓	✓	✓	✓	✓
Pregnancy test (if female of child bearing potential, see Appendix 8)		✓	✓	✓	✓	✓[b]	
FBC, U&Es, calcium, creatinine, LFTs (including bilirubin, and AST and/or ALT), albumin, LDH, calculated creatinine clearance, Urinary protein:creatinine ratio* **only requested at baseline*		✓	✓[a]	✓	✓	✓	✓
CRP, β2M		✓	✓ After cycle 2, 4, and 6 [d]	✓		✓ After cycles 6 and 12 [c]	✓
Serum paraprotein, serum free light chains, serum total (class-specific) immunoglobulins and urinary light chain detection (quantification where available)		✓	✓ (To assess response to previous cycle, not applicable for C1)	✓	✓	✓ (To assess response to previous cycle)	✓
Bone marrow aspirate and (if available) trephine		✓ (Within 6 weeks prior to R1) [g]	If at any point a first occurrence of CR or sCR is suspected then bone marrow aspirate should be sent for local review as well as to HMDS, as detailed below. (s)CR cannot be confirmed without bone marrow.				✓
Serology of hepatitis B and C		✓					
Imaging		✓ (Within 3 months prior to randomisation)	Imaging of lytic and/or focal bone and extramedullary lesions if clinically indicated, in accordance with IMWG recommendations and local practice [e]				
IMWG Frailty Index (Charlson Comorbidity Score, IADL, ADL)		✓	✓ After cycle 2, 4, and 6 [d]	✓		✓ After cycles 6 and 12 [c]	
Quality of Life and Healthcare resource use questionnaires (EORTC QLQ-C30, QLQ-MY20, EQ-5D (3 Level)) **Completed by consenting participants in clinic*		✓ Before participant is informed of allocated dosing strategy	✓ After cycle 2 and 6 [d]	✓[d]		✓ After cycles 6 and 12 [c]	
Adverse Events			Monitor throughout study and report on relevant eCRFs (From randomisation until 60 days post last treatment dose)				
SAEs / SUSARs / SPMs			Monitor throughout study: all SUSARs & SAEs/SPMs must be reported to CTRU within 24 hours of the Site becoming aware of the event: refer to Section 14: (SAEs from randomisation until 60 days post last treatment dose & SPMs/SARs/SUSARs from first treatment dose until the end of trial)				

Central Analysis Investigations: Central Samples (all Participants – core consent)								
**Sample**	**Send to**	**Investigation**	**Bone marrow and blood sample consent samples only**	**Pre-randomisation 1 (pre-treatment) - Post Main Consent**	**During induction therapy**	**At the end of 12 cycles of IRD induction treatment (or at end of the final IRD cycle if sooner)**	**During maintenance therapy**	**Disease Progression**
20 mL whole clotted blood or 10 mL serum	Birmingham	To confirm disease response & progression through investigation of paraprotein, immunoglobulins, serum free light chain, urinary free light chain and β2M		✓	✓ (After 2 cycles of IRD)	✓	✓ (At 2 monthly intervals)	✓
10 mL random urine				✓	✓ (After 2 cycles of IRD)	✓	✓ (At 2 monthly intervals)	✓
2 mL bone marrow aspirate in EDTA	HMDS, Leeds	To determine MRD	✓(Within 8 weeks prior to R1)	✓[¥]	✓ (After 6 cycles of IRD)	✓	✓ (At 12 months post-maintenance randomisation (R2))	✓
					If at any point a first occurrence of CR or sCR is suspected then bone marrow aspirate should be sent to HMDS.			
3 mL bone marrow aspirate in EDTA	ICR, London	Cytogenetic/Molecular research (including MLPA / FISH)	✓(Within 8 weeks prior to R1)	✓*[¥]				✓
6 mL peripheral blood in EDTA			✓(Within 8 weeks prior to R1)	✓[¥]				✓
20 mL peripheral blood in EDTA	LIMR, Leeds	Frailty biomarker studies		✓	✓ (After cycle 2, 4 & 6)	✓		✓
10 mL clotted blood				✓	✓ (After cycle 2, 4 & 6)	✓		✓
2 mL bone marrow aspirate in EDTA						✓ **	✓ (At 12 months post-maintenance randomisation (R2))	✓

a. The FBC should be repeated mid-cycle 1 (Day 14 +/-3 days) or more frequently and during subsequent cycles if there is a concern about cytopenias.
b. Pregnancy test must also be performed at 4 weeks after the end of study treatment.
c. Or at 6 and 12 months post-R2 if treatment is stopped prior to this for reasons other than disease progression.
d. Or at 2, 6 and 12 months post-R1 if treatment is stopped prior to this for reasons other than disease progression.
e. The imaging at baseline/pre-registration is mandatory. Subsequent imaging will be as per local protocols/standard of care - imaging will only need to be repeated if extramedullary disease was detected at baseline, or in the event of new symptoms suggestive of new extramedullary disease, cord compression, new fracture, etc., or to investigate new hypercalcaemia. In the rare event of participants with extramedullary disease at baseline, imaging should be performed at the end of IRD induction to confirm the end of induction response, and at any other time the disease parameters suggest that the participant has achieved a complete response (if not already achieved at the end of induction) to confirm that the extramedullary disease has resolved.
f. Performance status should be recorded at the same timepoints as the frailty index (i.e. prior to R1, after cycles 2, 4, 6 and 12 of induction, and after cycles 6 and 12 of maintenance). Performance status should also be recorded at the time of disease progression. Or at 2, 6 and 12 months post-R1 if treatment is stopped prior to this for reasons other than disease progression.
g. If the participant did not consent to the pre-trial bone marrow registration part of Myeloma XIV, the bone marrow biopsy will need to be taken after full informed consent.
[¥] If not sent at bone marrow and blood sample consent.
* At pre-randomisation 1 a CD138 negative portion of the bone marrow aspirate will be sent to LIMR after CD138 positive selection at ICR.
** At the end of induction bone marrow aspirate will be sent to ICR after CD138 positive selection at LIMR.

Table 1: Inclusion and Exclusion Criteria for R1

**Inclusion Criteria:**
1. Newly diagnosed as having MM according to the updated IMWG diagnostic criteria 2014 (see Appendix 1 for criteria) requiring treatment.
2. Not eligible for stem cell transplant.
3. Aged at least 18 years.
4. Meet all of the following blood criteria within 14 days before R1:<br>Haematological:<br>a) Absolute neutrophil count (ANC) ≥ 1 x 109/L. Unless the participant has a known/suspected diagnosis of familial or racial neutropenia in which case an ANC ≥ 0.75 x 109/L is allowed. The use of growth factor support is permitted.<br>b) Platelet count ≥ 50 x 109/L, or, in the case of heavy bone marrow infiltration (≥ 50%) which in the opinion of the investigator is the cause of the thrombocytopenia and provided appropriate supportive measures and patient monitoring are in place, platelet count ≥ 30x 109/L is permitted. Please note: Platelet transfusions are not allowed ≤ 3 days prior to randomisation in order to meet these values.<br>c) Haemoglobin ≥ 80 g/L. The use of red blood cell transfusions is permitted.<br>Biochemical:<br>d) Total bilirubin ≤ 3 x upper limit of normal (ULN).<br>e) Alanine aminotransferase (ALT) and/or aspartate aminotransferase (AST) ⍰ 3 ⍰ ULN.
5. Meet the pregnancy prevention requirements:<br>Female participants who:<br>a) Are not of childbearing potential (Appendix 8), OR<br>b) If they are of childbearing potential, agree to practice 2 effective methods of contraception (Appendix 8), at the same time, from the time of signing the informed consent form until 90 days after the last dose of study drug, OR<br>c) Agree to practice true abstinence when this is in line with the preferred and usual lifestyle of the subject. (Periodic abstinence [e.g. calendar, ovulation, symptothermal, post-ovulation methods] and withdrawal are not acceptable methods of contraception.)<br>Male participants, even if surgically sterilised (i.e. status post-vasectomy), must agree to one of the following:<br>a) Agree to practice effective barrier contraception during the entire study treatment period and through 90 days after the last dose of study drug, OR<br>b) Agree to practice true abstinence when this is in line with the preferred and usual lifestyle of the subject. (Periodic abstinence (e.g. calendar, ovulation, symptothermal, post-ovulation methods] and withdrawal are not acceptable methods of contraception).<br>Contraception for female and male participants must be in accordance with (and participants must consent to) the Celgene-approved Pregnancy Prevention Programme.<br>If female and of childbearing potential (see Appendix 8), they must have a negative pregnancy test performed by a healthcare professional in accordance with the Celgene Pregnancy Prevention Programme.
6. Able to provide written informed consent.
**Exclusion Criteria:**
1. Smouldering MM, MGUS, solitary plasmacytoma of bone, or extramedullary plasmacytoma (without evidence of MM).
2. Received previous treatment for MM, with the exception of local radiotherapy to relieve bone pain or spinal cord compression, prior bisphosphonate treatment, or corticosteroids as long as the total dose does not exceed the equivalent of 160 mg dexamethasone.
3. Known resistance, intolerance or sensitivity to any component of the planned therapies.
4. Prior or concurrent invasive malignancies except the following:<br>- Adequately treated basal cell or squamous cell skin cancer;<br>- Incidental finding of low grade (Gleason 3+3 or less) prostate cancer requiring no intervention;<br>- Adequately treated carcinoma in situ of the breast or cervix no longer requiring medical or surgical intervention;<br>- Any cancer from which the subject has been disease-free for at least 3 years.
5. Pregnant, lactating or breastfeeding female participants.<br>6. Major surgery within 14 days before randomisation. This would include surgical intervention for relief of cord compression but does not include vertebroplasty or kyphoplasty.
7. Systemic treatment, within 14 days before the first dose of ixazomib with strong CYP3A inducers (e.g. rifampicin, rifabutin, carbamazepine, phenytoin, phenobarbital), or use of St. John's wort.
8. Any concomitant drug therapy which, in the opinion of the investigator, may lead to an unacceptable interaction with any of the agents ixazomib, lenalidomide, dexamethasone, and that cannot be safely stopped prior to trial entry. Full details of interactions can be found in the SPCs.
9. Known gastrointestinal (GI) disease or GI procedure that could interfere with the oral absorption or tolerance of trial treatment, including difficulty swallowing.
10. ≥ Grade 2 peripheral neuropathy.
11. Known HIV positive
12. Participant has current or prior hepatitis B surface antigen positive or hepatitis C antibody positive. Participants must have screening conducted within 14 days before R1.
13. Active systemic infection.
14. Any other medical or psychiatric condition which, in the opinion of the investigator, contraindicates the participant's participation in this study.
15. Receipt of live vaccination within 30 days prior to R1.

*MM – Multiple Myeloma, SPCs- Summary of product characteristics

For peer review only - http://bmjopen.bmj.com/site/about/guidelines.xhtml

Table 2: Inclusion and Exclusion Criteria for R2

**R2 Inclusion Criteria:**
1. Randomised into the FiTNEss (Myeloma XIV) trial and received induction chemotherapy with ixazomib and lenalidomide continued for 12 cycles.<br><br>2. Achieved at least MR at the end of IRD induction according to the IMWG Uniform Response Criteria for Multiple Myeloma (see Appendix 2), with no evidence of progression prior to R2.<br>3. Meet all of the following blood criteria within 14 days before R2:<br>Haematological:<br>a) Absolute neutrophil count (ANC) ≥ 1 x 109/L. Unless the participant has a known/suspected diagnosis of familial or racial neutropenia in which case an ANC ≥ 0.75 x 109/L is allowed. The use of growth factor support is permitted.<br>b) Platelet count ≥ 50 x 109/L. Please note: Platelet transfusions are not allowed ≤ 3 days prior to randomisation in order to meet these values.<br>c) Haemoglobin ≥ 80 g/L. The use of red blood cell transfusions is permitted.<br><br>Biochemical:<br>d) Total bilirubin ≤ 3 x upper limit of normal (ULN).<br>e) Alanine aminotransferase (ALT) and/or aspartate aminotransferase (AST) [illegible] 3 [illegible] ULN.
**R2 Exclusion Criteria:**
1. Received any anti-myeloma therapy other than their randomised trial treatment, with the exception of local radiotherapy to relieve bone pain (in the absence of disease progression), or bisphosphonate treatment.<br>2. SD or disease progression according to the IMWG Uniform Response Criteria for Multiple Myeloma (see Appendix 2).<br>3. Known resistance, intolerance or sensitivity to ixazomib or lenalidomide that required cessation of either agent during induction.<br>4. Developed any malignancy since R1 except the following:<br>- Adequately treated basal cell or squamous cell skin cancer;<br>- Incidental finding of low grade (Gleason 3+3 or less) prostate cancer requiring no intervention;<br>- Adequately treated carcinoma in situ of the breast or cervix no longer requiring medical or surgical intervention.<br>5. Pregnant, lactating or breastfeeding female participants.<br>6. Major surgery within 14 days before randomisation. This does not include vertebroplasty or kyphoplasty.<br>7. Systemic treatment, within 14 days before the first dose of ixazomib with strong CYP3A inducers (e.g. rifampicin, rifabutin, carbamazepine, phenytoin, phenobarbital), or use of St. John's wort.<br>8. Known gastrointestinal (GI) disease or GI procedure that could interfere with the oral absorption or tolerance of trial treatment, including difficulty swallowing.<br>9. ≥ Grade 2 peripheral neuropathy, or grade 1 with pain.<br>10. Known HIV positive<br>11. Current or known hepatitis B surface antigen positive or hepatitis C antibody positive<br>12. Active systemic infection.<br>13. Any other medical or psychiatric condition which, in the opinion of the investigator, contraindicates the participant's continued participation in this study.<br>14. Receipt of live vaccination within 30 days prior to R1 or receipt of live vaccination at any point during the trial prior to R2.

*MM – Multiple Myeloma, SPCs- Summary of product characteristics

For peer review only

For peer review only - http://bmjopen.bmj.com/site/about/guidelines.xhtml

Table 3: Dosing Schedule

Randomisation 1			
**Treatment**	**Induction – FIT + Induction – standard dosing**	**Induction- Unfit**	**Induction – Frail**
**Lenalidomide (Days = 1-21)**	25mg	15mg	10mg
**Ixazomib (Days = 1,8, 15)**	4mg	4mg	4mg
**Dexamethasone **(Days = 1,8.15, 22)**	40mg in participants <= 75yrs<br>20 mg in participants >75yrs	20mg	10mg
**Randomisation 2**			
**Treatment**	**R+ Placebo Maintenance**	**R + I maintenance**	
**Lenalidomide (Days=1-21)**	10mg**	10mg**	
**Ixazomib (Days=1,8.15)**	N/A	4mg**	
**Placebo (Days=1,8, 15)**	4mg**	N/A	

*Age is consistently used as the participant's age at cycle 1 day 1.
**or final dose administrated at the end of induction treatment if lower

<u>Footnote:</u>

As per standard care, irrespective of R1 allocation, doses of Lenalidomide and Ixazomib will be adjusted at R1 baseline according to liver and renal function. Ixazomib is recommended to be reduced to 3mg in patients with moderate liver dysfunction and with severe renal impairment and end of stage renal disease. No specific dose recommendation have been made for Lenalidomide, however the dose will be as follows: 10mg once daily for moderate renal impairment, 15 mg every other day for severe renal impairment and 5mg once daily at end stage renal disease. Should the dose be tolerated, only the dose of Lenalidomide may be escalated to the maximum dose indicated by the participant's assigned dosing arm/strategy after cycle 1 as per the Lenalidomide SPC.

Ixazomib was not used in general multiple myeloma practice at the time of the EMN (European Myeloma Network) publication. The following licensed dose of Ixazomib will be at induction used for both randomisation arms: 4mg, at days 1, 8 and 15. This has been studied in TNE patients and was well tolerated. There have been no studies examining lower doses of Ixazomib so dose reductions are not permitted out of concern for loss of efficacy. The same dose is used irrespective of frailty.

For peer review only

Myeloma XIV Supplementary Material to Protocol Paper
EudraCT number: **2018-003590-10**

Supplementary Material:

Contents

Glossary of Terms 2
List of Sites in the Fitness Trial 4
Spirit Checklist : Reporting checklist for protocol of a clinical trial. 5
Concomitant Medication 11
Exploratory analysis 12
Subgroup analysis 12
Informed Consent Material 14
Bone marrow and Blood sample consent form 14
Main Trial consent 17
Central Sample Analysis 20
References 21

For peer review only

For peer review only - http://bmjopen.bmj.com/site/about/guidelines.xhtml

Myeloma XIV Supplementary Material to Protocol Paper
EudraCT number: **2018-003590-10**

Glossary of Terms

AE	Adverse event
ADL	Katz Activity of Daily Living
ALT	Alanine aminotransferase
AR	Adverse reaction
ANC	Absolute Neutrophil Count
AST	Aspartate aminotransferase
β2M	Beta2-microglobulin
CCI	Charlson Comorbidity Index
CI	Chief Investigator
CR	Complete response
CRF	Case report form
CTCAE	Common Terminology Criteria for Adverse Events
CTRU	Clinical Trials Research Unit
DMEC	Data Monitoring and Ethics Committee
eCRF	Electronic Case Report Form
GI	Gastrointestinal
HEAP	Health Economics Analysis Plan
HMDS	Haematological Malignancy Diagnostic Service
IADL	Lawton Instrumental Activity of Daily Living
IMiD	Immunomodulatory Drug
IMP	Investigational medicinal product
IMWG	International Myeloma Working Group
ITT	Intention to treat
mg	Milligram
MM	Multiple myeloma
MRD	Minimal Residual Disease
MR	Minimal response
NCI	National Cancer Institute
ORR	Overall response rate
OS	Overall survival
PD	Progressive disease
PFS	Progression-free survival
PFS2	Time from randomisation to second progression
PK	Pharmacokinetic(s)
PI	Proteasome Inhibitor
PR	Partial response
PS	Performance status
QoL	Quality of life
R1	Randomisation 1
R2	Randomisation 2
REC	Research Ethics Committee
SD	Stable disease
SAE	Serious adverse event
SAR	Serious adverse reaction
SPM	Secondary Primary Malignancy
SmPC	Summary of product characteristics

For peer review only - http://bmjopen.bmj.com/site/about/guidelines.xhtml

Myeloma XIV Supplementary Material to Protocol Paper
EudraCT number: **2018-003590-10**

SUSAR	Suspected unexpected serious adverse reaction
TMG	Trial Management Group
TNE	Transplant non-eligible
TSC	Trial Steering Committee
UK-MRA	United Kingdom Myeloma Research Alliance
ULN	Upper Limit of normal
VGPR	Very good partial response
VTE	Venous thromboembolism

For peer review only

For peer review only - http://bmjopen.bmj.com/site/about/guidelines.xhtml

Myeloma XIV Supplementary Material to Protocol Paper
EudraCT number: **2018-003590-10**

List of Sites in the Fitness Trial

Sites		
Aberdeen Royal Infirmary	Kettering general	Salford Royal Hospital
Blackpool Victoria	Kidderminster Hospital	Salisbury District Hospital
Bristol Haematology & Oncology Centre	Kings College Hospital, Denmark Hill	South Tyneside Hospital
Calderdale Royal Hospital	Leicester Royal Infirmary	Southampton Hospital
Castle Hill Hospital	Lincoln County Hospital	Southmead Hospital
Cheltenham Hospital	Maidstone Hospital	St Bartholomew Hospital
Clatterbridge Cancer Centre	Manchester Royal Infirmary	St George's Hospital, Tooting
Colchester District	New Cross Hospital	St Helens Hospital
Countess of Chester	Ninewells	St Helier Hospital
County Hospital	Norfolk & Norwich University Hospital	St Richard's Hospital
Croydon University Hospital	Nottingham City Hospital	St. James's University Hospital
Derriford Hospital	Peterborough City Hospital	St. John's. Hospital, Howden
Dewsbury and District Hospital	Pilgrim Hospital	Sunderland Royal Hospital
Dewsbury and District Hospital	Pontefract Hospital	The County Hospital, Wye Valley
Epsom General Hospital	Princess Royal	Torbay Hospital
Freeman Hospital	Queen Alexandra Hospital	TRAFFORD GENERAL HOSPITAL
Friarage Hospital	Queen Elizabeth Hospital the Queen Mother	University College London Hospital
Glan Clywd	Queen Elizabeth Hospital, Birmingham	Western General
Gloucestershire Royal	Queen Elizabeth Hospital, King's Lynn	Whiston Hospital
Goodhope	Queens Hospital, Romford	William Harvey
Grantham & District	Royal Albert Edward Infirmary	Worcestershire Royal Hospital
Guys Hospital	Royal Berkshire	Worcestershire Royal Hospital
Harrogate District Hospital	Royal Bournemouth General	Worthing Hospital
Heartlands	Royal Derby	Wrexham Maelor Hospital
Hillingdon Hospital	Royal Devon & Exeter Hospital	Wythenshaw Hospital
Hinchingbrooke Hospital	Royal Oldham	YSBYTY GWYNEDD
Huddersfield Royal Infirmary	Royal Stoke University Hospital	North Middlesex University Hospital
James Cook University Hospital	Royal United Hospital, Bath	Royal Hallamshire
Kent & Canterbury Hospital	Russells Hall Hospital	

*In total the trial will be conducted in 87 centres around the UK

For peer review only

Myeloma XIV Supplementary Material to Protocol Paper
EudraCT number: **2018-003590-10**

Spirit Checklist : Reporting checklist for protocol of a clinical trial.

Based on the SPIRIT guidelines.

Chan A-W, Tetzlaff JM, Gøtzsche PC, Altman DG, Mann H, Berlin J, Dickersin K, Hróbjartsson A, Schulz KF, Parulekar WR, Krleža-Jerić K, Laupacis A, Moher D. SPIRIT 2013 Explanation and Elaboration: Guidance for protocols of clinical trials. BMJ. 2013;346:e7586

		Reporting Item	Page Number
**Administrative information**			
Title	#1	Descriptive title identifying the study design, population, interventions, and, if applicable, trial acronym	1
Trial registration	#2a	Trial identifier and registry name. If not yet registered, name of intended registry	1,2
Trial registration: data set	#2b	All items from the World Health Organization Trial Registration Data Set	NA
Protocol version	#3	Date and version identifier	15
Funding	#4	Sources and types of financial, material, and other support	17
Roles and responsibilities: contributorship	#5a	Names, affiliations, and roles of protocol contributors	1, 17
Roles and responsibilities: sponsor contact information	#5b	Name and contact information for the trial sponsor	17
Roles and responsibilities: sponsor and funder	#5c	Role of study sponsor and funders, if any, in study design; collection, management, analysis, and interpretation of data; writing of the report; and the decision to submit the report for publication, including whether they will have ultimate authority over any of these activities	16,17
Roles and responsibilities: committees	#5d	Composition, roles, and responsibilities of the coordinating centre, steering committee, endpoint adjudication committee, data management team, and other individuals or groups overseeing the trial, if applicable (see Item 21a for data monitoring committee)	13
**Introduction**			

For peer review only - http://bmjopen.bmj.com/site/about/guidelines.xhtml

Myeloma XIV Supplementary Material to Protocol Paper
EudraCT number: **2018-003590-10**

Background and rationale	#6a	Description of research question and justification for undertaking the trial, including summary of relevant studies (published and unpublished) examining benefits and harms for each intervention	4,5
Background and rationale: choice of comparators	#6b	Explanation for choice of comparators	4,5,6
Objectives	#7	Specific objectives or hypotheses	6
Trial design	#8	Description of trial design including type of trial (eg, parallel group, crossover, factorial, single group), allocation ratio, and framework (eg, superiority, equivalence, non-inferiority, exploratory)	6
**Methods: Participants, interventions, and outcomes**			
Study setting	#9	Description of study settings (eg, community clinic, academic hospital) and list of countries where data will be collected. Reference to where list of study sites can be obtained	6
Eligibility criteria	#10	Inclusion and exclusion criteria for participants. If applicable, eligibility criteria for study centres and individuals who will perform the interventions (eg, surgeons, psychotherapists)	6
Interventions: description	#11a	Interventions for each group with sufficient detail to allow replication, including how and when they will be administered	7
Interventions: modifications	#11b	Criteria for discontinuing or modifying allocated interventions for a given trial participant (eg, drug dose change in response to harms, participant request, or improving / worsening disease)	7
Interventions: adherance	#11c	Strategies to improve adherence to intervention protocols, and any procedures for monitoring adherence (eg, drug tablet return; laboratory tests)	7
Interventions: concomitant care	#11d	Relevant concomitant care and interventions that are permitted or prohibited during the trial	7

For peer review only - http://bmjopen.bmj.com/site/about/guidelines.xhtml

For peer review only

Outcomes	#12	Primary, secondary, and other outcomes, including the specific measurement variable (eg, systolic blood pressure), analysis metric (eg, change from baseline, final value, time to event), method of aggregation (eg, median, proportion), and time point for each outcome. Explanation of the clinical relevance of chosen efficacy and harm outcomes is strongly recommended	8
Participant timeline	#13	Time schedule of enrolment, interventions (including any run-ins and washouts), assessments, and visits for participants. A schematic diagram is highly recommended (see Figure)	8
Sample size	#14	Estimated number of participants needed to achieve study objectives and how it was determined, including clinical and statistical assumptions supporting any sample size calculations	9
Recruitment	#15	Strategies for achieving adequate participant enrolment to reach target sample size	10
**Methods: Assignment of interventions (for controlled trials)**			
Allocation: sequence generation	#16a	Method of generating the allocation sequence (eg, computer-generated random numbers), and list of any factors for stratification. To reduce predictability of a random sequence, details of any planned restriction (eg, blocking) should be provided in a separate document that is unavailable to those who enrol participants or assign interventions	10 & 11
Allocation concealment mechanism	#16b	Mechanism of implementing the allocation sequence (eg, central telephone; sequentially numbered, opaque, sealed envelopes), describing any steps to conceal the sequence until interventions are assigned	10
Allocation: implementation	#16c	Who will generate the allocation sequence, who will enrol participants, and who will assign participants to interventions	11
Blinding (masking)	#17a	Who will be blinded after assignment to interventions (eg, trial participants, care	11

For peer review only - http://bmjopen.bmj.com/site/about/guidelines.xhtml

		providers, outcome assessors, data analysts), and how	
Blinding (masking): emergency unblinding	#17b	If blinded, circumstances under which unblinding is permissible, and procedure for revealing a participant's allocated intervention during the trial	11
**Methods: Data collection, management, and analysis**			
Data collection plan	#18a	Plans for assessment and collection of outcome, baseline, and other trial data, including any related processes to promote data quality (eg, duplicate measurements, training of assessors) and a description of study instruments (eg, questionnaires, laboratory tests) along with their reliability and validity, if known. Reference to where data collection forms can be found, if not in the protocol	11
Data collection plan: retention	#18b	Plans to promote participant retention and complete follow-up, including list of any outcome data to be collected for participants who discontinue or deviate from intervention protocols	11
Data management	#19	Plans for data entry, coding, security, and storage, including any related processes to promote data quality (eg, double data entry; range checks for data values). Reference to where details of data management procedures can be found, if not in the protocol	11
Statistics: outcomes	#20a	Statistical methods for analysing primary and secondary outcomes. Reference to where other details of the statistical analysis plan can be found, if not in the protocol	12
Statistics: additional analyses	#20b	Methods for any additional analyses (eg, subgroup and adjusted analyses)	13
Statistics: analysis population and missing data	#20c	Definition of analysis population relating to protocol non-adherence (eg, as randomised analysis), and any statistical methods to handle missing data (eg, multiple imputation)	12, 13
**Methods: Monitoring**			

For peer review only - http://bmjopen.bmj.com/site/about/guidelines.xhtml

For peer review only

Myeloma XIV Supplementary Material to Protocol Paper
EudraCT number: **2018-003590-10**

Data monitoring: formal committee	#21a	Composition of data monitoring committee (DMC); summary of its role and reporting structure; statement of whether it is independent from the sponsor and competing interests; and reference to where further details about its charter can be found, if not in the protocol. Alternatively, an explanation of why a DMC is not needed	14
Data monitoring: interim analysis	#21b	Description of any interim analyses and stopping guidelines, including who will have access to these interim results and make the final decision to terminate the trial	14,15
Harms	#22	Plans for collecting, assessing, reporting, and managing solicited and spontaneously reported adverse events and other unintended effects of trial interventions or trial conduct	15
Auditing	#23	Frequency and procedures for auditing trial conduct, if any, and whether the process will be independent from investigators and the sponsor	15
**Ethics and dissemination**			
Research ethics approval	#24	Plans for seeking research ethics committee / institutional review board (REC / IRB) approval	15
Protocol amendments	#25	Plans for communicating important protocol modifications (eg, changes to eligibility criteria, outcomes, analyses) to relevant parties (eg, investigators, REC / IRBs, trial participants, trial registries, journals, regulators)	15
Consent or assent	#26a	Who will obtain informed consent or assent from potential trial participants or authorised surrogates, and how (see Item 32)	16
Consent or assent: ancillary studies	#26b	Additional consent provisions for collection and use of participant data and biological specimens in ancillary studies, if applicable	16
Confidentiality	#27	How personal information about potential and enrolled participants will be collected, shared, and maintained in order to protect confidentiality before, during, and after the trial	16
Declaration of interests	#28	Financial and other competing interests for principal investigators for the overall trial and each study site	17 & 18

For peer review only - http://bmjopen.bmj.com/site/about/guidelines.xhtml

Data access	#29	Statement of who will have access to the final trial dataset, and disclosure of contractual agreements that limit such access for investigators	16
Ancillary and post trial care	#30	Provisions, if any, for ancillary and post-trial care, and for compensation to those who suffer harm from trial participation	16
Dissemination policy: trial results	#31a	Plans for investigators and sponsor to communicate trial results to participants, healthcare professionals, the public, and other relevant groups (eg, via publication, reporting in results databases, or other data sharing arrangements), including any publication restrictions	16
Dissemination policy: authorship	#31b	Authorship eligibility guidelines and any intended use of professional writers	16
Dissemination policy: reproducible research	#31c	Plans, if any, for granting public access to the full protocol, participant-level dataset, and statistical code	16
**Appendices**			
Informed consent materials	#32	Model consent form and other related documentation given to participants and authorised surrogates	13 (Supplementary Material)
Biological specimens	#33	Plans for collection, laboratory evaluation, and storage of biological specimens for genetic or molecular analysis in the current trial and for future use in ancillary studies, if applicable	13 (Supplementary Material)

None The SPIRIT Explanation and Elaboration paper is distributed under the terms of the Creative Commons Attribution License CC-BY-NC. This checklist can be completed online using https://www.goodreports.org/, a tool made by the EQUATOR Network in collaboration with Penelope.ai

For peer review only - http://bmjopen.bmj.com/site/about/guidelines.xhtml

Concomitant Medication

**Permitted Concomitant Medication:**
1) Antiemetics, including 5-HT3 serotonin receptor antagonists, may be used at the discretion of the investigator (however, note that dexamethasone additional to that given as part of the trial treatment schedule is **not** permitted).<br>2) Loperamide or other anti-diarrhoeal should be used for symptomatic non-infective diarrhoea at the discretion of the investigator. The dose and regimen will be according to institutional guidelines. Intravenous fluids should be given to prevent volume depletion.<br>3) Growth factors (e.g. granulocyte colony stimulating factor [G-CSF], thrombopoeitin receptor agonists (TPO) and recombinant erythropoietin) are permitted at the discretion of the PI and/or institutional practice. Due consideration of the increased risk of VTE with TPO agonists and erythropoietin should be given and prophylaxis adjusted accordingly.<br>4) Participants should be transfused with red cells and platelets as clinically indicated and according to institutional guidelines. Platelet transfusions are not allowed within 3 days prior to study drug dosing for any dosing day in order to meet pre-treatment criteria, to ensure that the platelet count is at a safe level to commence further treatment.<br>5) Patients known to be Hep B core antibody positive should receive prophylaxis with an appropriate anti-viral as per local protocols and after consultation with a gastroenterology/hepatology specialist.<br>6) Other supportive measures consistent with optimal participant care may be given throughout the study.
**Excluded concomitant Medication:**
1) Any antineoplastic treatment with activity against MM, other than study drugs and localised radiotherapy for pain relief.<br>2) Platelet transfusions within 3 days prior to study drug dosing for any dosing day in order to meet pre-treatment criteria, or prior to randomisation in order to meet eligibility criteria.<br>3) Additional steroid therapy (not as trial IMP) (except: before commencing trial treatment up to a maximum of 160 mg dexamethasone or equivalent based on glucocorticoid activity per inclusion criteria, or for the management of rash in which case topical, IC or oral steroid at a dose not exceeding prednisolone 10mg per day or equivalent is permitted)<br>4) Radiotherapy for the treatment of symptoms related to disease progression (in the event of disease progression participant should stop trial treatment and further treatment will be at the discretion of the treating clinician).<br>5) Excluded foods and dietary supplements include St. John's Wort.<br>6) Live vaccines are prohibited 30 days prior to R1 and for the duration of the study. They are also prohibited for 3 months after the last dose of study drug.<br>The following treatments should be avoided, unless deemed necessary by the treating clinician:<br>1) Systemic treatment with any of the following metabolising enzyme inducers should be avoided, unless there is no appropriate alternative medication for the participant's use (Rationale: If there were to be a drug-drug interaction with an inducer, ixazomib exposure would be decreased):<br>2) Strong CYP3A inducers: rifampicin, rifabutin, carbamazepine, phenytoin, & phenobarbital<br>3) Non-steroidal anti-inflammatory drugs (NSAIDs) should be avoided especially in those with abnormal renal function unless the clinical benefit outweighs the risk to deteriorating renal impairment.

For peer review only - http://bmjopen.bmj.com/site/about/guidelines.xhtml

Myeloma XIV Supplementary Material to Protocol Paper
EudraCT number: **2018-003590-10**

Exploratory analysis

Exploratory analysis will consider; the association of molecular subgroups with outcome through the investigation of frailty biomarkers, differences between the Karnofsky Performance Status (PS) and ECOG PS, and the prospective validation the UK-MRA MRP (United Kingdom-Myeloma Research Alliance Myeloma Risk Profile). All exploratory analysis to be conducted by the trial statisticians will be pre-specified in the statistical analysis plan.

Subgroup analysis

A series of subgroup analyses will be conducted to determine whether a selection of patient characteristics, haematology / local serological results, biochemistry results and cytogenetic/molecular results as well as response assessments are prognostic of the endpoints; Progression-Free survival, Progression-free survival 2, overall survival, attainment of ≥VGPR (Very Good Partial Response) and attainment of Minimal Residual Disease (MRD) negativity, as defined for both randomisations. Note that the subgroups MRD response to IRD (Ixazomib, Lenalidomide and Dexamethasone) induction and MRD response at 12m maintenance will not be analysed for the attainment of MRD negativity endpoint.

Patient Characteristics

- Age at R1 baseline (<70,70-80,>80)
- Sex (Male / Female)
- ECOG performance status at R1 baseline (0,1, ≥2, missing)
- Karnofsky performance status at R1 baseline (<80, 80-90,>90, missing)
- ISS at R1 baseline (Stage I, Stage II, Stage III, missing)
- R-ISS at R1 baseline (Stage I, Stage II, Stage III, missing)
- IMWG frailty category at R1 baseline (Fit, Unfit, Frail)
- MRP frailty category at R1 baseline (Fit, Intermediate-Fitness, Frail, missing)

Hematology / Local Serological Results

- Haemaglobin concentration at R1 baseline (<100, ≥100 g/L)
- Platelets at R1 baseline (<150, ≥150 x109/L)
- White blood cells at R1 baseline (<LLN, ≥LLN, missing)
- Neutrophil count at R1 baseline (<LLN, ≥LLN)
- Lymphocyte count at R1 baseline (<LLN, ≥LLN, missing)
- Plasma cells (%) in bone marrow at R1 baseline (<60%, ≥60%)

Biochemistry Results

- β-2 microglobulin concentration at R1 baseline (<3.5, 3.5–<5.5, ≥5.5mg/L)
- Serum creatinine concentration at R1 baseline (<175, ≥175 µmol/L)
- Corrected serum calcium concentration at R1 baseline (<2.75, ≥2.75 mmol/L)
- Serum albumin at R1 baseline (<ULN, ≥ULN, missing)
- Lactate dehydrogenase at R1 baseline (<ULN, ≥ULN, missing)
- C-Reactive protein at R1 baseline (<ULN, ≥ULN, missing)
- Total bilirubin at R1 baseline (<ULN, ≥ULN)
- ALT/AST at R1 baseline (<ULN, ≥ULN)

For peer review only - http://bmjopen.bmj.com/site/about/guidelines.xhtml

Myeloma XIV Supplementary Material to Protocol Paper
EudraCT number: **2018-003590-10**

MLPA Cytogenetic Results

- t(4,14) at R1 baseline (detected, not detected)
- t(14,16) at R1 baseline (detected, not detected)
- t(14,20) at R1 baseline (detected, not detected)
- del(17p) at R1 baseline (detected, not detected)
- del(13q) at R1 baseline (detected, not detected)
- del(1p) at R1 baseline (detected, not detected)
- gain(1q) at R1 baseline (detected, not detected)
- UK definition risk group at R1 baseline (Standard, high risk, ultra-high risk)
- IMWG definition risk group at R1 baseline (Adverse, Standard)

Response

- Response to IRD induction (<VGPR, ≥ VGPR)
- Response to 12m maintenance (<VGPR, ≥ VGPR)
- MRD response to IRD induction (MRD positive, MRD Negative)
- MRD response to 12m maintenance (MRD positive, MRD Negative)

Further subgroup analyses may be undertaken, as appropriate and all subgroup analysis will be fully documented in the statistical analysis plan. Subgroup and exploratory analyses may, by chance, generate false negative or positive results. Those carried out will be interpreted with caution.

For peer review only

Myeloma XIV Supplementary Material to Protocol Paper
EudraCT number: **2018-003590-10**

Informed Consent Material

Bone marrow and Blood sample consent form

Delete this line, then print on Trust/Hospital headed paper

Participant ID:	Initials:
Date of Birth:	NHS/Hospital Number:
EudraCT Number:	Principal Investigator:

FiTNEss
UK-MRA Myeloma XIV

PARTICIPANT CONSENT FORM FOR BONE MARROW AND BLOOD SAMPLE

Please initial each box

1. I confirm that I have read and understand the information sheet for the bone marrow and blood sample request and have had the opportunity to ask questions. ☐

2. I understand that my providing bone marrow and blood samples is voluntary and that I am free to withdraw at any time without my medical care or legal rights being affected. ☐

3. I understand that the samples will be sent along with information about me, which will include NHS number and may include my name. ☐

4. I agree to these samples being stored and used for research investigations that form part of the FiTNEss (UK-MRA Myeloma XIV) study, and understand that this will include genetic research. ☐

5. I understand that a unique reference number will be allocated to the samples which may allow them to be linked back to me in future for research purposes. ☐

6. I understand that my data may be shared on a collaborative basis with researchers in the UK and potentially, centres abroad, including outside the European Economic Area. ☐

For peer review only - http://bmjopen.bmj.com/site/about/guidelines.xhtml

Myeloma XIV Supplementary Material to Protocol Paper
EudraCT number: **2018-003590-10**

7. I agree to allow any information provided to be used for healthcare and/or further medical research upon the understanding that my identity will remain anonymous wherever possible. ☐

8. I understand that results of my laboratory tests may be shared with other research teams if I am taking part in another clinical study being run by the University of Leeds. ☐

9. I agree to a copy of this Consent Form being sent to the Clinical Trials Research Unit. ☐

10. I agree for my details (which will include date of birth and NHS number) to be submitted to NHS Digital so that information about my health, and mortality should this become available, may be obtained by the CTRU if necessary. ☐

11. I agree to provide bone marrow and blood samples. ☐

Please indicate your wishes in the below scenarios:

Please tick ✓ or initial

Please tick or initial yes or no:

	Yes	No
A) If I am diagnosed with a plasma cell dyscrasia, other than myeloma, I give permission for the samples sent to the central laboratories to be stored and used in future research that receives ethical approval. I understand that the samples and data collected from them may be shared with researchers, possibly outside the EEA.	☐	☐
B) If I am diagnosed with myeloma but do not take part in the FiTNEss (UK-MRA Myeloma XIV) study, I give permission for the samples sent to the central laboratories to be stored and used in future research that receives ethical approval. I understand that the samples and data collected from them may be shared with researchers, possibly outside the EEA.	☐	☐

For peer review only - http://bmjopen.bmj.com/site/about/guidelines.xhtml

For peer review only

Myeloma XIV Supplementary Material to Protocol Paper
EudraCT number: **2018-003590-10**

Patient (to be completed by the patient):

Signature………..

Name (block capitals)…………………………………………………………………………………………….

Date……..…

Investigator:

I have explained the request to the above named patient and he/she has indicated his/her willingness for samples of bone marrow and blood to be sent to the FiTNEss central laboratories.

Signature………

Name (block capitals)…………………………………………………………………………………………….

Date………

(If used)Translator:

Signature………

Name (block capitals)…………………………………………………………………………………………….

Date………

(1 copy for patient; 1 for the CTRU; 1 held in patient notes, original stored in Investigator Site File)

.

For peer review only

Myeloma XIV Supplementary Material to Protocol Paper
EudraCT number: **2018-003590-10**

Main Trial consent

Delete this line, then print on Trust/Hospital headed paper

Participant ID:	Initials:
Date of Birth:	NHS/Hospital Number:
EudraCT Number:	Principal Investigator:

FiTNEss
UK-MRA Myeloma XIV

PARTICIPANT CONSENT FORM

Please initial each box

1. I confirm that I have read and understand the information sheet for the above study and have had the opportunity to ask questions.

2. I understand that my participation in this study is voluntary and that I am free to withdraw at any time without my medical care or legal rights being affected. I understand that even if I withdraw from the above study, the data and samples collected from me will be used in analysing the results of the study, and in some cases further information about any unwanted effects of my treatment may need to be collected by the study team.

3. I understand that my healthcare records may be looked at by authorised individuals from the study team, regulatory bodies or Sponsor in order to check that the study is being carried out correctly.

4. I understand that I will be asked questions about how independent my lifestyle is. I understand that this information will be used to generate a score of frailty and may affect the doses of treatment that I receive.

5. I am aware that I am required to have samples taken and sent to a central laboratory and I understand that this will include genetic research. I understand that the samples will be sent along with

For peer review only

information about me, which will include NHS number and may include my name.

6. I understand that results or reports from imaging scans, blood tests or other investigations (including for additional cancers) may be sent to the Clinical Trials Research Unit for central review by the research team.

7. I agree to allow any information or results arising from this study to be used for healthcare and/or further medical research upon the understanding that my identity will remain anonymous wherever possible.

8. I understand that my data and results of my laboratory tests (blood, urine and bone marrow sample collections) may be shared with other research teams if I am taking part in another clinical study being run by the University of Leeds.

9. I agree for my details (which will include date of birth and NHS number) to be submitted to NHS Digital so that information about my health, and mortality should this become available, may be obtained by the CTRU if necessary.

10. I understand that my GP, or any other doctor treating me, will be notified of my participation in this study.

11. I understand that some of my data may be passed to other organisations, such as the pharmaceutical companies Takeda and Celgene, (possibly in other countries outside the EEA where the data protection standards and laws may be different from the UK) to monitor the safety of the treatment(s) that I am receiving. I understand that my identity will remain anonymous.

12. I agree to a copy of this Consent Form being sent to the Clinical Trials Research Unit

13. I agree to take part in the study.

For peer review only - http://bmjopen.bmj.com/site/about/guidelines.xhtml

The following parts of the study are OPTIONAL (see part 3 of the Participant Information Sheet). Even if you agree to take part in the FiTNEss study, you do not have to agree to these optional parts. Please tick or initial yes or no.

Please tick ✓ or initial

	Yes	No
A) I agree to take part in the Quality of Life and Healthcare Resource Use studies by completing questionnaires at time points throughout the study.	☐	☐
B) I give permission for samples that I provide to be used in future research that receives ethical approval, and I understand that this includes the blood and bone marrow samples provided at the time of my diagnosis (if consented separately to the bone marrow and blood sample for this study). I understand that my samples and data collected from them may be shared on a collaborative basis with researchers in the UK and potentially, centres abroad, including outside the European Economic Area.	☐	☐

Patient (to be completed by the patient):

Signature…………………………………………………………………………………

Name (block capitals)………………………………………………………………………

Date………………………………………………………………………………………

Investigator:

I have explained the study to the above named patient and he/she has indicated his/her willingness to participate.

Signature…………………………………………………………………………………

Name (block capitals)………………………………………………………………………

Date………………………………………………………………………………………

(If used)Translator:

Signature…………………………………………………………………………………

Name (block capitals)………………………………………………………………………

Date………………………………………………………………………………………

For peer review only - http://bmjopen.bmj.com/site/about/guidelines.xhtml

Myeloma XIV Supplementary Material to Protocol Paper
EudraCT number: **2018-003590-10**

Central Sample Analysis

The trial has four central laboratories, each receiving samples for trial purposes. The time points and the reason for analysis for each central sample can be found in Figure 4. Details on the analysis of each sample is below. Note that participants can consent for their samples to be used in future research and hence each laboratory can store their trial samples for use in future studies. The storage details are also included below.

Birmingham

Samples are sent in approved packaging via the UK post office service and received in a UKAS accredited clinical laboratory for laboratory assessment and storage under the direction of the TMG. Assessment of prognostic markers, markers of disease activity and M-protein levels. Analyses will include serum levels of beta-2 microglobulin, immunoglobulins IgG, IgA and IgM, kappa and lambda free immunoglobulin light chains. Serum electrophoresis and immunofixation, M-protein quantitation including total protein and densitometry. Urine immunofixation and free light chain quantitation will be performed where appropriate.

HMDS

The presence of minimal residual disease will be assessed in bone marrow aspirates using a validated flow cytometry assay (sensitivity ≤0.004%) performed at a single central laboratory (HMDS, Leeds Teaching Hospitals Trust). A minimum of 500,000 cells will be evaluated with six- or eight-colour antibody combinations including CD138, CD38, CD45, CD19, CD56, CD27, CD81 and CD117.

Samples will be used for trial purposes only and will not be stored for future research.

ICR

Myeloma tumor material is purified from fresh bone marrow material using CD138 immunomagnetic separation. Tumor DNA and RNA are extracted and recurrent immunoglobulin translocations t(4;14), t(14;16), t(14;20) and copy number aberrations del(1p32), gain(1q21), del(17p) investigated as previously published using qRT-PCR and multiplex ligation-dependent amplification (MLPA), respectively[1]. Excess material is stored, if patient gave written consent for use in future research, at -80C standard DNA and RNA storage conditions.

LIMR

Peripheral blood samples for immunosenescent and Senescence-associated secretory proteome will be draw and managed timepoints in the clinical pathway, processed in a central laboratory and stored as aliquots of mononuclear cells and serum for subsequent planned use. Full QA is performed on sample receipt to ensure only viable and valuable samples are processed and stored for research use.

For peer review only - http://bmjopen.bmj.com/site/about/guidelines.xhtml

References

1. Shah, V., et al., *Prediction of outcome in newly diagnosed myeloma: a meta-analysis of the molecular profiles of 1905 trial patients.* Leukemia, 2018. **32**(1): p. 102-110.

For peer review only

For peer review only - http://bmjopen.bmj.com/site/about/guidelines.xhtml

BMJ Open

Frailty-adjusted therapy in Transplant Non-Eligible patients with newly diagnosed Multiple Myeloma (FiTNEss (UK-MRA Myeloma XIV Trial)): A study protocol for a randomised phase III trial

Journal:	*BMJ Open*
Manuscript ID	bmjopen-2021-056147.R1
Article Type:	Protocol
Date Submitted by the Author:	25-Jan-2022
Complete List of Authors:	Coulson, Amy; University of Leeds Clinical Trials Research Unit, Leeds Institute of Clinical Trials Research<br>Royle, Kara-Louise; University of Leeds Clinical Trials Research Unit, Leeds Institute of Clinical Trials Research<br>Pawlyn, Charlotte; Cancer Research UK London Research Institute<br>Cairns, David; University of Leeds Clinical Trials Research Unit, Leeds Institute of Clinical Trials Research<br>Hockaday, Anna; University of Leeds Clinical Trials Research Unit, Leeds Institute of Clinical Trials Research<br>Bird, Jennifer; University Hospitals Bristol and Weston NHS Foundation Trust, Department of Haematology<br>Bowcock, Stella; Princess Royal Hospital, Department of Haematology, Kings college Hospital NHS Foundation Trust<br>Kaiser, Martin; Institute of Cancer Research; Royal Marsden Hospital NHS Trust, The Department of Haemato-oncology<br>de Tute, Ruth; University of Leeds, Haematology Malignancy Diagnostic Service (HMDS)<br>Rabin, Neil; University College London Hospitals NHS Foundation Trust, Department of Haematology<br>Boyd, Kevin; Royal Marsden Hospital NHS Trust, The Department of Haemato-oncology<br>Jones, John; King's College Hospital; Eastbourne District General Hospital<br>Parrish, Christopher; Leeds Teaching Hospitals NHS Trust, Department of Haematology<br>Gardner, Hayley; University Hospitals Birmingham NHS Foundation Trust, Department of Haematology and Stem Cell Transplantation<br>Meads, David; University of Leeds Leeds Institute of Health Sciences, Academic Unit of Health Economics<br>Dawkins, Bryony ; University of Leeds Leeds Institute of Health Sciences, Academic Unit of Health Economics<br>Olivier, Catherine; University of Leeds Clinical Trials Research Unit, Leeds Institute of Clinical Trials Research<br>Henderson, Rowena; University of Leeds Clinical Trials Research Unit, Leeds Institute of Clinical Trials Research<br>Best, Phillip; University of Leeds Clinical Trials Research Unit, Leeds Institute of Clinical Trials Research<br>Owen, Roger; St James's University Hospital, Haematology Malignancy

	Diagnostic Service (HMDS)<br>Jenner, Matthew ; Southampton General Hospital<br>Kishore, Bhuvan; University Hospitals Birmingham NHS Foundation Trust, Department of Haematology and Stem Cell Transplantation,<br>Drayson, Mark ; University of Birmingham, Institute of Immunology and Immunotherapy University of Birmingham Department of Haematology<br>Jackson, Graham; Newcastle University, Department of Haematology<br>Cook, Gordon; University of Leeds Clinical Trials Research Unit, Leeds Institute of Clinical Trials Research; St James's University Hospital, Leeds Cancer Centre
**Primary Subject Heading**:	Haematology (incl blood transfusion)
Secondary Subject Heading:	Haematology (incl blood transfusion), Qualitative research, Health economics, Oncology
Keywords:	Myeloma < HAEMATOLOGY, Myeloma < ONCOLOGY, CHEMOTHERAPY, Clinical trials < THERAPEUTICS

SCHOLARONE™
Manuscripts

Frailty-adjusted therapy in Transplant Non-Eligible patients with newly diagnosed Multiple Myeloma (FiTNEss (UK-MRA Myeloma XIV Trial)): A study protocol for a randomised phase III trial

Amy Beth Coulson[1], Kara-Louise Royle[1], Charlotte Pawlyn[2], David A Cairns[1], Anna Hockaday[1], Jennifer Bird[3], Stella Bowcock[4], Martin Kaiser[2,5], Ruth De Tute[6], Neil Rabin[7], Kevin Boyd[5], John Jones[15,16,17], Christopher Parrish[14], Hayley Gardner[9], David Meads[10], Bryony Dawkins[10], Catherine Olivier[1], Rowena Henderson[1], Phillip Best[1], Roger Owen[6], Matthew Jenner[11], Bhuvan Kishore[9], Mark Drayson[12], Graham Jackson[13] and Gordon Cook[1,8*]

1. Clinical Trials Research Unit, Leeds Institute of Clinical Trials Research, University of Leeds, Leeds, United Kingdom
2. The Institute of Cancer Research, London, United Kingdom
3. Department of Haematology, University Hospitals Bristol and Weston NHS Trust , Bristol, UK
4. Department of Haematology, King's College Hospital NHS Foundation Trust, Princess Royal Hospital, London, UK
5. The Department of Haemato-oncology, The Royal Marsden NHS Foundation Trust, London, UK
6. HMDS, St James's University Hospital, Leeds, UK
7. The Department of Haematology, University College London Hospitals NHS Trust, London
8. Leeds Cancer Centre, St James's University Hospital, Leeds, United Kingdom
9. Department of Haematology and Stem Cell Transplantation, University Hospitals Birmingham NHS Foundation Trust, Birmingham
10. Academic Unit of Health Economics, Leeds Institute of Health Sciences, University of Leeds, Leeds, UK
11. University Hospital Southampton NHS Foundation Trust
12. Institute of Immunology and Immunotherapy University of Birmingham Department of Haematology
13. Department of Haematology, Newcastle University, Newcastle-upon-Tyne, United Kingdom
14. Department of Haematology, Leeds Teaching Hospitals NHS Trust, Leeds, UK
15. Brighton and Sussex Medical School, Brighton, UK
16. King's College Hospital, London, UK.
17. Eastbourne District General Hospital, UK.

*Corresponding Author:

Gordon Cook, MB.ChB, PhD, FRCP(Glas), FRCPath

Leeds Cancer Centre

St James's University Hospital

Beckett Street, Leeds, UK, LS9 7TF

Tel: +44 113 206 7940 Fax: +44 113 206 8177

E-mail: g.cook@leeds.ac.uk

Word count: 6558

For peer review only - http://bmjopen.bmj.com/site/about/guidelines.xhtml

Abstract

Introduction

Multiple myeloma is a bone marrow cancer which predominantly affects older people. The incidence is increasing in an ageing population.

Over the last ten years patient outcomes have improved. However, this is less apparent in older, less fit patients, who are ineligible for stem cell transplant. Research is required in this patient group, taking into account frailty and aiming to improve: treatment tolerability, clinical outcomes and quality of life.

Methods and analysis

FiTNEss is a national, phase III, multicentre, randomised controlled trial comparing standard (reactive) and frailty-adjusted (adaptive) induction therapy delivery with ixazomib, lenalidomide and dexamethasone (IRD), and to compare maintenance lenalidomide to lenalidomide plus ixazomib, in patients with newly diagnosed multiple myeloma not suitable for stem cell transplant. Seven-Hundred and Forty participants will be registered into the trial to allow 720 and 478 to be randomised at induction and maintenance, respectively.

All participants will receive IRD induction with the dosing strategy randomised (1:1) at trial entry. Patients randomised to the standard, reactive arm will commence at the full dose followed by toxicity dependent reactive modifications. Patients randomised to the adaptive arm will commence at a dose level determined by their International Myeloma Working Group (IMWG) frailty score. Following 12 cycles of induction treatment participants alive and progression-free will undergo a second (double-blind) randomisation on a 1:1 basis to maintenance treatment with lenalidomide plus placebo versus lenalidomide plus ixazomib until disease progression or intolerance.

Ethics and dissemination

Ethical approval has been obtained from the North East - Tyne & Wear South Research Ethics Committee (19/NE/0125) and capacity and capability confirmed by local research and development departments for each participating centre prior to opening to recruitment. Participants are required to provide written informed consent prior to trial registration. Trial results will be disseminated by conference presentations and peer-reviewed publications.

Trial registration

ISRCTN17973108

ClinicalTrials.gov:NCT03720041.

Key Words: Multiple Myeloma, Transplant-ineligible, Frailty-adjusted, Randomised, Blinded

For peer review only - http://bmjopen.bmj.com/site/about/guidelines.xhtml

Article Summary

Strengths and limitations of this Study

- FiTNEss will provide the first prospective data investigating the use of the International Myeloma Working Group (IMWG) frailty score to define appropriate dose delivery strategies for older patients.
- FiTNEss will explore the impact of dual agent maintenance compared to the single agent standard of care using a gold standard placebo controlled design.
- The trial has the potential to meet a high unmet need in older myeloma patients for whom the impact of recent therapeutic have been less marked.
- Wide inclusion criteria have been designed to maximise the recruitment of older, more frail patients who may not previously have been included in clinical trials.
- Owing to the nature of assessments and dose adaptations blinding in the induction phase of the trial is infeasible.

For peer review only

For peer review only - http://bmjopen.bmj.com/site/about/guidelines.xhtml

Introduction

Multiple Myeloma

Multiple Myeloma is the second most common haematological malignancy with over 5500 patients diagnosed in the UK each year.[1] Myeloma is predominantly a disease of older people, with two thirds of patients aged over 70 years at diagnosis. The incidence is increasing as the population ages.

Over the last ten years the development of proteasome inhibitors (PI), immunomodulatory drugs (IMiD® agents) and improved supportive care, have ameliorated outcomes for myeloma patients such that the median overall survival is now more than 6 years for younger, fitter patients (Figure 1A).[2] However, the impact of these therapies has been less marked in the older or less fit population, particularly those over 75 years of age and/or ineligible for stem cell transplant (Figure 1B). Whilst outcomes in younger patients are largely driven by molecular risk factors present in the myeloma cell clone, there is no evidence that older myeloma patients have more biologically high-risk disease.[3] Differences in outcomes are likely to be accounted for by changes in patient physiology and/or increased treatment-related toxicity. Data from our previous trial, Myeloma XI (ISRCTN49407852) shows that as age increases the number of participants ceasing treatment due to choice or toxicity increases (Figure 2). This group therefore has a high unmet need for new, less toxic treatments and improved treatment delivery approaches.

Existing Evidence: Induction Therapy

Treatment

Lenalidomide is an immunomodulatory agent and thalidomide derivative available as an oral preparation, which is more potent *in vitro* and with a different adverse effect profile than thalidomide. For example, a major benefit of lenalidomide is the absence of associated neurotoxicity or sedation seen with thalidomide, making it more tolerable; however, there is a significant rate of myelosuppression (20%), which is not seen with thalidomide. Lenalidomide is licensed, in combination with dexamethasone, for use in newly diagnosed multiple myeloma patients who are not eligible for transplant (TNE).

With the caveats of cross-trial comparison, our recent analysis of the Myeloma XI trial suggests that the combination CRDa (cyclophosphamide, lenalidomide and attenuated dexamethasone) is not superior to Rd-continuous used in the FIRST study (NCT00689936) in terms of progression-free survival (PFS). In addition, Myeloma XI also showed that patients in the non-intensive pathway with only a minimal or partial response to induction therapy who were randomised to receive cyclophosphamide, bortezomib and dexamethasone (CVD) consolidation had better outcomes than those who did not (an increase in median PFS of 11 months (HR: 0.73; P=0.061)).[4] However, a significant proportion of patients (394/610; 64.6%) did not make it to this randomisation due to participant withdrawal (211; 53.6% of 394), death (96; 24.4%), progression (45; 11.4%) and other reasons (42; 10.7%). These data suggest that combining IMiD® agents and PI upfront may improve outcomes further, avoiding the early loss of patients and leading to improved responses, as we have seen with the carfilzomib, cyclophosphamide, lenalidomide and dexamethasone (KCRD) arm in the transplant-eligible pathway of the Myeloma XI study.[5 6]

Ixazomib (MLN9708) is an orally bioavailable, small molecule inhibitor of the 20S proteasome, and has shown single agent activity in phase I/II studies alongside combination therapy with dexamethasone and more recently IMiD agents and alkylating agents.[7-9] The oral formulation provides convenience

For peer review only - http://bmjopen.bmj.com/site/about/guidelines.xhtml

for patients, and the slower pharmacokinetic (PK) profile reduces the neurotoxicity seen with bortezomib, suggesting the use of this new PI in combination regimens might be better tolerated.[10] In the front-line setting the combination of ixazomib with lenalidomide and dexamethasone (IRD) has been reported in phase I/II studies demonstrating high response rates and good tolerability.[11]

With a favourable toxicity profile compared to either carfilzomib or bortezomib, and the benefits of oral dosing, the IRD combination represents a tolerable regimen to achieve the same combination of IMiD® agents and PI in TNE patients. The Tourmaline MM-1 study demonstrated excellent efficacy and tolerability of IRD in the relapsed setting in TNE patients, supporting this hypothesis.[12] The Tourmaline MM-02 study, a randomised, double blind, placebo controlled study evaluating IRd vs placebo Rd has recently been reported[13] and showed a clinically meaningful but non-significant 13.5 month improvement in PFS in the IRd group (35.3 vs 21.8 months; HR 0.83; P = 0.073). This trial used reactive dosing strategies.

Treatment Delivery

The International Myeloma Working Group (IMWG) proposed a scoring system for myeloma patient frailty that predicts survival, adverse events (AEs) and treatment tolerability,[14] which can help to account for the considerable heterogeneity in outcome for TNE patients. This score combines age and the outcomes of 3 patient assessment tools; the Katz Activity of Daily Living (ADL),[15] Lawton's Instrumental Activity of Daily Living (IADL),[16] and the Charlson Comorbidity Index (CCI)[17 18] to categorise patients into three groups: fit, unfit and frail. The IMWG frailty score was subsequently shown to be predictive of both PFS and toxicity. An increase in frailty score was associated with an increased risk of death, progression, non-haematological AEs and treatment discontinuation that was independent of classical definitions of risk, including ISS stage and cytogenetic risk, and also independent of treatment regimen. As such it was suggested to be useful in determining the feasibility of treatment regimens and appropriate dose reductions, but this remains to be prospectively validated.

Existing Evidence: Maintenance Therapy

Four published studies have demonstrated an important clinical benefit for the use of maintenance lenalidomide in newly diagnosed multiple myeloma in patients of all ages. Data from our previous study Myeloma XI contributes to this evidence base and in TNE patients, lenalidomide maintenance demonstrated a significant improvement in PFS compared to observation, of 24 months vs 11 months from maintenance randomisation.[19] This improvement was seen across all subgroups of multiple myeloma patients including all cytogenetic risk groups and at all ages. Overall survival data demonstrated a benefit for lenalidomide once the effect of subsequent therapies have been taken in to account.[20]

Overall, the data for maintenance lenalidomide until disease progression in patients not eligible for stem cell transplant suggest that there is a clear and significant improvement in PFS and a possible OS benefit. The crucial question to answer, going forward, is whether the results seen with lenalidomide as a single agent for maintenance can be enhanced further by the use of a combination regimen.

The use of ixazomib monotherapy in the maintenance setting demonstrated efficacy and tolerability in previously untreated patients [21] and this has recently been confirmed in the Phase III Tourmaline-MM4 trial in the non-transplant-eligible setting,[22] Adding ixazomib to lenalidomide maintenance has not been studied in a randomised phase III study.

For peer review only - http://bmjopen.bmj.com/site/about/guidelines.xhtml

Existing Evidence: Summary

The recently developed, less-toxic PI ixazomib [11] with novel IMiD® agents/PI combinations for induction and maintenance treatment needs to be evaluated in the context of the IMWG frailty score where frailty-adjusted dosing was recommended,[14] but emphasised the need for prospective validation of their approach.

Aims and Objectives

The FiTNEss trial aims to improve outcomes for TNE patients by investigating whether using prospective dose adjustments dependent on patient frailty will improve patients' ability to remain on therapy, reduce toxicity, and improve outcomes from randomisation 1 (R1). The trial also aims, from randomisation 2 (R2), to investigate whether doublet maintenance therapy improve outcomes compared to single agent lenalidomide without prohibitive toxicity.

Trial Design

The FiTNEss trial is a phase III, multi-centre, randomised, parallel group trial in newly diagnosed patients with MM, who are assessed to be TNE by their treating clinician. Following R2 the trial is also double-blind placebo-controlled with the participant and treating clinician blind to treatment allocation. The following report details the trial protocol and follows the structure of the SPIRIT statement.[23] The SPIRIT checklist[24] can be found within the supplementary material.

For peer review only

Methods

Setting

The trial will be conducted at 87 centres around the United Kingdom (See supplementary material), as identified via a feasibility assessment to determine the most appropriate to participate in the trial. The majority of potential participants will be identified by the research team at the time they are referred to the haematology outpatient department with suspected multiple myeloma. A smaller number of participants may be identified during inpatient admissions. Invitation to participate in the trial and provision of information will be made either during their first consultation, when routine diagnostic tests will be performed and potential treatment options discussed, or at the time they receive their diagnostic test results.

Eligibility criteria

Adults (18 years and older) with newly diagnosed MM, by IMWG 2014 diagnostic criteria, [25] who are TNE and who are capable of giving written informed consent will be assessed for eligibility. Eligibility will be confirmed prior to each randomisation by the principal investigator or authorised delegate and will be recorded in the participant's medical records and on the relevant case report form (CRF). The participant will be registered into the trial prior to undergoing procedures that are specifically for the purposes of the trial and are above NHS standard of care.

To be eligible for R1, participants must meet all the inclusion criteria and none of the exclusion criteria outlined in Table 1. Following 12 cycles of induction therapy, participants who achieve at least a minimal response (MR), according to IMWG uniform response criteria, and fulfill all the inclusion criteria and none of the exclusion criteria outlined in Table 2, will proceed to R2.

For peer review only - http://bmjopen.bmj.com/site/about/guidelines.xhtml

Table 1: Randomisation 1 Inclusion and Exclusion Criteria

Inclusion Criteria:

1. Newly diagnosed as having MM according to the updated IMWG diagnostic criteria 2014 requiring treatment.
2. Not eligible for stem cell transplant.
3. Aged at least 18 years.
4. Meet all of the following blood criteria within 14 days before R1:

Haematological:

a) Absolute neutrophil count (ANC) ≥ 1 x 109/L. Unless the participant has a known/suspected diagnosis of familial or racial neutropenia in which case an ANC ≥ 0.75 x 109/L is allowed. The use of growth factor support is permitted.

b) Platelet count ≥ 50 x 109/L, or, in the case of heavy bone marrow infiltration (≥ 50%) which in the opinion of the investigator is the cause of the thrombocytopenia and provided appropriate supportive measures and patient monitoring are in place, platelet count ≥ 30x 109/L is permitted. Please note: Platelet transfusions are not allowed ≤ 3 days prior to randomisation in order to meet these values.

c) Haemoglobin ≥ 80 g/L. The use of red blood cell transfusions is permitted.

Biochemical:

d) Total bilirubin ≤ 3 x upper limit of normal (ULN).

e) Alanine aminotransferase (ALT) and/or aspartate aminotransferase (AST) ⍰ 3 ⍰ ULN.

5. Meet the pregnancy prevention requirements:

Female participants who:

a) Are not of childbearing potential, OR

b) If they are of childbearing potential, agree to practice 2 effective methods of contraception, at the same time, from the time of signing the informed consent form until 90 days after the last dose of study drug, OR

c) Agree to practice true abstinence when this is in line with the preferred and usual lifestyle of the subject. (Periodic abstinence [e.g. calendar, ovulation, symptothermal, post-ovulation methods] and withdrawal are not acceptable methods of contraception.)

Male participants, even if surgically sterilised (i.e. status post-vasectomy), must agree to one of the following:

a) Agree to practice effective barrier contraception during the entire study treatment period and through 90 days after the last dose of study drug, OR

b) Agree to practice true abstinence when this is in line with the preferred and usual lifestyle of the subject. (Periodic abstinence (e.g. calendar, ovulation, symptothermal, post-ovulation methods] and withdrawal are not acceptable methods of contraception).

Contraception for female and male participants must be in accordance with (and participants must consent to) the Celgene-approved Pregnancy Prevention Programme.

If female and of childbearing potential, they must have a negative pregnancy test performed by a healthcare professional in accordance with the Celgene Pregnancy Prevention Programme.

6. Able to provide written informed consent.

Exclusion Criteria:

1. Smouldering MM, MGUS, solitary plasmacytoma of bone, or extramedullary plasmacytoma (without evidence of MM).
2. Received previous treatment for MM, with the exception of local radiotherapy to relieve bone pain or spinal cord compression, prior bisphosphonate treatment, or corticosteroids as long as the total dose does not exceed the equivalent of 160 mg dexamethasone.
3. Known resistance, intolerance or sensitivity to any component of the planned therapies.
4. Prior or concurrent invasive malignancies except the following:

- Adequately treated basal cell or squamous cell skin cancer;
- Incidental finding of low grade (Gleason 3+3 or less) prostate cancer requiring no intervention;
- Adequately treated carcinoma in situ of the breast or cervix no longer requiring medical or surgical intervention;
- Any cancer from which the subject has been disease-free for at least 3 years.

5. Pregnant, lactating or breastfeeding female participants.
6. Major surgery within 14 days before randomisation. This would include surgical intervention for relief of cord compression but does not include vertebroplasty or kyphoplasty.

For peer review only - http://bmjopen.bmj.com/site/about/guidelines.xhtml

7. Systemic treatment, within 14 days before the first dose of ixazomib with strong CYP3A inducers (e.g. rifampicin, rifabutin, carbamazepine, phenytoin, phenobarbital), or use of St. John's wort.

8. Any concomitant drug therapy which, in the opinion of the investigator, may lead to an unacceptable interaction with any of the agents ixazomib, lenalidomide, dexamethasone, and that cannot be safely stopped prior to trial entry. Full details of interactions can be found in the SPCs.

9. Known gastrointestinal (GI) disease or GI procedure that could interfere with the oral absorption or tolerance of trial treatment, including difficulty swallowing.

10. ≥ Grade 2 peripheral neuropathy.

11. Known HIV positive

12. Participant has current or prior hepatitis B surface antigen positive or hepatitis C antibody positive. Participants must have screening conducted within 14 days before R1.

13. Active systemic infection.

14. Any other medical or psychiatric condition which, in the opinion of the investigator, contraindicates the participant's participation in this study.

15. Receipt of live vaccination within 30 days prior to R1.

Table 2: Randomisation 2 Inclusion ad Exclusion Criteria

R2 Inclusion Criteria:

1. Randomised into the FiTNEss (Myeloma XIV) trial and received induction chemotherapy with ixazomib and lenalidomide continued for 12 cycles.

2. Achieved at least MR at the end of IRD induction according to the IMWG Uniform Response Criteria for Multiple Myeloma, with no evidence of progression prior to R2.

3. Meet all of the following blood criteria within 14 days before R2:
Haematological:
a) Absolute neutrophil count (ANC) ≥ 1 x 109/L. Unless the participant has a known/suspected diagnosis of familial or racial neutropenia in which case an ANC ≥ 0.75 x 109/L is allowed. The use of growth factor support is permitted.
b) Platelet count ≥ 50 x 109/L. Please note: Platelet transfusions are not allowed ≤ 3 days prior to randomisation in order to meet these values.
c) Haemoglobin ≥ 80 g/L. The use of red blood cell transfusions is permitted.

Biochemical:
d) Total bilirubin ≤ 3 x upper limit of normal (ULN).
e) Alanine aminotransferase (ALT) and/or aspartate aminotransferase (AST) □ 3 □ ULN.

R2 Exclusion Criteria:

1. Received any anti-myeloma therapy other than their randomised trial treatment, with the exception of local radiotherapy to relieve bone pain (in the absence of disease progression), or bisphosphonate treatment.

2. SD or disease progression according to the IMWG Uniform Response Criteria for Multiple Myeloma.

3. Known resistance, intolerance or sensitivity to ixazomib or lenalidomide that required cessation of either agent during induction.

4. Developed any malignancy since R1 except the following:

- Adequately treated basal cell or squamous cell skin cancer;
- Incidental finding of low grade (Gleason 3+3 or less) prostate cancer requiring no intervention;
- Adequately treated carcinoma in situ of the breast or cervix no longer requiring medical or surgical intervention.

5. Pregnant, lactating or breastfeeding female participants.

6. Major surgery within 14 days before randomisation. This does not include vertebroplasty or kyphoplasty.

7. Systemic treatment, within 14 days before the first dose of ixazomib with strong CYP3A inducers (e.g. rifampicin, rifabutin, carbamazepine, phenytoin, phenobarbital), or use of St. John's wort.

8. Known gastrointestinal (GI) disease or GI procedure that could interfere with the oral absorption or tolerance of trial treatment, including difficulty swallowing.

9. ≥ Grade 2 peripheral neuropathy, or grade 1 with pain.

10. Known HIV positive

For peer review only

11. Current or known hepatitis B surface antigen positive or hepatitis C antibody positive
12. Active systemic infection.
13. Any other medical or psychiatric condition which, in the opinion of the investigator, contraindicates the participant's continued participation in this study.
14. Receipt of live vaccination within 30 days prior to R1 or receipt of live vaccination at any point during the trial prior to R2.

Interventions and Dosing

Intervention Schedule

The control and experimental interventions for induction (R1) and maintenance (R2) therapy include: lenalidomide, ixazomib, placebo and dexamethosone. At R1, eligible patients will be allocated to one of two interventions with IRD induction; standard up-front dosing followed by toxicity dependent dose modification (reactive), or; frailty score-adjusted up-front dose reductions (adaptive). At R2, eligible patients will be randomised between lenalidomide + ixazomib (R+I) or lenalidomide and placebo maintenance therapy. Table 3 summarises the dosing schedules for R1 and R2.

Table 3: Dosing Schedule[1]

Randomisation 1			
**Treatment**	**Induction – FIT + Induction – standard dosing**	**Induction- Unfit**	**Induction – Frail**
**Lenalidomide (Days = 1-21)**	25mg	15mg	10mg
**Ixazomib (Days = 1,8, 15)**	4mg	4mg	4mg
**Dexamethasone **(Days = 1,8.15, 22)**	40mg in participants <= 75yrs<br>20 mg in participants >75yrs	20mg	10mg
**Randomisation 2**			
**Treatment**	**R+ Placebo Maintenance**	**R + I maintenance**	
**Lenalidomide (Days=1-21)**	10mg**	10mg**	
**Ixazomib (Days=1,8.15)**	N/A	4mg**	
**Placebo (Days=1,8, 15)**	4mg**	N/A	

*Age is consistently used as the participant's age at cycle 1 day 1.
**or final dose administrated at the end of induction treatment if lower

[1] As per standard care, irrespective of R1 allocation, doses of Lenalidomide and Ixazomib will be adjusted at R1 baseline according to liver and renal function. Regardless of which dosing arm has been assigned the doses indicated to be adjusted for as per renal and liver impairment should not be exceeded on cycle 1 day 1. For renal function, Creatinine clearance should be calculated using the Cockcroft-Gault formula. For moderate renal impairment, ixazomib should be given 4mg daily and should be reduced be reduced to 3mg in patients with severe renal impairment and end of stage renal disease. For moderate renal impairment, 10mg of Lenalidomide once daily should be given,, 15 mg every other day for severe renal impairment and 5mg once daily at end stage renal disease. Doses of lenalidomide reduced (based on renal function) can only be escalated, if tolerated, to the maximum dose indicated by the participant's assigned dosing arm/strategy after cycle 1 as per the lenalidomide SPC. Ixazomib doses may **not** be escalated, whether reduced based on renal or liver function. For liver function, the reduced ixazomib dose of 3 mg is recommended in patients with moderate liver dysfunction, Lenalidomide has not formally been studied in patients with impaired hepatic function and there are no specific dose recommendations. Further information regarding dosing due to liver and renal function is provided in supplementary material.

Ixazomib was not used in general multiple myeloma practice at the time of the EMN (European Myeloma Network) publication. The following licensed dose of Ixazomib will be at induction used for both randomisation arms: 4mg, at days 1, 8 and 15. This has been studied in TNE patients and was well tolerated. There have been no studies examining lower doses of Ixazomib so dose reductions are not permitted out of concern for loss of efficacy. The same dose is used irrespective of frailty.

For peer review only - http://bmjopen.bmj.com/site/about/guidelines.xhtml

As part of their induction therapy, participants in the adaptive arm of R1 will have their dose adjusted according to changes in frailty category at the start of cycles 3, 5 and 7[2]. Doses can also be escalated for suboptimal responders under certain criteria. If after cycle 2, a participant on the unfit or frail dosing strategy has not achieved at least an MR, or required a dose reduction due to toxicity, a request can be made to increase any of their doses to the next highest level at the start of cycle 4. A similar request can be made after cycle 4 for the start of cycle 6, but this requires at least a partial response (PR). This criteria applies for all participants irrespective of changes in frailty at cycles 3 and 5.

Intervention Adherence

Throughout the trial lenalidomide and ixazomib will be taken orally and swallowed whole at the same time on the scheduled days. Dexamethasone will be administrated in accordance with the relevant Summary of Product Characteristics (SPC). To monitor treatment adherence, participants will complete a daily medication diary which will be reviewed at trial visits. Unused capsules will be returned to pharmacy.

Dose Modification and Discontinuation

Both R1 and R2 treatment cycles will be 28 days in length. Response will be assessed at the end of each cycle according to the IMWG 2016 Uniform Response Criteria [26 27]. In the absence of progression or treatment intolerance, participants will receive a maximum of 12 cycles of induction therapy and continuous maintenance therapy. Those who receive 12 cycles of induction therapy will be assessed for R2 eligibility, with non-eligible participants being treated off trial.

Toxicity and hence treatment intolerance will be assessed throughout each treatment cycle, according to the National Cancer Institute (NCI) common terminology criteria for AE (CTCAE) Version 5.

For a new cycle of treatment to begin (induction and maintenance), the participant must meet the haematological and biochemical criteria (at day 1 or ≤3 days prior) outlined in the eligibility criteria (R1: Table 1, R2: Table 2). Non-haematological toxicities (except for alopecia) must have resolved to less than or equal to Grade 1 or to the participants baseline condition in order for treatment to resume. If the participant does not meet this criteria their dose will be delayed for one week before they are re-assessed. This will continue for a maximum of three weeks before treatment discontinuation or eight weeks at the discretion of the Chief Investigator.

In the event that a dose is reduced due to toxicity, as per the lenalidomide SPC the dose may be reintroduced to the next higher dose level upon improvement. Ixazomib, once reduced cannot be re-escalated.

Concomitant medication

Concomitant medication, disease and other malignancies will be recorded at eligibility.

All participants may receive additional care during the treatment period as deemed appropriate by the treating clinician. Local support care protocols, including anti-emetic schedules, tumor lysis syndrome prevention, VTE (Venous thromboembolism) prophylaxis and prophylactic antimicrobial therapy, will be followed for both randomisations. Permitted and excluded concomitant medications and procedures can be found in the supplementary material.

---

[2] For the Myeloma Frailty index, participant's age is at the time of (Main) Trial registration, therefore, a patient's frailty will never change based on age only.

For peer review only - http://bmjopen.bmj.com/site/about/guidelines.xhtml

For peer review only

Outcomes

Primary outcome

For R1 the primary outcome is early treatment cessation (defined at a binary endpoint) for reactive versus adaptive dosing in participants defined to be "unfit" or "frail" at baseline. Participants will be defined to have experienced an event if they die, progress, or are withdrawn from treatment (by the treating clinician) or withdraw consent for treatment, within 60 days of R1.

For R2 the primary outcome is PFS for R+Placebo versus R+I, and is defined as the time from R2 to the time of first documented disease progression or death from any cause. Individuals lost to follow up or progression-free at time of analysis will be censored at their last known alive and progression-free date.

Secondary outcomes

The secondary outcomes of this trial are to assess PFS for reactive versus adaptive dosing, time to progression, time to 2nd PFS event (PFS2), overall survival (OS), survival after progression, deaths within 12 months of R1, overall response rate (ORR), attainment of ≥VGPR, attainment of MRD negativity[3], duration of response, time to improved response, time to next treatment, treatment compliance and total amount of therapy delivered, toxicity and safety including the incidence of Second Primary Malignancies (SPMs), Quality of Life (QoL), cost-effectiveness of reactive versus adaptive dosing of IRD and cost-effectiveness of R+I versus R.

Exploratory outcomes

Exploratory outcomes are to prospectively validate the UK-MRA Myeloma Risk Profile, to assess the usefulness of the Karnofsky performance status (PS), and consider the association of molecular subgroups with response, PFS and OS.

Participants Timelines

The full trial schema can be seen in Figure 3. The schedule of assessments at each time point are presented in Figure 4.

Trial Entry

Participants will enter the trial at one of two points in their patient pathway, this will either at bone marrow registration or main trial registration.

Trial consent

Participants who enter the trial at bone marrow registration will provide consent to having bone marrow and blood samples taken and sent to central laboratories for analysis. If the participant is diagnosed with a plasma cell dyscrasia, other than myeloma, or they have myeloma, but decide not to take part in the FiTNEss trial, they will also have the option of consenting to their samples being used in future research.

All participants, will provide written informed consent for the trial prior to trial registration. Optional consent for QoL resources and the use of samples for future research will also be obtained.

[3] Flow MRD will be assessed only. Additional detail on the testing of MRD and time points is presented in the 'Central Lab analysis' section of the supplementary material

For peer review only - http://bmjopen.bmj.com/site/about/guidelines.xhtml

Trial Registration

Following trial consent participants will be registered onto the main trial and assessed for eligibility. Consenting patients will complete the baseline QoL and Healthcare resource use questionnaires. Trial samples for blood and urine will be taken for all participants and bone marrow samples will be taken for those who did not enter the trial through bone marrow registration.

Trial Treatment

Following trial registration participants will be randomised into R1 and treated as described in the intervention schedule on a monthly basis. In absence of disease progression or intolerance, those participants with at least an MR following 12 cycles of maintenance and whom meet all of the R2 eligibility criteria will proceed to R2 maintenance treatment.

Participants will be followed up monthly (at each cycle) whilst receiving maintenance treatment, until death or until the final analysis of the trial (whichever happens sooner).

Trial Follow-up

Participants who discontinue treatment during induction and before the point of R2 will be followed up to the point of assessment for eligibility for R2, unless they withdraw consent for this. Thereafter, participants will continue to be followed-up for data pertaining to safety, progression (including second progression), and survival. Frailty scores will be completed for all participants at 2, 4, 6 and 12 months post-R1, irrespective of whether they have discontinued treatment. Similarly, QoL and Healthcare resources use questionnaires (if the participant has consented to these) will continue to be completed at 2, 6 and 12 months post-R1.

If treatment has been stopped without progression following R2, e.g. due to toxicity, then participants will be followed up two monthly until disease progression. Follow-up will include local investigations, central investigations, frailty index at 6 and 12 months post-R2 and QoL and Healthcare resource use at 6 and 12 months post-R2 (if consented).

Sample Size

In total 740 participants will be enrolled into the trial at R1 to ensure that at least 478 participants remain on trial and are randomised to R2. It is assumed that 65% of those randomised at R1 will be progression-free and, therefore, eligible for R2, hence 740 participants are required to be enrolled.

Based on data from the Myeloma XI non-intensive pathway we hypothesise the frail and unfit patients in R1 will be similar to the older patients in Myeloma XI (>75 years) who have an early treatment cessation rate (within 60 days of R1) of 20%. This hypothesis is based on the expectation that the frailty score is heavily driven by patient age. Younger patients (≤75 years) in the Myeloma XI non-intensive pathway have an early treatment cessation rate of 9% and it is our hypothesis that our frailty-based dosing schedule has the potential to reduce the rate amongst the unfit and frail patients to the proportion observed in fit patients [28 29] .

To demonstrate a decrease of 11 percent points in the proportion of early treatment cessation from 20% in the standard dosing schedule arm to 9% in the frailty score-adjusted dose arm amongst those patients scored at baseline to be unfit or frail would require the recruitment of 324 patients with an allocation ratio of 1:1. These calculations are based on a Pearson's chi-squared test without continuity correction, assume a 2-sided 5% level of significance, 80% power, and allow for a 1% dropout prior to 60 days post-randomisation. Given that we anticipate that 45% of patients will be scored as unfit or frail, by assuming this trial will have a similar underlying population as in Myeloma XI non-intensive

For peer review only - http://bmjopen.bmj.com/site/about/guidelines.xhtml

pathway and the age distributions in the IMWG report proposing the frailty score [14], we would anticipate that we will require 720 patients to enter the trial at R1 to have sufficient unfit and frail patients available to answer this question.

For R2, in the non-intensive pathway of Myeloma XI, the median PFS for patients on R following CRDa induction was approximately 33 months [19], where approximately 65% of individuals were progression-free 12 months post-randomisation. As R2 is approximately 12 months following R1 in FiTNEss we assume that the median PFS for patients receiving R maintenance therapy will be 21 months from R2. Tourmaline-MM1 [12] demonstrated a hazard ratio for PFS of 0.74 when comparing IRD and RD in patients with relapsed and refractory multiple myeloma. Thus a similar hazard ratio would be the minimum clinically relevant difference for our comparison in patients with newly diagnosed multiple myeloma. Assuming a median PFS of 29 months for those in the R+I maintenance group in addition to the assumption of 21 months in the R group equates to a hazard ratio of 0.72.

The above assumptions require the recruitment of 478 participants over a 30 month recruitment period with a further 24 months of follow-up. These calculations also assume a 2-sided 5% significance level, 80% power and allow for a 3% dropout rate prior to a PFS event being experienced. Note that 80% power is attained when 302 events have been observed. A total of 740 participants should be allocated to R1 to ensure that 478 participants are available at the second randomisation.

Overall survival is considered to be a key secondary endpoint for R2, 180 events with a minimum of 2 years follow up for all participants are required for 80% power.

Recruitment

It is planned that 740 participants will be recruited over a 30 month recruitment period from 87 UK centres. Once all centres are open, the recruitment target is 30 participants a month. The trial opened to recruitment on the 4th August 2020. As of May 2021 85 sites are open to recruitment and 137 participants have been randomised to the first stage of the trial.

In order to ensure the trial will meet the target sample size within the recruitment period, site set-up was prioritised whilst the trial was preparing to open to recruitment. In addition, the trial team are actively engaging with Principal Investigators at sites who support the trial to ensure that those sites open quickly and are maintaining regular communication with open sites to ensure that they continue to recruit to the trial. Finally, whilst the trial was originally delayed due to the COVID-19 pandemic, efforts were made to ensure that it opened as soon as possible when research restarted. One factor being the positive risk to benefit ratio of the number of hospital visits required for the trial interventions as compared to standard of care.

Assignment of treatment allocations

Each of the registration and randomisation procedures will be conducted centrally using the Leeds CTRU (Clinical Trial Research Unit) automated 24 hour web based and telephone system.

Registrations

If a patient is suspected to have myeloma they will enter the trial at the time of routine diagnostic tests, through bone marrow registration. Once diagnosis is confirmed locally and the research team consider the patient potentially eligible for the trial, patients will be provided with full verbal explanation of the trial and the full participant information sheet and informed consent documents to consider. Once the participant has provided informed consent, they will be registered onto the trial.

For peer review only - http://bmjopen.bmj.com/site/about/guidelines.xhtml

Other participants who have myeloma confirmed prior to entering trial, will enter at trial registration.

Randomisations

Following trial registration patients will be assessed for R1 eligibility. Eligible participants will be randomised on a 1:1 basis into R1, using the stratification factors; centre, IMWG frailty category, Beta-2 microglobulin concentration (<3.5, 3.5-<5.5, ≥5.5 mg/L), Haemaglobulin concentration (<100, $\geq 100g/L$), serum crestinine concentration (<175, ≥ 175 µmol/L), corrected serum calcium concentration (<2.75, ≥ 2.75mmol/L), platelets (<150, ≥ 150 x $10^9/L$).

Following R1, and as soon as the end of induction response is known, R2 eligible participants will be randomised on a 1:1 basis into R2, using the stratification factors; centre, allocated induction arm (reactive, adaptive); final response to induction treatment (<VGPR, ≥ VGPR).

For both R1 and R2, a computer generated minimization program that incorporates a random element will be used to ensure treatment groups are well balanced for the specified stratification factors.

Blinding Methods

For R2 treatment allocation will be concealed from participants, treatment provider and the trial team. The placebo and ixazomib capsules will be identical in colour, size, packaging and labelling.

To maintain the overall integrity of the trial design, unblinding will only be permitted in exceptional circumstances; for example valid medical or safety reasons where assuming that the patient is receiving active treatment and/ or stopping the blinded medication is insufficient.

Unblinding will be conducted automatically using an online system accessed by an authorised member of the site research team.

If unblinding is performed at any stage during the trial, decisions around further trial treatment will be the responsibility of the principal investigator or delegate. In either case unblinded participants will be followed up as per the protocol.

At the completion of the trial and after final analysis, participants will be given the opportunity to be informed of their allocation by their research site.

Data Collection

Clinical data will be collected both electronically and on paper by staff at research site completing CRFs provided by CTRU. QoL and Healthcare Resource Use questionnaires will be completed on paper CRFs by the participant. All paper CRFs will be sent to CTRU, by the research site, usually via standard post and entered onto an electronic database. This data along with the data entered electronically by staff at each research sites will be validated and monitored for completeness and quality by the CTRU.

Missing data will be chased until it is received, confirmed as not available or the trial is at analysis. Missing QoL data items will not be chased from participants, although missing questionnaires may be chased from sites.

Data management

Validation checks will be incorporated into the trial database to verify the data, and discrepancy reports will be generated for resolution by the trial site. Priority validations will be incorporated to ensure that any discrepancies related to participant rights, or the safety of participants, are expedited to sites for resolution. The CTRU/Sponsor will reserve the right to intermittently conduct source data

For peer review only - http://bmjopen.bmj.com/site/about/guidelines.xhtml

verification exercises on a sample of participants, which will be carried out by staff from the CTRU/Sponsor. Source data verification will involve direct access to participant notes at the participating hospital sites and the ongoing central collection of copies of consent forms and other relevant investigation reports.

Statistical methods

Statistical analysis is the responsibility of the CTRU statisticians, with the exception of the analysis for cost-effectiveness of delivery of IRD and R/R+I, which will be undertaken by Health Economists at the University of Leeds. A full statistical analysis plan (SAP) and Health Economics Analysis Plan (HEAP) will be written and approved before any analysis is undertaken.

All analyses will be conducted on the intention-to-treat (ITT) population, where participants will be included according to their randomisation allocation regardless of eligibility, whether they prematurely discontinued treatment, or did not comply with the regimen. A per-protocol (PP) analysis, where participants will be included if they received their allocated intervention according to the protocol, will be considered for the primary endpoints if there are a considerable number of major protocol violators. The safety population will consist of all participants who received at least one dose of the trial treatment and participants will be summarised as per their treatment received rather than their allocation.

An overall two-sided 5% significance level will be used for all efficacy endpoint comparisons. For the primary endpoints, this will be adjusted to account for the formal interim analyses.

Primary Endpoint Analysis

<u>Randomisation 1</u>

For R1, the number and proportion of participants, categorised as unfit or frail at baseline, experiencing an early treatment cessation event will be summarised by randomisation allocation and exact 95% confidence intervals will be calculated.

A logistic regression model will regress early treatment cessation on randomisation allocation (reactive / adaptive dosing) adjusting for the stratification factors of the trial. A statistically significant induction treatment effect will be suggested if the p-value for the resulting odds ratio is <0.047. Parameter estimates, odds ratios and corresponding 95% confidence intervals, degrees of freedom, test statistics and p-values will be presented for each variable in the model. Residuals and predicted values produced from the models will be examined to assess the assumptions of the statistical models.

<u>Randomisation 2</u>

For R2, PFS between the two maintenance therapies (R+Placebo / R+I) will be compared using a Cox regression model adjusting for the stratification factors of the trial. A statistically significant maintenance treatment effect will be suggested if the p-value for the hazard ratio corresponding to randomisation allocation is <0.047. Parameter estimates, hazard ratios and corresponding 95% confidence intervals, degrees of freedom, test statistics and p-values will be presented for each variable in the model.

The proportional hazards assumptions will be assessed by plotting the hazards over time (i.e. the log cumulative hazard plot) for each treatment arm and using appropriate statistical tests. If evidence is

For peer review only - http://bmjopen.bmj.com/site/about/guidelines.xhtml

found to support the violation of the proportional hazards assumption then alternative appropriate analysis methods will be investigated.

No imputation strategy is planned for the primary endpoints.

Secondary Endpoint Analysis

Secondary endpoint analysis of OS and other time-to-event endpoints will be analysed using similar methods to those described for PFS.

MRD negativity and other binary endpoints will be analysed using similar methods to those described for the early treatment cessation primary endpoint. The number and proportion of participants in each response category (sCR, CR, VGPR, etc.) will be summarised by allocated treatment and exact 95% confidence intervals will be calculated. The difference in proportions for each response category will be presented with corresponding 95% confidence intervals.

The domains of the QoL questionnaires will be summarised using mean scores adjusted for baseline and 95% CIs at each assessment time-point. Similar summaries will be produced for quality-adjusted life years (QALYs) derived using the EQ-5D-3L questionnaire.

Exploratory and subgroup analyses

An overview of the planned exploratory and subgroup analysis can be found in the supplementary material. These include genetic and molecular analysis of patient samples conducted by the respective central laboratories.

Health economics

A full HEAP will be written and approved before any analysis is undertaken.

Economic evaluations will be conducted at R1 and R2, using within-trial and decision-model based analyses. The analysis will be guided by the NICE reference case, applying the cost-utility framework from the perspective of the health and social care provider over a life-time horizon. Base case QALYs will be based on EQ-5D-3L responses, and costs on patient completed resource use forms and hospital records. Results will be presented in terms of incremental cost-effectiveness ratios, cost-effectiveness acceptability frontiers and net benefit.

Trial Oversight

The trial management group (TMG), comprises of the Chief and Co-Chief Investigators, CTRU team and co-investigators and are responsible for the clinical set up, ongoing management, promotion of the trial and for the interpretation of the results. The trial steering committee (TSC), consisting of independent clinicians and statisticians, along with a patient representative, will provide overall supervision on the trial, including trial progress, adherence to protocol, participant safety and consideration of new information.

Data monitoring

An independent data monitoring and ethics committee (DMEC) will review the safety and ethics of the trial by reviewing unblinded interim data prepared by the CTRU in strict confidence at approximately yearly intervals. Unblinded safety updates are also prepared at 6 monthly intervals.

For peer review only - http://bmjopen.bmj.com/site/about/guidelines.xhtml

After each annual review of safety data, the DMEC will make their recommendations to the TSC about the continuation of the trial who will make their recommendations known to the TMG.

Interim Analyses

Two formal interim analyses will be undertaken for early efficacy, one for each of the randomisations. The first will occur when 50% of required participants (370 participants) have reached 60 days post-R1. The second will occur when 50% of required PFS events have been observed (151 events) following R2. In order to maintain an overall two-sided 5% significance level for the primary endpoint analysis the O'Brien and Fleming alpha spending function [30] will be used. This results in a 0.05% significance level for the interim analysis. The analysis itself will reflect that detailed in the statistical analysis section. For the second interim analysis, only the DMEC, safety statistician and supervising statistician will see the unblinded results, as is standard procedure for double blind trials.

No other formal analysis of the trial is planned before the participants have attained the primary endpoints.

The DMEC, in the light of the interim data, and any advice or evidence they wish to request, will advise the TSC if there is proof beyond reasonable doubt that one treatment is better and recommend appropriate changes to the trial protocol.

Harms

Adverse Events

Adverse Events (AEs) are any untoward medical occurrence in a patient or clinical trial subject administrated a medicinal product and which does not necessarily have a causal relationship with this treatment. AEs can be defined as any unfavourable and unintended sign (including an abnormal laboratory finding, for example), symptom or disease. Due to the nature of myeloma and its treatment, participants are likely to experience several AEs throughout the course of the disease.

All AEs, both related and unrelated to myeloma treatment will be collected on the relevant CRF from R1 until 60 days after the last dose of protocol treatment and will be evaluated and summarised in accordance with the NCI-CTCAE V5.

Serious Adverse Events

Serious Adverse Events (SAEs) are defined as any untoward medical occurrences or effects that at any dose result in death; or are life threatening (at the time of event); or require in patient hospitalisation or prolongation of existing hospitalisation; or result in persistent or significant disability of incapacity; or result in a congenital abnormality or birth defect; or jeopardise the participant or may require an intervention to prevent one of the above outcomes/consequences (other important medical event). SAEs will be reported from R1 until 60 days post the last dose of trial drug.

Serious Adverse Reactions (SARs) are SAEs that are deemed to be possibly related to any dose administrated of any trial treatment. Suspected unexpected serious adverse reactions (SUSARs) are SARs, of which the nature and severity is not consistent with the applicable reference safety information. SUSARs and SARs will be reported from the date of the first trial drug for the duration of the trial.

For peer review only - http://bmjopen.bmj.com/site/about/guidelines.xhtml

Presenting Safety Data

Safety analyses will summarise all SUSARs, SARs, SAEs, ARs, AEs and treatment-related mortality rates. Safety data will be presented by treatment group for the safety population in addition to suspected relationship to trial treatment.

Secondary Primary Malignancies

All new secondary primary malignancies (SPMs) or suspected malignancies will be recorded from R1 for the duration of the trial and will be summarised and reviewed by an appointed member of the TMG, who will determine whether trial treatment should continue.

Pregnancies

The Celgene approved pregnancy programme will be followed as per usual clinical practice. Pregnancies in participants on trial treatment will be prevented as effectively as possible. Pregnancies and suspected pregnancies in a female or male participant's partner occurring at any time until 90 days post cessation of trial treatment will be reported.

Auditing

The CTRU and the trial Sponsor have procedures in place to ensure that serious breaches of GCP or the trial protocol are identified and reported. A triggered monitoring plan will ensure that sites at risk are monitored accordingly.

Patient and public involvement

FiTNEss has been developed following extensive discussion within the UK myeloma community, including with the NCRI Myeloma Subgroup (the UK Myeloma Research Alliance (UK-MRA)) and the NCRI Haematological Oncology Group. Both groups include patient and public representatives who work with clinical members of the group. To develop studies which address key questions for induction and maintenance.The protocol was reviewed in depth by a patient representative in order to ensure that the interventions and proposed schedule of assessments would be acceptable to patients. In addition, the trial consent and participant information document was reviewed for clarity by the same patient representative. Furthermore, to ensure that the patient perspective is considered throughout the trial, a patient representative sits on the TSC.

Ethics and Dissemination

Ethics approval statement

Ethical approval has been obtained from the North East - Tyne & Wear South Research Ethics Committee (reference 19/NE/0125). In addition approval was granted by the appropriate local research and development department for each participating centre prior to opening to recruitment. Participants will be required to provide written informed consent before joining the trial.

Protocol Amendments

The trial opened to recruitment on 4th August 2020 using protocol version 2, dated 10th October 2019. An amendment to protocol version 3 is anticipated in June 2021, which will include the addition of the secondary endpoint Event-Free Survival, clarification on the requirements around when face-to-face assessments should be conducted, and adding in the recommendation of 5 mg once daily of lenalidomide for participants with severe renal impairment, as opposed to 15mg every other day.

For peer review only - http://bmjopen.bmj.com/site/about/guidelines.xhtml

Consent

The Principal Investigator retains overall responsibility for the informed consent of participants at their site and must ensure that any medically qualified person delegated responsibility to participate in the informed consent process is duly authorized, trained and competent to participate according to ethical approved protocol, principles of Good Clinical Practice (GCP) and Declaration of Helsinki. Written consent will be obtained and signed by a medically qualified member of the site research team. A record of the consent process for both bone marrow and blood sample consent and full trial consent, including the date of consent and all those present, will be kept in the participant's notes. At any stage, participants can withdraw consent without repercussion.

Confidentiality

All information collected during the course of the trial will be kept strictly confidential. Information will be held securely on paper at Leeds CTRU. In addition, the CTRU will hold electronic information on all trial participants. The CTRU will have access to the entire database for monitoring, coordination, and analysis purposes.

Access to data

Data will not be made available until the end of the study, The CTRU will control the final trial datasets, and any requests for data will be reviewed by the trial management group in the first instance. Only requests that have a methodologically sound proposal and whose proposed use of the data has been approved by the independent trial steering committee, based on the principles of a controlled access approach, and subject to existing contractual agreements with the funder, will be considered. Proposals should be directed to Leeds Clinical Trials unit *(*CTRU-DataAccess@leeds.ac.uk) in the first instance; to gain access, data requestors will need to sign a data access agreement.

Ancillary and post-trial care

Participants who stop trial treatment due to progression or any point prior to the end of trial will be treated off-trial at the discretion of their treating clinician. Following disease progression, participants will be followed up annually until death, or until the end of the trial for post-progression endpoints.

Dissemination policy

Authorship of clinical and translational outputs will be in keeping with the UK-MRA Publication Policy and due acknowledgement to participants, local investigators, funders and NCRI Haematological-Oncology Group support made. The success of the trial depends upon the collaboration of all trial members. For this reason, credit for the main results will be given to all those who have collaborated in the trial, through authorship and contributorship. Uniform requirements for authorship for manuscripts submitted to medical journals will guide authorship decisions alongside the guidance of the UK-MRA.

To maintain the scientific integrity of the trial, data will not be released prior to the end of the trial or a primary endpoint being reached, either for trial publication or oral presentation purposes, without the permission of the Trial Steering Committee and the (Co-)Chief Investigators. In addition, individual collaborators must not publish data concerning their participants that is directly relevant to the questions posed in the trial until the main results of the trial have been published and following written consent from the Sponsor.

Appendices

For peer review only - http://bmjopen.bmj.com/site/about/guidelines.xhtml

Informed consent material

The consent forms which are to be completed by the participant at Bone Marrow Registration and/or Trial Registration are included in supplementary material.

Biological specimens

The collection of central samples for laboratory analysis is summarised in Figure 4. The analysis to be conducted for trial purposes is stated in the supplementary material. Additional analysis may be carried out by each central laboratory provided the appropriate consent for sample use in future research has been provided by the participant at trial entry.

Author Contributions

The FiTNEss trial was conceived by GC and GJ and designed by them in collaboration with CPaw, DAC, AH, JB, SB, MK, RO, MJ, BK, and MD. All authors (ABC, KLR, CPaw, DAC, AH, JB, SB, MK, RT, NR, KB, JJ, CP, HG, DM, BD, CO, RH, PB, RO, MJ, BK, MD, GJ and GC) inputted into the development of the protocol and patient information sheet. The first draft of the manuscript was written by ABC and KLR. All authors (ABC, KLR, CPaw, DAC, AH, JB, SB, MK, RT, NR, KB, JJ, CP, HG, DM, BD, CO, RH, PB, RO, MJ, BK, MD, GJ and GC) reviewed and approved the final manuscript.

Funding

This trial is funded by Cancer Research UK (C37712/A21282) with further unrestricted funding from Millennium: The Takeda Oncology Company and Celgene: A Bristol Myers Squibb Company. This work was also supported by Core Clinical Trials Unit Infrastructure from Cancer Research UK (C7852/A25447). The funders had no role in the design, collection, analysis or collection of data; in writing the manuscript; or in the decision to submit the manuscript for publication.

The trial Sponsor is responsible for the overall conduct of the trial as defined by Directive 2001/20/EC is University of Leeds, UoL/LTHT Joint Sponsor QA office (CTIMPs); Research & Innovation Centre/Faculty of Medicine & Health; Leeds Teaching Hospitals NHS Trust/University of Leeds; St James University Hospital; Leeds LS9 7TF.

Competing Interest Statement

ABC, KLR, DAC, AH, CO, RH, PB report grants and non-financial support from BMS/Celgene, grants and non-financial support from Merck Sharpe & Dohme, grants and non-financial support from Amgen, grants and non-financial support from Takeda, during the conduct of the trial. DAC also reports travel support from Celgene Corporation. Cpaw reports receiving honoraria and/or travel support from Amgen, BMS/Celgene, Janssen, Sanofi and Takeda. MK consultancy: AbbVie, Amgen, BMS/Celgene, GSK, Janssen, Karyopharm, Seattle Genetics, Takeda; honoraria: BMS/Celgene, Janssen, Takeda; Research funding: BMS/Celgene; Travel/educational support: BMS/Celgene, Janssen, Takeda. SB reports receiving research funding from Takeda. KB – Advisory Boards Janssen: BMS/Celgene, Takeda, Novartis. Speaker Honoraria: Janssen, BMS/Celgene, Sanofi, Takeda. Support to attend educational meetings: Janssen, BMS/Celgene, Takeda, GSK.GJ reports research funding from Takeda, Onyx, MSD & BMS/Celgene with consultancy from Janssen, Takeda, Sanofi, Oncopeptides, Karyopharm, Pfizer, Roche & BMS/Celgene. GC reports research funding from Janssen, Takeda, Amgen & BMS/Celgene with consultancy from Janssen, Takeda, Sanofi, Oncopeptides, Karyopharm, Pfizer, Roche & BMS/Celgene .MD reports Stock held in Abingdon Health LtdJB, RDT, NR, JJ, CPar, HG, DM, BD, RO, MJ and BK have no declared competing interests.

For peer review only - http://bmjopen.bmj.com/site/about/guidelines.xhtml

For peer review only

For peer review only - http://bmjopen.bmj.com/site/about/guidelines.xhtml

References

1. CANCER RESEARCH UK. Myeloma Incidence Statistics [online]: CANCER RESEARCH UK; 2015 [Available from: http://www.cancerresearchuk.org/health-professional/cancer-statistics/statistics-by-cancer-type/myeloma/incidence accessed June.
2. Morgan GJ, Davies FE, Gregory WM, et al. Long-term Follow-up of MRC Myeloma IX Trial: Survival Outcomes with Bisphosphonate and Thalidomide Treatment. *Clinical cancer research* 2013;19(21):6030-38. doi: 10.1158/1078-0432.CCR-12-3211
3. Pawlyn C, Cairns D, Kaiser M, et al. The relative importance of factors predicting outcome for myeloma patients at different ages: results from 3894 patients in the Myeloma XI trial. 2020
4. Jackson GH, Davies FE, Pawlyn C, et al. Response-adapted intensification with cyclophosphamide, bortezomib, and dexamethasone versus no intensification in patients with newly diagnosed multiple myeloma (Myeloma XI): a multicentre, open-label, randomised, phase 3 trial. *The Lancet Haematology* 2019;6(12):e616-e29. doi: 10.1016/S2352-3026(19)30167-X
5. Pawlyn C, Davies FE, Cairns DA, et al. Quadruplet Vs Sequential Triplet Induction Therapy Approaches to Maximise Response for Newly Diagnosed, Transplant Eligible, Myeloma Patients. *Blood* 2015;126(189)
6. Jackson GH, Pawlyn C, Cairns DA, et al. Carfilzomib, lenalidomide, dexamethasone, and cyclophosphamide (KRdc) as induction therapy for transplant-eligible, newly diagnosed multiple myeloma patients (Myeloma XI+): Interim analysis of an open-label randomised controlled trial. *PLoS medicine* 2021;18(1):e1003454-e54. doi: 10.1371/journal.pmed.1003454
7. Richardson PG, Baz R, Wang M, et al. Phase 1 study of twice-weekly ixazomib, an oral proteasome inhibitor, in relapsed/refractory multiple myeloma patients. *Blood* 2014;124(7):1038-46. doi: 10.1182/blood-2014-01-548826
8. Offidani M, Corvatta L, Caraffa P, et al. An evidence-based review of ixazomib citrate and its potential in the treatment of newly diagnosed multiple myeloma. *OncoTargets and therapy* 2014;7:1793-800. doi: 10.2147/OTT.S49187
9. Assouline SE, Chang J, Cheson BD, et al. Phase 1 dose-escalation study of IV ixazomib, an investigational proteasome inhibitor, in patients with relapsed/refractory lymphoma. *Blood cancer journal (New York)* 2014;4(10):e251-e51. doi: 10.1038/bcj.2014.71
10. Gupta N, Zhao Y, Hui AM, et al. Switching from body surface area-based to fixed dosing for the investigational proteasome inhibitor ixazomib: a population pharmacokinetic analysis. *British journal of clinical pharmacology* 2015;79(5):789-800. doi: 10.1111/bcp.12542
11. Kumar SK, Berdeja JG, Niesvizky R, et al. Safety and tolerability of ixazomib, an oral proteasome inhibitor, in combination with lenalidomide and dexamethasone in patients with previously untreated multiple myeloma: an open-label phase 1/2 study. *The Lancet Oncology* 2014;15(13):1503-12.
12. Moreau P, Masszi T, Grzasko N, et al. Ixazomib, an investigational oral proteasome inhibitor (PI), in combination with lenalidomide and dexamethasone (IRd), significantly extends progression-free survival (PFS) for patients (Pts) with relapsed and/or refractory multiple myeloma (RRMM): the phase 3 Tourmaline-MM1 study (NCT01564537): Am Soc Hematology, 2015.
13. Facon T, Venner CP, Bahlis NJ, et al. MM-347: Ixazomib Plus Lenalidomide-Dexamethasone (IRd) vs. Placebo-Rd for Newly Diagnosed Multiple Myeloma (NDMM) Patients Not Eligible for Autologous Stem Cell Transplant: The Double-Blind, Placebo-Controlled, Phase 3 TOURMALINE-MM2 Trial. *Clinical lymphoma, myeloma and leukemia* 2020;20:S307-S08. doi: 10.1016/S2152-2650(20)30955-1
14. Palumbo A, Bringhen S, Mateos M-V, et al. Geriatric assessment predicts survival and toxicities in elderly myeloma patients: an International Myeloma Working Group report. *Blood* 2015;125(13):2068-74.

For peer review only - http://bmjopen.bmj.com/site/about/guidelines.xhtml

15. Katz S, Ford AB, Moskowitz RW, et al. Studies of illness in the aged: The index of adl: a standardized measure of biological and psychosocial function. *JAMA* 1963;185(12):914-19. doi: 10.1001/jama.1963.03060120024016
16. Lawton MP, Brody EM. Assessment of older people: self-maintaining and instrumental activities of daily living. *The gerontologist* 1969;9(3_Part_1):179-86.
17. Charlson ME, Pompei P, Ales KL, et al. A new method of classifying prognostic comorbidity in longitudinal studies: development and validation. *Journal of chronic diseases* 1987;40(5):373-83.
18. Delforge M, Minuk L, Eisenmann J-C, et al. Health-related quality-of-life in patients with newly diagnosed multiple myeloma in the FIRST trial: lenalidomide plus low-dose dexamethasone versus melphalan, prednisone, thalidomide. *Haematologica* 2015;100(6):826-33. doi: 10.3324/haematol.2014.120121
19. Jackson GH, Davies FE, Pawlyn C, et al. Lenalidomide Is a Highly Effective Maintenance Therapy in Myeloma Patients of All Ages; Results of the Phase III Myeloma XI Study. *Blood* 2016;128(22):1143-43.
20. Jackson G, Davies FE, Pawlyn C, et al. Lenalidomide Maintenance Significantly Improves Outcomes Compared to Observation Irrespective of Cytogenetic Risk: Results of the Myeloma XI Trial. *Blood* 2017;130(Supplement 1):436-36. doi: 10.1182/blood.V130.Suppl_1.436.436 %J Blood
21. Kumar S, Berdeja JG, Niesvizky R, et al. Long-Term Ixazomib Maintenance Is Tolerable and Improves Depth of Response Following Ixazomib-Lenalidomide-Dexamethasone Induction in Patients (Pts) with Previously Untreated Multiple Myeloma (MM): Phase 2 Study Results. *Blood* 2014;124(21):82-82.
22. Dimopoulos MA, Spicka I, Quach H, et al. Ixazomib as Postinduction Maintenance for Patients With Newly Diagnosed Multiple Myeloma Not Undergoing Autologous Stem Cell Transplantation: The Phase III TOURMALINE-MM4 Trial. *Journal of clinical oncology* 2020;38(34):4030-41. doi: 10.1200/JCO.20.02060
23. Chan A-W, Tetzlaff JM, Altman DG, et al. SPIRIT 2013 Statement: Defining Standard Protocol Items for Clinical Trials. *Annals of internal medicine* 2013;158(3):200-07. doi: 10.7326/0003-4819-158-3-201302050-00583
24. Chan A-W, Tetzlaff JM, Gøtzsche PC, et al. SPIRIT 2013 explanation and elaboration: guidance for protocols of clinical trials. *BMJ (Online)* 2013;346(jan08 15):907-e7586. doi: 10.1136/bmj.e7586
25. The International Myeloma Working Group (IMWG). updated criteria for the diagnosis of multiple myeloma. *The Lancet 2014*;15 (12):538-48 (43).
26. Rajkumar SV, Harousseau JL, Durie B, et al. Consensus recommendations for the uniform reporting of clinical trials: report of the International Myeloma Workshop Consensus Panel 1. *Blood* 2011;117(18):4691-5. doi: 10.1182/blood-2010-10-299487
27. Kumar S, Paiva B, Anderson KC, et al. International Myeloma Working Group consensus criteria for response and minimal residual disease assessment in multiple myeloma. *The Lancet Oncology* 2016;17(8):e328-46. doi: 10.1016/S1470-2045(16)30206-6
28. Pawlyn C, Davies, F.E., Cairns, D., Striha, A., Best, P., Sigsworth, R., Jones, J.R., Kishore, B., Garg, M., Williams, C. and Karunanithi, K.,. Continuous Treatment with Lenalidomide Improves Outcomes in Newly Diagnosed Myeloma Patients Not Eligible for Autologous Stem Cell Transplant: Results of the Myeloma XI Trial. *ASH 2017* 2017
29. Jackson GH, Pawlyn C, Cairns DA, et al. Optimising the value of immunomodulatory drugs during induction and maintenance in transplant ineligible patients with newly diagnosed multiple myeloma: results from Myeloma XI, a multicentre, open-label, randomised, Phase III trial. *Br J Haematol* 2020 doi: 10.1111/bjh.16945 [published Online First: 2020/07/14]
30. O'Brien PC, Fleming TR. Multiple Testing Procedure for Clinical-Trials. *Biometrics* 1979;35(3):549-56. doi: Doi 10.2307/2530245

For peer review only - http://bmjopen.bmj.com/site/about/guidelines.xhtml

Figure Legends/ Captions

Figure 1A & Figure 1B: Overall survival in MRC-IX and NCRI-XI TE pathway (Figure 1A). Overall survival in MRC-IX and NCRI-XI TNE pathway (Figure 1B)

Figure 2: Reasons for ceasing induction treatment in NCRF=XI (n=928)

Figure 3: Flow Diagram of Myeloma XIV (FiTNEss) Trial

Figure 4: Summary of Investigations (Local and Central)

For peer review only

(A)

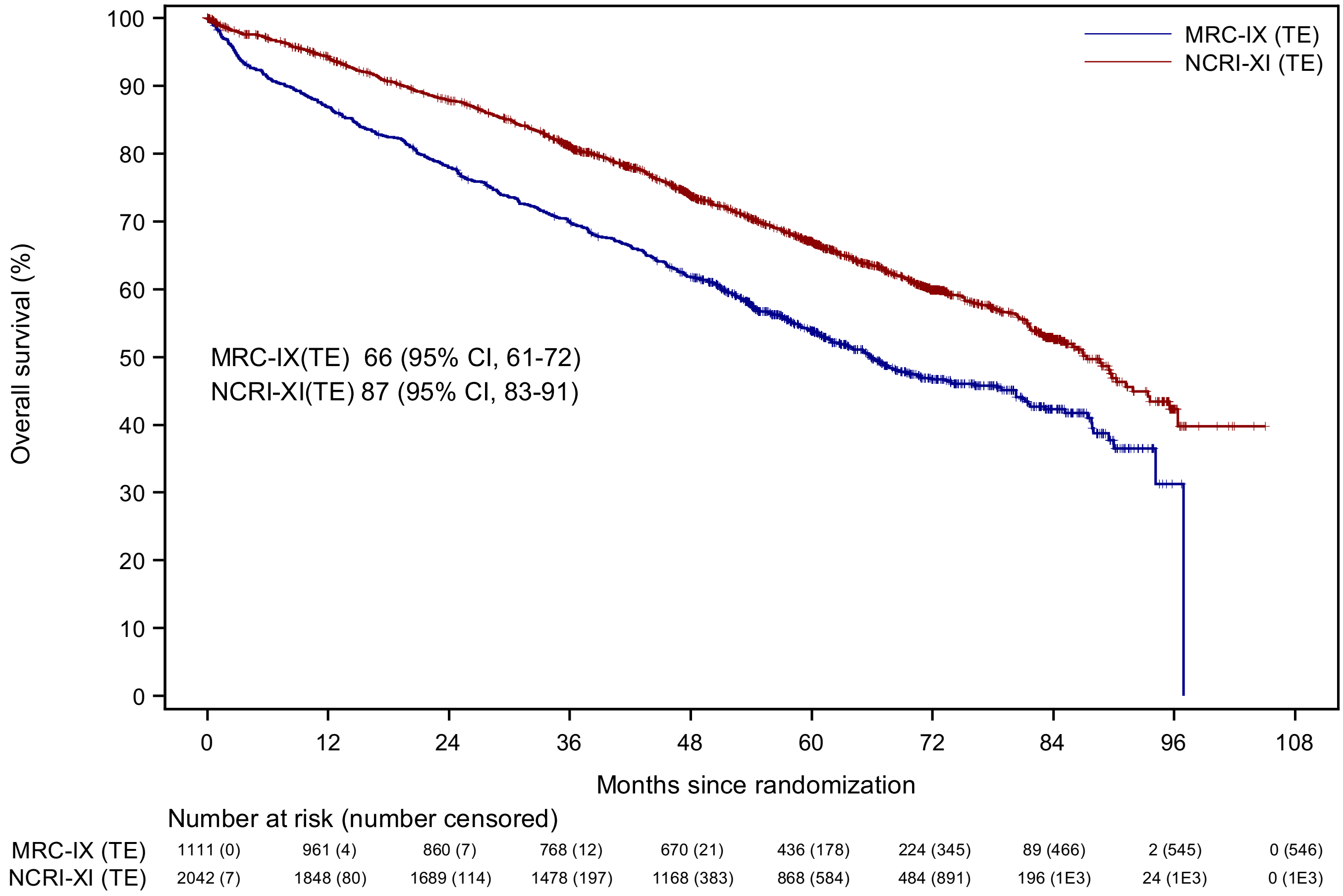

MRC-IX (TE)
NCRI-XI (TE)
Overall survival (%)
MRC-IX(TE) 66 (95% CI, 61-72)
NCRI-XI(TE) 87 (95% CI, 83-91)
Months since randomization
Number at risk (number censored)
MRC-IX (TE) 1111 (0) 961 (4) 860 (7) 768 (12) 670 (21) 436 (178) 224 (345) 89 (466) 2 (545) 0 (546)
NCRI-XI (TE) 2042 (7) 1848 (80) 1689 (114) 1478 (197) 1168 (383) 868 (584) 484 (891) 196 (1E3) 24 (1E3) 0 (1E3)

(B)

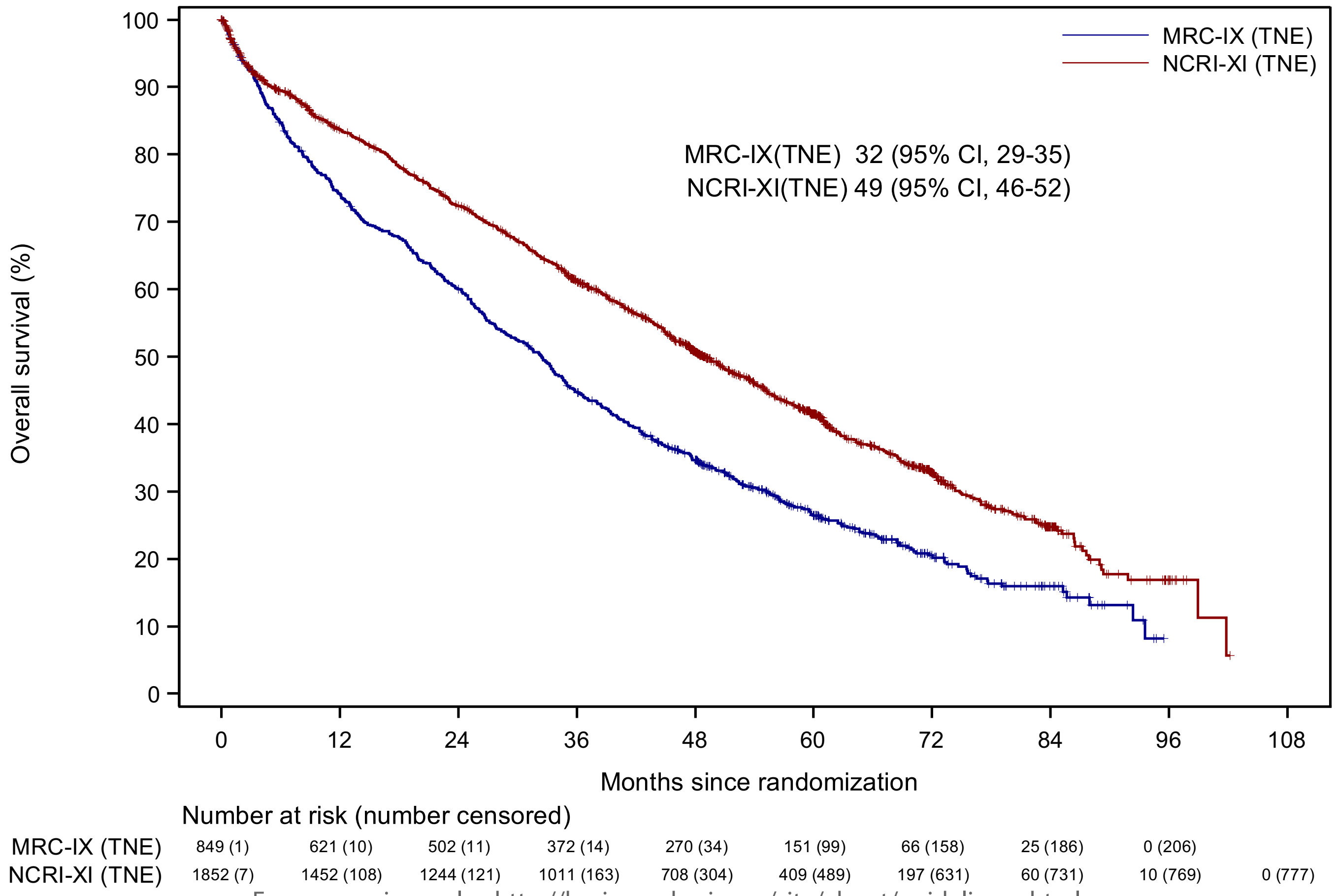

MRC-IX (TNE)
NCRI-XI (TNE)
Overall survival (%)
MRC-IX(TNE) 32 (95% CI, 29-35)
NCRI-XI(TNE) 49 (95% CI, 46-52)
Months since randomization
Number at risk (number censored)
MRC-IX (TNE) 849 (1) 621 (10) 502 (11) 372 (14) 270 (34) 151 (99) 66 (158) 25 (186) 0 (206)
NCRI-XI (TNE) 1852 (7) 1452 (108) 1244 (121) 1011 (163) 708 (304) 409 (489) 197 (631) 60 (731) 10 (769) 0 (777)

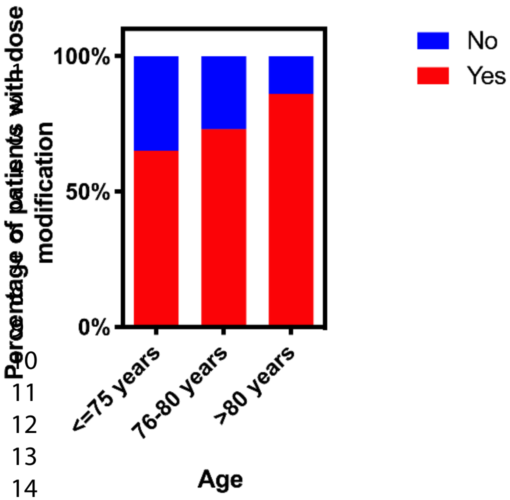
Percentage of patients with dose modification
100%
50%
0%
<=75 years
76-80 years
>80 years
Age
No
Yes

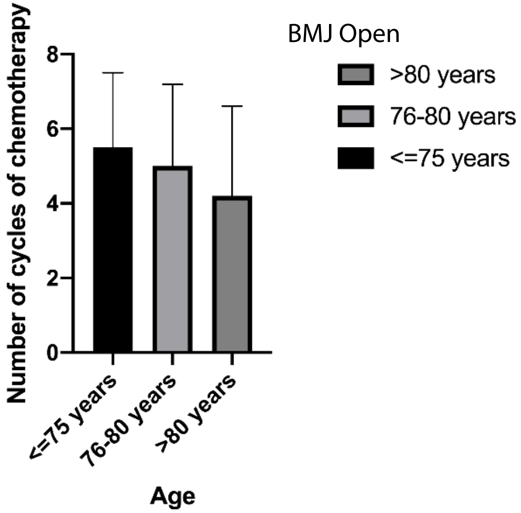
Number of cycles of chemotherapy
8
6
4
2
0
<=75 years
76-80 years
>80 years
Age
>80 years
76-80 years
<=75 years

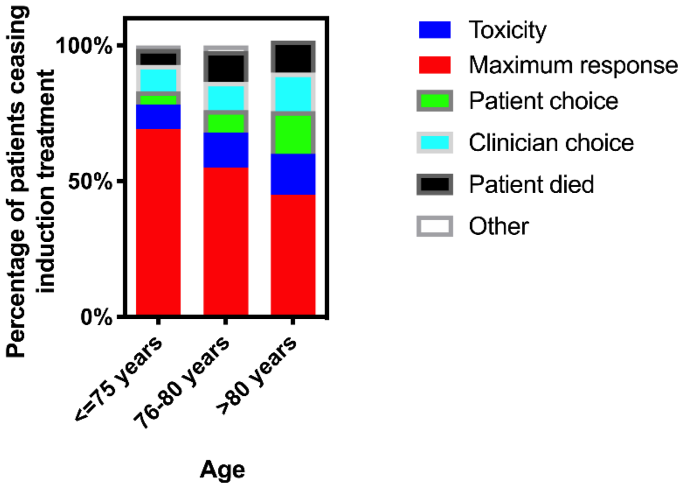
Percentage of patients ceasing induction treatment
100%
50%
0%
<=75 years
76-80 years
>80 years
Age
Toxicity
Maximum response
Patient choice
Clinician choice
Patient died
Other

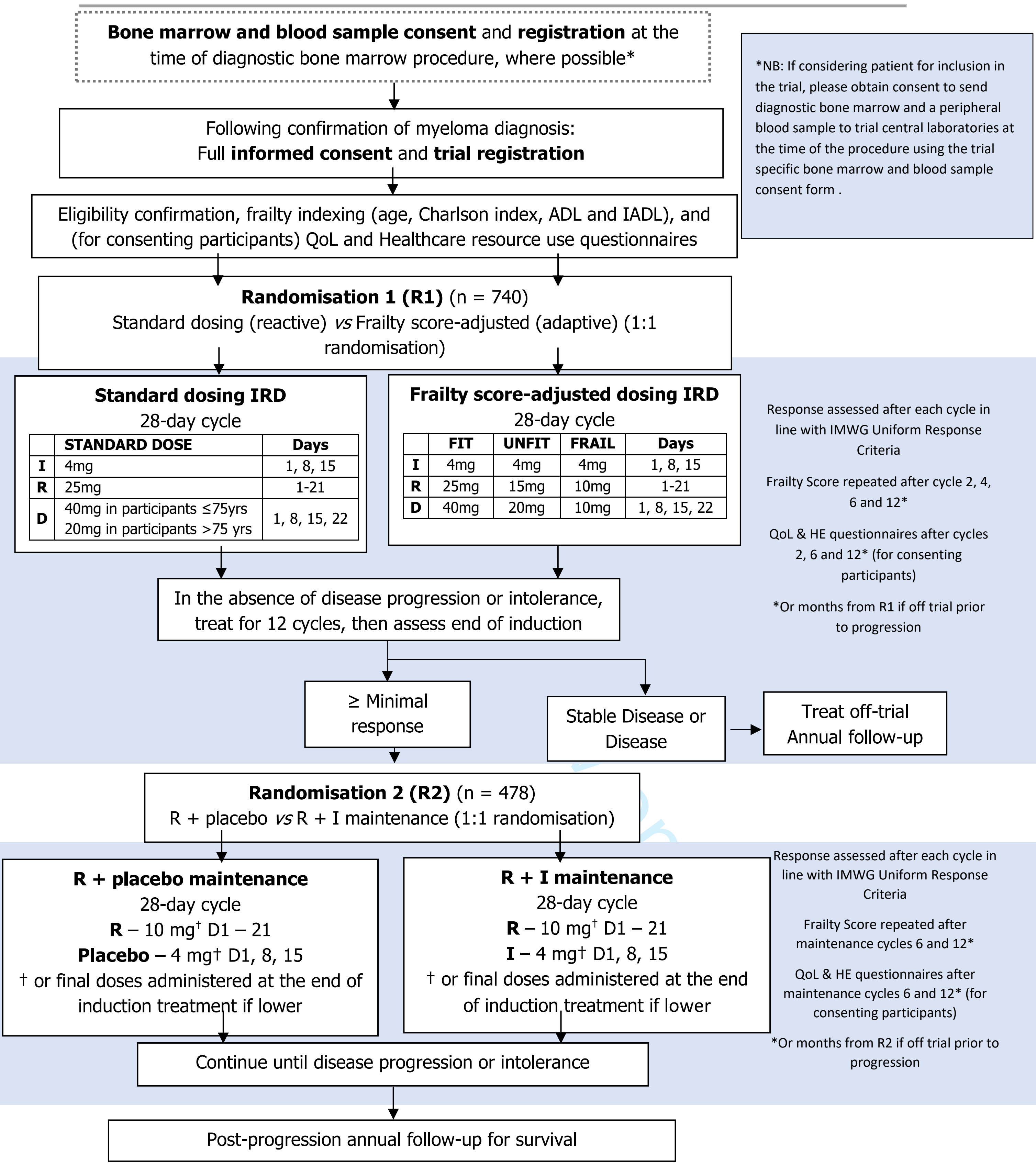

Figure 3: Flow Diagram of the trial

Figure 4: Schedule of assessments

Local Investigations	Consent & trial registration	Pre-R1 (assessments for eligibility within 14 days prior to R1, unless otherwise stated)	Day 1 (or ≤3 days prior) of each induction treatment cycle	End of 12 cycles of IRD induction treatment	Pre-Randomisation 2 (R2) (assessments for eligibility within 14 days prior to R2, do not need repeating if end of IRD assessments are within this timeframe)	Day 1 (or ≤3 days prior) of each maintenance treatment cycle	Disease Progression
Pre-trial Consent for Bone Marrow (at time of diagnostic bone marrow) and bone marrow registration	✓						
Full written informed consent and (main) trial registration	✓						
Medical history (including comorbidities, concomitant medications and previous malignancies)		✓					
Assessment of cardiac and thyroid function (as part of standard care)		✓	Monitor throughout treatment				
Physical examination (including height/weight, BP, performance status [f], vital signs [baseline only])		✓	✓	✓	✓	✓	✓
Pregnancy test (if female of child bearing potential, see Appendix 8)		✓	✓	✓	✓	✓[b]	
FBC, U&Es, calcium, creatinine, LFTs (including bilirubin, and AST and/or ALT), albumin, LDH, calculated creatinine clearance, Urinary protein:creatinine ratio* **only requested at baseline*		✓	✓[a]	✓	✓	✓	✓
CRP, β2M		✓	✓ After cycle 2, 4, and 6 [d]	✓		✓ After cycles 6 and 12 [c]	✓
Serum paraprotein, serum free light chains, serum total (class-specific) immunoglobulins and urinary light chain detection (quantification where available)		✓	✓ (To assess response to previous cycle, not applicable for C1)	✓	✓	✓ (To assess response to previous cycle)	✓
Bone marrow aspirate and (if available) trephine		✓ (Within 6 weeks prior to R1) [g]	If at any point a first occurrence of CR or sCR is suspected then bone marrow aspirate should be sent for local review as well as to HMDS, as detailed below. (s)CR cannot be confirmed without bone marrow.				✓
Serology of hepatitis B and C		✓					
Imaging		✓ (Within 3 months prior to randomisation)	Imaging of lytic and/or focal bone and extramedullary lesions if clinically indicated, in accordance with IMWG recommendations and local practice [e]				
IMWG Frailty Index (Charlson Comorbidity Score, IADL, ADL)		✓	✓ After cycle 2, 4, and 6 [d]	✓		✓ After cycles 6 and 12 [c]	
Quality of Life and Healthcare resource use questionnaires (EORTC QLQ-C30, QLQ-MY20, EQ-5D (3 Level)) **Completed by consenting participants in clinic*		✓ Before participant is informed of allocated dosing strategy	✓ After cycle 2 and 6 [d]	✓[d]		✓ After cycles 6 and 12 [c]	
Adverse Events			Monitor throughout study and report on relevant eCRFs (From randomisation until 60 days post last treatment dose)				
SAEs / SUSARs / SPMs			Monitor throughout study: all SUSARs & SAEs/SPMs must be reported to CTRU within 24 hours of the Site becoming aware of the event: refer to Section 14: (SAEs from randomisation until 60 days post last treatment dose & SPMs/SARs/SUSARs from first treatment dose until the end of trial)				

Central Analysis Investigations: Central Samples (all Participants – core consent)

Sample	Send to	Investigation	Bone marrow and blood sample consent samples <u>only</u>	Pre-randomisation 1 (pre-treatment) - Post Main Consent	During induction therapy	At the end of 12 cycles of IRD induction treatment (or at end of the final IRD cycle if sooner)	During maintenance therapy	Disease Progression
20 mL whole clotted blood or 10 mL serum	Birmingham	To confirm disease response & progression through investigation of paraprotein, immunoglobulins, serum free light chain, urinary free light chain and β2M		✓	✓ (After 2 cycles of IRD)	✓	✓ (At 2 monthly intervals)	✓
10 mL random urine				✓	✓ (After 2 cycles of IRD)	✓	✓ (At 2 monthly intervals)	✓
2 mL bone marrow aspirate in EDTA	HMDS, Leeds	To determine MRD	✓(Within 8 weeks prior to R1)	✓[¥]	✓ (After 6 cycles of IRD)	✓	✓ (At 12 months post-maintenance randomisation (R2))	✓
					If at any point a first occurrence of CR or sCR is suspected then bone marrow aspirate should be sent to HMDS.			
3 mL bone marrow aspirate in EDTA	ICR, London	Cytogenetic/Molecular research (including MLPA / FISH)	✓(Within 8 weeks prior to R1)	✓[*¥]				✓
6 mL peripheral blood in EDTA			✓(Within 8 weeks prior to R1)	✓[¥]				✓
20 mL peripheral blood in EDTA	LIMR, Leeds	Frailty biomarker studies		✓	✓ (After cycle 2, 4 & 6)	✓		✓
10 mL clotted blood				✓	✓ (After cycle 2, 4 & 6)	✓		✓
2 mL bone marrow aspirate in EDTA						✓ **	✓ (At 12 months post-maintenance randomisation (R2))	✓

a. The FBC should be repeated mid-cycle 1 (Day 14 +/-3 days) or more frequently and during subsequent cycles if there is a concern about cytopenias.
b. Pregnancy test must also be performed at 4 weeks after the end of study treatment.
c. Or at 6 and 12 months post-R2 if treatment is stopped prior to this for reasons other than disease progression.
d. Or at 2, 6 and 12 months post-R1 if treatment is stopped prior to this for reasons other than disease progression.
e. The imaging at baseline/pre-registration is mandatory. Subsequent imaging will be as per local protocols/standard of care - imaging will only need to be repeated if extramedullary disease was detected at baseline, or in the event of new symptoms suggestive of new extramedullary disease, cord compression, new fracture, etc., or to investigate new hypercalcaemia. In the rare event of participants with extramedullary disease at baseline, imaging should be performed at the end of IRD induction to confirm the end of induction response, and at any other time the disease parameters suggest that the participant has achieved a complete response (if not already achieved at the end of induction) to confirm that the extramedullary disease has resolved.
f. Performance status should be recorded at the same timepoints as the frailty index (i.e. prior to R1, after cycles 2, 4, 6 and 12 of induction, and after cycles 6 and 12 of maintenance). Performance status should also be recorded at the time of disease progression. Or at 2, 6 and 12 months post-R1 if treatment is stopped prior to this for reasons other than disease progression.
g. If the participant did not consent to the pre-trial bone marrow registration part of Myeloma XIV, the bone marrow biopsy will need to be taken after full informed consent.
[¥] If not sent at bone marrow and blood sample consent.
* At pre-randomisation 1 a CD138 negative portion of the bone marrow aspirate will be sent to LIMR after CD138 positive selection at ICR.
** At the end of induction bone marrow aspirate will be sent to ICR after CD138 positive selection at LIMR.

For peer review only

Myeloma XIV Supplementary Material to Protocol Paper
EudraCT number: **2018-003590-10**

Supplementary Material:

Contents

Glossary of Terms 2
List of Sites in the Fitness Trial 4
Spirit Checklist : Reporting checklist for protocol of a clinical trial. 5
Renal and Liver Function 11
Concomitant Medication 12
Exploratory analysis 13
Subgroup analysis 13
Informed Consent Material 15
  Bone marrow and Blood sample consent form 15
  Main Trial consent 18
Central Sample Analysis 21
References 23

For peer review only

For peer review only - http://bmjopen.bmj.com/site/about/guidelines.xhtml

Myeloma XIV Supplementary Material to Protocol Paper
EudraCT number: **2018-003590-10**

Glossary of Terms

AE	Adverse event
ADL	Katz Activity of Daily Living
ALT	Alanine aminotransferase
AR	Adverse reaction
ANC	Absolute Neutrophil Count
AST	Aspartate aminotransferase
β2M	Beta2-microglobulin
CCI	Charlson Comorbidity Index
CI	Chief Investigator
CR	Complete response
CRF	Case report form
CTCAE	Common Terminology Criteria for Adverse Events
CTRU	Clinical Trials Research Unit
DMEC	Data Monitoring and Ethics Committee
eCRF	Electronic Case Report Form
GI	Gastrointestinal
HEAP	Health Economics Analysis Plan
HMDS	Haematological Malignancy Diagnostic Service
IADL	Lawton Instrumental Activity of Daily Living
IMiD	Immunomodulatory Drug
IMP	Investigational medicinal product
IMWG	International Myeloma Working Group
ITT	Intention to treat
mg	Milligram
MM	Multiple myeloma
MRD	Minimal Residual Disease
MR	Minimal response
NCI	National Cancer Institute
ORR	Overall response rate
OS	Overall survival
PD	Progressive disease
PFS	Progression-free survival
PFS2	Time from randomisation to second progression
PK	Pharmacokinetic(s)
PI	Proteasome Inhibitor
PR	Partial response
PS	Performance status
QoL	Quality of life
R1	Randomisation 1
R2	Randomisation 2
REC	Research Ethics Committee
SD	Stable disease
SAE	Serious adverse event
SAR	Serious adverse reaction
SPM	Secondary Primary Malignancy
SmPC	Summary of product characteristics

For peer review only

Myeloma XIV Supplementary Material to Protocol Paper
EudraCT number: **2018-003590-10**

SUSAR	Suspected unexpected serious adverse reaction
TMG	Trial Management Group
TNE	Transplant non-eligible
TSC	Trial Steering Committee
UK-MRA	United Kingdom Myeloma Research Alliance
ULN	Upper Limit of normal
VGPR	Very good partial response
VTE	Venous thromboembolism

For peer review only

For peer review only - http://bmjopen.bmj.com/site/about/guidelines.xhtml

Myeloma XIV Supplementary Material to Protocol Paper
EudraCT number: **2018-003590-10**

List of Sites in the Fitness Trial

Sites		
Aberdeen Royal Infirmary	Kettering general	Salford Royal Hospital
Blackpool Victoria	Kidderminster Hospital	Salisbury District Hospital
Bristol Haematology & Oncology Centre	Kings College Hospital, Denmark Hill	South Tyneside Hospital
Calderdale Royal Hospital	Leicester Royal Infirmary	Southampton Hospital
Castle Hill Hospital	Lincoln County Hospital	Southmead Hospital
Cheltenham Hospital	Maidstone Hospital	St Bartholomew Hospital
Clatterbridge Cancer Centre	Manchester Royal Infirmary	St George's Hospital, Tooting
Colchester District	New Cross Hospital	St Helens Hospital
Countess of Chester	Ninewells	St Helier Hospital
County Hospital	Norfolk & Norwich University Hospital	St Richard's Hospital
Croydon University Hospital	Nottingham City Hospital	St. James's University Hospital
Derriford Hospital	Peterborough City Hospital	St. John's. Hospital, Howden
Dewsbury and District Hospital	Pilgrim Hospital	Sunderland Royal Hospital
Dewsbury and District Hospital	Pontefract Hospital	The County Hospital, Wye Valley
Epsom General Hospital	Princess Royal	Torbay Hospital
Freeman Hospital	Queen Alexandra Hospital	TRAFFORD GENERAL HOSPITAL
Friarage Hospital	Queen Elizabeth Hospital the Queen Mother	University College London Hospital
Glan Clywd	Queen Elizabeth Hospital, Birmingham	Western General
Gloucestershire Royal	Queen Elizabeth Hospital, King's Lynn	Whiston Hospital
Goodhope	Queens Hospital, Romford	William Harvey
Grantham & District	Royal Albert Edward Infirmary	Worcestershire Royal Hospital
Guys Hospital	Royal Berkshire	Worcestershire Royal Hospital
Harrogate District Hospital	Royal Bournemouth General	Worthing Hospital
Heartlands	Royal Derby	Wrexham Maelor Hospital
Hillingdon Hospital	Royal Devon & Exeter Hospital	Wythenshaw Hospital
Hinchingbrooke Hospital	Royal Oldham	YSBYTY GWYNEDD
Huddersfield Royal Infirmary	Royal Stoke University Hospital	North Middlesex University Hospital
James Cook University Hospital	Royal United Hospital, Bath	Royal Hallamshire
Kent & Canterbury Hospital	Russells Hall Hospital	

*In total the trial will be conducted in 87 centres around the UK

For peer review only

Myeloma XIV Supplementary Material to Protocol Paper
EudraCT number: **2018-003590-10**

Spirit Checklist : Reporting checklist for protocol of a clinical trial.

Based on the SPIRIT guidelines.

Chan A-W, Tetzlaff JM, Gøtzsche PC, Altman DG, Mann H, Berlin J, Dickersin K, Hróbjartsson A, Schulz KF, Parulekar WR, Krleža-Jerić K, Laupacis A, Moher D. SPIRIT 2013 Explanation and Elaboration: Guidance for protocols of clinical trials. BMJ. 2013;346:e7586

		Reporting Item	Page Number
**Administrative information**			
Title	#1	Descriptive title identifying the study design, population, interventions, and, if applicable, trial acronym	1
Trial registration	#2a	Trial identifier and registry name. If not yet registered, name of intended registry	1,2
Trial registration: data set	#2b	All items from the World Health Organization Trial Registration Data Set	NA
Protocol version	#3	Date and version identifier	18
Funding	#4	Sources and types of financial, material, and other support	20
Roles and responsibilities: contributorship	#5a	Names, affiliations, and roles of protocol contributors	1, 20
Roles and responsibilities: sponsor contact information	#5b	Name and contact information for the trial sponsor	20
Roles and responsibilities: sponsor and funder	#5c	Role of study sponsor and funders, if any, in study design; collection, management, analysis, and interpretation of data; writing of the report; and the decision to submit the report for publication, including whether they will have ultimate authority over any of these activities	20,21
Roles and responsibilities: committees	#5d	Composition, roles, and responsibilities of the coordinating centre, steering committee, endpoint adjudication committee, data management team, and other individuals or groups overseeing the trial, if applicable (see Item 21a for data monitoring committee)	16
**Introduction**			

For peer review only - http://bmjopen.bmj.com/site/about/guidelines.xhtml

Myeloma XIV Supplementary Material to Protocol Paper
EudraCT number: **2018-003590-10**

Background and rationale	#6a	Description of research question and justification for undertaking the trial, including summary of relevant studies (published and unpublished) examining benefits and harms for each intervention	4,5
Background and rationale: choice of comparators	#6b	Explanation for choice of comparators	4,5,6
Objectives	#7	Specific objectives or hypotheses	6
Trial design	#8	Description of trial design including type of trial (eg, parallel group, crossover, factorial, single group), allocation ratio, and framework (eg, superiority, equivalence, non-inferiority, exploratory)	6
**Methods: Participants, interventions, and outcomes**			
Study setting	#9	Description of study settings (eg, community clinic, academic hospital) and list of countries where data will be collected. Reference to where list of study sites can be obtained	6
Eligibility criteria	#10	Inclusion and exclusion criteria for participants. If applicable, eligibility criteria for study centres and individuals who will perform the interventions (eg, surgeons, psychotherapists)	6
Interventions: description	#11a	Interventions for each group with sufficient detail to allow replication, including how and when they will be administered	9
Interventions: modifications	#11b	Criteria for discontinuing or modifying allocated interventions for a given trial participant (eg, drug dose change in response to harms, participant request, or improving / worsening disease)	9,10
Interventions: adherance	#11c	Strategies to improve adherence to intervention protocols, and any procedures for monitoring adherence (eg, drug tablet return; laboratory tests)	10
Interventions: concomitant care	#11d	Relevant concomitant care and interventions that are permitted or prohibited during the trial	10

For peer review only - http://bmjopen.bmj.com/site/about/guidelines.xhtml

Outcomes	#12	Primary, secondary, and other outcomes, including the specific measurement variable (eg, systolic blood pressure), analysis metric (eg, change from baseline, final value, time to event), method of aggregation (eg, median, proportion), and time point for each outcome. Explanation of the clinical relevance of chosen efficacy and harm outcomes is strongly recommended	11
Participant timeline	#13	Time schedule of enrolment, interventions (including any run-ins and washouts), assessments, and visits for participants. A schematic diagram is highly recommended (see Figure)	11,12
Sample size	#14	Estimated number of participants needed to achieve study objectives and how it was determined, including clinical and statistical assumptions supporting any sample size calculations	12,13
Recruitment	#15	Strategies for achieving adequate participant enrolment to reach target sample size	13
**Methods: Assignment of interventions (for controlled trials)**			
Allocation: sequence generation	#16a	Method of generating the allocation sequence (eg, computer-generated random numbers), and list of any factors for stratification. To reduce predictability of a random sequence, details of any planned restriction (eg, blocking) should be provided in a separate document that is unavailable to those who enrol participants or assign interventions	13,14
Allocation concealment mechanism	#16b	Mechanism of implementing the allocation sequence (eg, central telephone; sequentially numbered, opaque, sealed envelopes), describing any steps to conceal the sequence until interventions are assigned	13
Allocation: implementation	#16c	Who will generate the allocation sequence, who will enrol participants, and who will assign participants to interventions	14
Blinding (masking)	#17a	Who will be blinded after assignment to interventions (eg, trial participants, care	14

For peer review only

		providers, outcome assessors, data analysts), and how	
Blinding (masking): emergency unblinding	#17b	If blinded, circumstances under which unblinding is permissible, and procedure for revealing a participant's allocated intervention during the trial	14
**Methods: Data collection, management, and analysis**			
Data collection plan	#18a	Plans for assessment and collection of outcome, baseline, and other trial data, including any related processes to promote data quality (eg, duplicate measurements, training of assessors) and a description of study instruments (eg, questionnaires, laboratory tests) along with their reliability and validity, if known. Reference to where data collection forms can be found, if not in the protocol	14
Data collection plan: retention	#18b	Plans to promote participant retention and complete follow-up, including list of any outcome data to be collected for participants who discontinue or deviate from intervention protocols	14
Data management	#19	Plans for data entry, coding, security, and storage, including any related processes to promote data quality (eg, double data entry; range checks for data values). Reference to where details of data management procedures can be found, if not in the protocol	14,15
Statistics: outcomes	#20a	Statistical methods for analysing primary and secondary outcomes. Reference to where other details of the statistical analysis plan can be found, if not in the protocol	15,16
Statistics: additional analyses	#20b	Methods for any additional analyses (eg, subgroup and adjusted analyses)	16
Statistics: analysis population and missing data	#20c	Definition of analysis population relating to protocol non-adherence (eg, as randomised analysis), and any statistical methods to handle missing data (eg, multiple imputation)	16,17
**Methods: Monitoring**			

For peer review only

Data monitoring: formal committee	#21a	Composition of data monitoring committee (DMC); summary of its role and reporting structure; statement of whether it is independent from the sponsor and competing interests; and reference to where further details about its charter can be found, if not in the protocol. Alternatively, an explanation of why a DMC is not needed	16,17
Data monitoring: interim analysis	#21b	Description of any interim analyses and stopping guidelines, including who will have access to these interim results and make the final decision to terminate the trial	16,17
Harms	#22	Plans for collecting, assessing, reporting, and managing solicited and spontaneously reported adverse events and other unintended effects of trial interventions or trial conduct	17,18
Auditing	#23	Frequency and procedures for auditing trial conduct, if any, and whether the process will be independent from investigators and the sponsor	18
**Ethics and dissemination**			
Research ethics approval	#24	Plans for seeking research ethics committee / institutional review board (REC / IRB) approval	18
Protocol amendments	#25	Plans for communicating important protocol modifications (eg, changes to eligibility criteria, outcomes, analyses) to relevant parties (eg, investigators, REC / IRBs, trial participants, trial registries, journals, regulators)	18
Consent or assent	#26a	Who will obtain informed consent or assent from potential trial participants or authorised surrogates, and how (see Item 32)	19
Consent or assent: ancillary studies	#26b	Additional consent provisions for collection and use of participant data and biological specimens in ancillary studies, if applicable	19
Confidentiality	#27	How personal information about potential and enrolled participants will be collected, shared, and maintained in order to protect confidentiality before, during, and after the trial	19
Declaration of interests	#28	Financial and other competing interests for principal investigators for the overall trial and each study site	20,21

For peer review only - http://bmjopen.bmj.com/site/about/guidelines.xhtml

Myeloma XIV Supplementary Material to Protocol Paper
EudraCT number: **2018-003590-10**

Data access	#29	Statement of who will have access to the final trial dataset, and disclosure of contractual agreements that limit such access for investigators	19
Ancillary and post trial care	#30	Provisions, if any, for ancillary and post-trial care, and for compensation to those who suffer harm from trial participation	19
Dissemination policy: trial results	#31a	Plans for investigators and sponsor to communicate trial results to participants, healthcare professionals, the public, and other relevant groups (eg, via publication, reporting in results databases, or other data sharing arrangements), including any publication restrictions	19
Dissemination policy: authorship	#31b	Authorship eligibility guidelines and any intended use of professional writers	19
Dissemination policy: reproducible research	#31c	Plans, if any, for granting public access to the full protocol, participant-level dataset, and statistical code	19
**Appendices**			
Informed consent materials	#32	Model consent form and other related documentation given to participants and authorised surrogates	15 (Supplementary Material)
Biological specimens	#33	Plans for collection, laboratory evaluation, and storage of biological specimens for genetic or molecular analysis in the current trial and for future use in ancillary studies, if applicable	21 (Supplementary Material)

None The SPIRIT Explanation and Elaboration paper is distributed under the terms of the Creative Commons Attribution License CC-BY-NC. This checklist can be completed online using https://www.goodreports.org/, a tool made by the EQUATOR Network in collaboration with Penelope.ai

For peer review only - http://bmjopen.bmj.com/site/about/guidelines.xhtml

Myeloma XIV Supplementary Material to Protocol Paper
EudraCT number: **2018-003590-10**

Renal and Liver Function

As per standard of care, doses of lenalidomide and ixazomib should be adjusted at baseline according to liver and renal function. Regardless of which dosing arm has been assigned the doses indicated in this section should not be exceeded on cycle 1 day 1. Doses of lenalidomide reduced (based on renal function) can only be escalated, if tolerated, to the maximum dose indicated by the participant's assigned dosing arm/strategy after cycle 1 as per the lenalidomide SPC. Ixazomib doses may **not** be escalated, whether reduced based on renal or liver function.

Renal function:

Creatinine clearance should be calculated using the Cockcroft-Gault formula as the estimated GFR produced in most hospitals is not accurate in older patients.

	**Calculated creatinine Clearance (using Cockcroft-Gault formula)**	**Lenalidomide dose** (Days 1-21)	**Ixazomib dose** (Day 1, 8, 15)
Moderate renal impairment	≥ 30 to < 50 ml/min	10mg once daily	4mg once daily
Severe renal impairment	< 30 ml/min; not requiring dialysis	15mg every other day	3mg once daily
End stage renal disease	< 30 ml/min; requiring dialysis	5mg once daily (on dialysis days should be administered following dialysis)	3mg once daily

Liver function:

The reduced ixazomib dose of 3 mg is recommended in patients with moderate liver dysfunction characterised by total bilirubin > 1.5-3 x ULN (patients with bilirubin > 3 x ULN are not eligible for the study). No dose reduction based on AST/ALT is required.

Lenalidomide has not formally been studied in patients with impaired hepatic function and there are no specific dose recommendations

For peer review only - http://bmjopen.bmj.com/site/about/guidelines.xhtml

Myeloma XIV Supplementary Material to Protocol Paper
EudraCT number: **2018-003590-10**

Concomitant Medication

Permitted Concomitant Medication:

1) Antiemetics, including 5-HT3 serotonin receptor antagonists, may be used at the discretion of the investigator (however, note that dexamethasone additional to that given as part of the trial treatment schedule is **not** permitted).
2) Loperamide or other anti-diarrhoeal should be used for symptomatic non-infective diarrhoea at the discretion of the investigator. The dose and regimen will be according to institutional guidelines. Intravenous fluids should be given to prevent volume depletion.
3) Growth factors (e.g. granulocyte colony stimulating factor [G-CSF], thrombopoeitin receptor agonists (TPO) and recombinant erythropoietin) are permitted at the discretion of the PI and/or institutional practice. Due consideration of the increased risk of VTE with TPO agonists and erythropoietin should be given and prophylaxis adjusted accordingly.
4) Participants should be transfused with red cells and platelets as clinically indicated and according to institutional guidelines. Platelet transfusions are not allowed within 3 days prior to study drug dosing for any dosing day in order to meet pre-treatment criteria, to ensure that the platelet count is at a safe level to commence further treatment.
5) Patients known to be Hep B core antibody positive should receive prophylaxis with an appropriate anti-viral as per local protocols and after consultation with a gastroenterology/hepatology specialist.
6) Other supportive measures consistent with optimal participant care may be given throughout the study.

Excluded concomitant Medication:

1) Any antineoplastic treatment with activity against MM, other than study drugs and localised radiotherapy for pain relief.
2) Platelet transfusions within 3 days prior to study drug dosing for any dosing day in order to meet pre-treatment criteria, or prior to randomisation in order to meet eligibility criteria.
3) Additional steroid therapy (not as trial IMP) (except: before commencing trial treatment up to a maximum of 160 mg dexamethasone or equivalent based on glucocorticoid activity per inclusion criteria, or for the management of rash in which case topical, IC or oral steroid at a dose not exceeding prednisolone 10mg per day or equivalent is permitted)
4) Radiotherapy for the treatment of symptoms related to disease progression (in the event of disease progression participant should stop trial treatment and further treatment will be at the discretion of the treating clinician).
5) Excluded foods and dietary supplements include St. John's Wort.
6) Live vaccines are prohibited 30 days prior to R1 and for the duration of the study. They are also prohibited for 3 months after the last dose of study drug.

The following treatments should be avoided, unless deemed necessary by the treating clinician:

1) Systemic treatment with any of the following metabolising enzyme inducers should be avoided, unless there is no appropriate alternative medication for the participant's use (Rationale: If there were to be a drug-drug interaction with an inducer, ixazomib exposure would be decreased):
2) Strong CYP3A inducers: rifampicin, rifabutin, carbamazepine, phenytoin, & phenobarbital
3) Non-steroidal anti-inflammatory drugs (NSAIDs) should be avoided especially in those with abnormal renal function unless the clinical benefit outweighs the risk to deteriorating renal impairment.

For peer review only - http://bmjopen.bmj.com/site/about/guidelines.xhtml

Myeloma XIV Supplementary Material to Protocol Paper
EudraCT number: **2018-003590-10**

Exploratory analysis

Exploratory analysis will consider; the association of molecular subgroups with outcome through the investigation of frailty biomarkers, differences between the Karnofsky Performance Status (PS) and ECOG PS, and the prospective validation the UK-MRA MRP (United Kingdom-Myeloma Research Alliance Myeloma Risk Profile). All exploratory analysis to be conducted by the trial statisticians will be pre-specified in the statistical analysis plan.

Subgroup analysis

A series of subgroup analyses will be conducted to determine whether a selection of patient characteristics, haematology / local serological results, biochemistry results and cytogenetic/molecular results as well as response assessments are prognostic of the endpoints; Progression-Free survival, Progression-free survival 2, overall survival, attainment of ≥VGPR (Very Good Partial Response) and attainment of Minimal Residual Disease (MRD) negativity, as defined for both randomisations. Note that the subgroups MRD response to IRD (Ixazomib, Lenalidomide and Dexamethasone) induction and MRD response at 12m maintenance will not be analysed for the attainment of MRD negativity endpoint.

Patient Characteristics

- Age at R1 baseline (<70,70-80,>80)
- Sex (Male / Female)
- ECOG performance status at R1 baseline (0,1, ≥2, missing)
- Karnofsky performance status at R1 baseline (<80, 80-90,>90, missing)
- ISS at R1 baseline (Stage I, Stage II, Stage III, missing)
- R-ISS at R1 baseline (Stage I, Stage II, Stage III, missing)
- IMWG frailty category at R1 baseline (Fit, Unfit, Frail)
- MRP frailty category at R1 baseline (Fit, Intermediate-Fitness, Frail, missing)

Hematology / Local Serological Results

- Haemaglobin concentration at R1 baseline (<100, ≥100 g/L)
- Platelets at R1 baseline (<150, ≥150 x109/L)
- White blood cells at R1 baseline (<LLN, ≥LLN, missing)
- Neutrophil count at R1 baseline (<LLN, ≥LLN)
- Lymphocyte count at R1 baseline (<LLN, ≥LLN, missing)
- Plasma cells (%) in bone marrow at R1 baseline (<60%, ≥60%)

Biochemistry Results

- β-2 microglobulin concentration at R1 baseline (<3.5, 3.5–<5.5, ≥5.5mg/L)
- Serum creatinine concentration at R1 baseline (<175, ≥175 µmol/L)
- Corrected serum calcium concentration at R1 baseline (<2.75, ≥2.75 mmol/L)
- Serum albumin at R1 baseline (<ULN, ≥ULN, missing)
- Lactate dehydrogenase at R1 baseline (<ULN, ≥ULN, missing)
- C-Reactive protein at R1 baseline (<ULN, ≥ULN, missing)
- Total bilirubin at R1 baseline (<ULN, ≥ULN)
- ALT/AST at R1 baseline (<ULN, ≥ULN)

For peer review only - http://bmjopen.bmj.com/site/about/guidelines.xhtml

Myeloma XIV Supplementary Material to Protocol Paper
EudraCT number: **2018-003590-10**

MLPA Cytogenetic Results

- t(4,14) at R1 baseline (detected, not detected)
- t(14,16) at R1 baseline (detected, not detected)
- t(14,20) at R1 baseline (detected, not detected)
- del(17p) at R1 baseline (detected, not detected)
- del(13q) at R1 baseline (detected, not detected)
- del(1p) at R1 baseline (detected, not detected)
- gain(1q) at R1 baseline (detected, not detected)
- UK definition risk group at R1 baseline (Standard, high risk, ultra-high risk)
- IMWG definition risk group at R1 baseline (Adverse, Standard)

Response

- Response to IRD induction (<VGPR, ≥ VGPR)
- Response to 12m maintenance (<VGPR, ≥ VGPR)
- MRD response to IRD induction (MRD positive, MRD Negative)
- MRD response to 12m maintenance (MRD positive, MRD Negative)

Further subgroup analyses may be undertaken, as appropriate and all subgroup analysis will be fully documented in the statistical analysis plan. Subgroup and exploratory analyses may, by chance, generate false negative or positive results. Those carried out will be interpreted with caution.

For peer review only

For peer review only - http://bmjopen.bmj.com/site/about/guidelines.xhtml

Myeloma XIV Supplementary Material to Protocol Paper
EudraCT number: **2018-003590-10**

Informed Consent Material

Bone marrow and Blood sample consent form

Delete this line, then print on Trust/Hospital headed paper

Participant ID:	Initials:
Date of Birth:	NHS/Hospital Number:
EudraCT Number:	Principal Investigator:

FiTNEss UK-MRA Myeloma XIV

PARTICIPANT CONSENT FORM FOR BONE MARROW AND BLOOD SAMPLE

Please initial each box

1. I confirm that I have read and understand the information sheet for the bone marrow and blood sample request and have had the opportunity to ask questions. ☐
2. I understand that my providing bone marrow and blood samples is voluntary and that I am free to withdraw at any time without my medical care or legal rights being affected. ☐
3. I understand that the samples will be sent along with information about me, which will include NHS number and may include my name. ☐
4. I agree to these samples being stored and used for research investigations that form part of the FiTNEss (UK-MRA Myeloma XIV) study, and understand that this will include genetic research. ☐
5. I understand that a unique reference number will be allocated to the samples which may allow them to be linked back to me in future for research purposes. ☐
6. I understand that my data may be shared on a collaborative basis with researchers in the UK and potentially, centres abroad, including outside the European Economic Area. ☐

For peer review only - http://bmjopen.bmj.com/site/about/guidelines.xhtml

Myeloma XIV Supplementary Material to Protocol Paper
EudraCT number: **2018-003590-10**

7. I agree to allow any information provided to be used for healthcare and/or further medical research upon the understanding that my identity will remain anonymous wherever possible. ☐

8. I understand that results of my laboratory tests may be shared with other research teams if I am taking part in another clinical study being run by the University of Leeds. ☐

9. I agree to a copy of this Consent Form being sent to the Clinical Trials Research Unit. ☐

10. I agree for my details (which will include date of birth and NHS number) to be submitted to NHS Digital so that information about my health, and mortality should this become available, may be obtained by the CTRU if necessary. ☐

11. I agree to provide bone marrow and blood samples. ☐

For peer review only

Please indicate your wishes in the below scenarios:

Please tick or initial yes or no:

Please tick ✓ or initial

A) If I am diagnosed with a plasma cell dyscrasia, other than myeloma, I give permission for the samples sent to the central laboratories to be stored and used in future research that receives ethical approval. I understand that the samples and data collected from them may be shared with researchers, possibly outside the EEA.

Yes ☐ No ☐

B) If I am diagnosed with myeloma but do not take part in the FiTNEss (UK-MRA Myeloma XIV) study, I give permission for the samples sent to the central laboratories to be stored and used in future research that receives ethical approval. I understand that the samples and data collected from them may be shared with researchers, possibly outside the EEA.

Yes ☐ No ☐

Patient (to be completed by the patient):

Signature………..

Name (block capitals)…………………………………………………………………………………………….

Date……....

Investigator:

I have explained the request to the above named patient and he/she has indicated his/her willingness for samples of bone marrow and blood to be sent to the FiTNEss central laboratories.

Signature………

Name (block capitals)…………………………………………………………………………………………….

Date……

(If used)Translator:

Signature………

Name (block capitals)…………………………………………………………………………………………….

Date……

(1 copy for patient; 1 for the CTRU; 1 held in patient notes, original stored in Investigator Site File)

.

For peer review only - http://bmjopen.bmj.com/site/about/guidelines.xhtml

For peer review only

Myeloma XIV Supplementary Material to Protocol Paper
EudraCT number: **2018-003590-10**

Main Trial consent

Delete this line, then print on Trust/Hospital headed paper

Participant ID:	Initials:
Date of Birth:	NHS/Hospital Number:
EudraCT Number:	Principal Investigator:

FiTNEss
UK-MRA Myeloma XIV

PARTICIPANT CONSENT FORM

Please initial each box

1. I confirm that I have read and understand the information sheet for the above study and have had the opportunity to ask questions.

2. I understand that my participation in this study is voluntary and that I am free to withdraw at any time without my medical care or legal rights being affected. I understand that even if I withdraw from the above study, the data and samples collected from me will be used in analysing the results of the study, and in some cases further information about any unwanted effects of my treatment may need to be collected by the study team.

3. I understand that my healthcare records may be looked at by authorised individuals from the study team, regulatory bodies or Sponsor in order to check that the study is being carried out correctly.

4. I understand that I will be asked questions about how independent my lifestyle is. I understand that this information will be used to generate a score of frailty and may affect the doses of treatment that I receive.

5. I am aware that I am required to have samples taken and sent to a central laboratory and I understand that this will include genetic research. I understand that the samples will be sent along with

For peer review only - http://bmjopen.bmj.com/site/about/guidelines.xhtml

information about me, which will include NHS number and may include my name.

6. I understand that results or reports from imaging scans, blood tests or other investigations (including for additional cancers) may be sent to the Clinical Trials Research Unit for central review by the research team. ☐

7. I agree to allow any information or results arising from this study to be used for healthcare and/or further medical research upon the understanding that my identity will remain anonymous wherever possible. ☐

8. I understand that my data and results of my laboratory tests (blood, urine and bone marrow sample collections) may be shared with other research teams if I am taking part in another clinical study being run by the University of Leeds. ☐

9. I agree for my details (which will include date of birth and NHS number) to be submitted to NHS Digital so that information about my health, and mortality should this become available, may be obtained by the CTRU if necessary. ☐

10. I understand that my GP, or any other doctor treating me, will be notified of my participation in this study. ☐

11. I understand that some of my data may be passed to other organisations, such as the pharmaceutical companies Takeda and Celgene, (possibly in other countries outside the EEA where the data protection standards and laws may be different from the UK) to monitor the safety of the treatment(s) that I am receiving. I understand that my identity will remain anonymous. ☐

12. I agree to a copy of this Consent Form being sent to the Clinical Trials Research Unit ☐

13. I agree to take part in the study. ☐

For peer review only

Myeloma XIV Supplementary Material to Protocol Paper
EudraCT number: **2018-003590-10**

The following parts of the study are OPTIONAL (see part 3 of the Participant Information Sheet). Even if you agree to take part in the FiTNEss study, you do not have to agree to these optional parts. Please tick or initial yes or no.

Please tick ✓ or initial

	Yes	No
A) I agree to take part in the Quality of Life and Healthcare Resource Use studies by completing questionnaires at time points throughout the study.	☐	☐
B) I give permission for samples that I provide to be used in future research that receives ethical approval, and I understand that this includes the blood and bone marrow samples provided at the time of my diagnosis (if consented separately to the bone marrow and blood sample for this study). I understand that my samples and data collected from them may be shared on a collaborative basis with researchers in the UK and potentially, centres abroad, including outside the European Economic Area.	☐	☐

Patient (to be completed by the patient):

Signature……………………………………………………………………………………

Name (block capitals)…………………………………………………………………………

Date……………………………………………………………………………………………

Investigator:

I have explained the study to the above named patient and he/she has indicated his/her willingness to participate.

Signature……………………………………………………………………………………

Name (block capitals)…………………………………………………………………………

Date……………………………………………………………………………………………

(If used)Translator:

Signature……………………………………………………………………………………

Name (block capitals)…………………………………………………………………………

Date……………………………………………………………………………………………

For peer review only - http://bmjopen.bmj.com/site/about/guidelines.xhtml

Myeloma XIV Supplementary Material to Protocol Paper
EudraCT number: **2018-003590-10**

Central Sample Analysis

The trial has four central laboratories, each receiving samples for trial purposes. The time points and the reason for analysis for each central sample can be found in Figure 4. Details on the analysis of each sample is below. Note that participants can consent for their samples to be used in future research and hence each laboratory can store their trial samples for use in future studies. The storage details are also included below.

Birmingham

Samples are sent in approved packaging via the UK post office service and received in a UKAS accredited clinical laboratory for laboratory assessment and storage under the direction of the TMG. Assessment of prognostic markers, markers of disease activity and M-protein levels. Analyses will include serum levels of beta-2 microglobulin, immunoglobulins IgG, IgA and IgM, kappa and lambda free immunoglobulin light chains. Serum electrophoresis and immunofixation, M-protein quantitation including total protein and densitometry. Urine immunofixation and free light chain quantitation will be performed where appropriate.

HMDS

The presence of minimal residual disease will be assessed in bone marrow aspirates using a validated flow cytometry assay (sensitivity ≤0.004%) performed at a single central laboratory (HMDS, Leeds Teaching Hospitals Trust). A minimum of 500,000 cells will be evaluated with six- or eight-colour antibody combinations including CD138, CD38, CD45, CD19, CD56, CD27, CD81 and CD117.

Sustained MRD-negativity, as defined by the IMWG [1], will not be assessed as it requires the participant to be imaging MRD-negative at the same timepoints that they are flow MRD-negative and imaging is only mandated at baseline and to investigate new systems not at regular intervals for all participants.

Flow MRD will be assessed at the following time points: Baseline (pre-randomisation 1), after 6 cycles of induction therapy, at the end of 12 cycles of induction treatment (or at the end of the final cycle of IRD treatment if sooner), at 12 months post maintenance randomisation 2. Flow MRD will also be assessed at any point a first occurrence of CR, or SCR is suspected.

During final analysis, we will determine the conversion rate of patients who are MRD Positive at the end of induction to MRD Negative at 12 months post Randomisation 2. The analysis to this is specified within our Statistical Analysis Plan (SAP).

Samples will be used for trial purposes only and will not be stored for future research.

ICR

Myeloma tumor material is purified from fresh bone marrow material using CD138 immunomagnetic separation. Tumor DNA and RNA are extracted and recurrent immunoglobulin translocations t(4;14), t(14;16), t(14;20) and copy number aberrations del(1p32), gain(1q21), del(17p) investigated as previously published using qRT-PCR and multiplex ligation-dependent amplification (MLPA), respectively[2]. Excess material is stored, if patient gave written consent for use in future research, at -80C standard DNA and RNA storage conditions.

LIMR

For peer review only - http://bmjopen.bmj.com/site/about/guidelines.xhtml

Myeloma XIV Supplementary Material to Protocol Paper
EudraCT number: **2018-003590-10**

Peripheral blood samples for immunosenescent and Senescence-associated secretory proteome will be draw and managed timepoints in the clinical pathway, processed in a central laboratory and stored as aliquots of mononuclear cells and serum for subsequent planned use. Full QA is performed on sample receipt to ensure only viable and valuable samples are processed and stored for research use.

For peer review only

For peer review only - http://bmjopen.bmj.com/site/about/guidelines.xhtml

Myeloma XIV Supplementary Material to Protocol Paper
EudraCT number: **2018-003590-10**

References

1. Kumar, S.P., et al., *International Myeloma Working Group consensus criteria for response and minimal residual disease assessment in multiple myeloma.* The lancet oncology, 2016. **17**(8): p. e328-e346.
2. Shah, V., et al., *Prediction of outcome in newly diagnosed myeloma: a meta-analysis of the molecular profiles of 1905 trial patients.* Leukemia, 2018. **32**(1): p. 102-110.

For peer review only

For peer review only - http://bmjopen.bmj.com/site/about/guidelines.xhtml

BMJ Open

Frailty-adjusted therapy in Transplant Non-Eligible patients with newly diagnosed Multiple Myeloma (FiTNEss (UK-MRA Myeloma XIV Trial)): A study protocol for a randomised phase III trial

Journal:	*BMJ Open*
Manuscript ID	bmjopen-2021-056147.R2
Article Type:	Protocol
Date Submitted by the Author:	02-Mar-2022

Complete List of Authors:	Coulson, Amy; University of Leeds Clinical Trials Research Unit, Leeds Institute of Clinical Trials Research<br>Royle, Kara-Louise; University of Leeds Clinical Trials Research Unit, Leeds Institute of Clinical Trials Research<br>Pawlyn, Charlotte; Cancer Research UK London Research Institute<br>Cairns, David; University of Leeds Clinical Trials Research Unit, Leeds Institute of Clinical Trials Research<br>Hockaday, Anna; University of Leeds Clinical Trials Research Unit, Leeds Institute of Clinical Trials Research<br>Bird, Jennifer; University Hospitals Bristol and Weston NHS Foundation Trust, Department of Haematology<br>Bowcock, Stella; Princess Royal Hospital, Department of Haematology, Kings college Hospital NHS Foundation Trust<br>Kaiser, Martin; Institute of Cancer Research; Royal Marsden Hospital NHS Trust, The Department of Haemato-oncology<br>de Tute, Ruth; University of Leeds, Haematology Malignancy Diagnostic Service (HMDS)<br>Rabin, Neil; University College London Hospitals NHS Foundation Trust, Department of Haematology<br>Boyd, Kevin; Royal Marsden Hospital NHS Trust, The Department of Haemato-oncology<br>Jones, John; King's College Hospital; Eastbourne District General Hospital<br>Parrish, Christopher; Leeds Teaching Hospitals NHS Trust, Department of Haematology<br>Gardner, Hayley; University Hospitals Birmingham NHS Foundation Trust, Department of Haematology and Stem Cell Transplantation<br>Meads, David; University of Leeds Leeds Institute of Health Sciences, Academic Unit of Health Economics<br>Dawkins, Bryony ; University of Leeds Leeds Institute of Health Sciences, Academic Unit of Health Economics<br>Olivier, Catherine; University of Leeds Clinical Trials Research Unit, Leeds Institute of Clinical Trials Research<br>Henderson, Rowena; University of Leeds Clinical Trials Research Unit, Leeds Institute of Clinical Trials Research<br>Best, Phillip; University of Leeds Clinical Trials Research Unit, Leeds Institute of Clinical Trials Research<br>Owen, Roger; St James's University Hospital, Haematology Malignancy

	Diagnostic Service (HMDS)<br>Jenner, Matthew ; Southampton General Hospital<br>Kishore, Bhuvan; University Hospitals Birmingham NHS Foundation Trust, Department of Haematology and Stem Cell Transplantation,<br>Drayson, Mark ; University of Birmingham, Institute of Immunology and Immunotherapy University of Birmingham Department of Haematology<br>Jackson, Graham; Newcastle University, Department of Haematology<br>Cook, Gordon; University of Leeds Clinical Trials Research Unit, Leeds Institute of Clinical Trials Research; St James's University Hospital, Leeds Cancer Centre
**Primary Subject Heading**:	Haematology (incl blood transfusion)
Secondary Subject Heading:	Haematology (incl blood transfusion), Qualitative research, Health economics, Oncology
Keywords:	Myeloma < HAEMATOLOGY, Myeloma < ONCOLOGY, CHEMOTHERAPY, Clinical trials < THERAPEUTICS

SCHOLARONE™
Manuscripts

Frailty-adjusted therapy in Transplant Non-Eligible patients with newly diagnosed Multiple Myeloma (FiTNEss (UK-MRA Myeloma XIV Trial)): A study protocol for a randomised phase III trial

Amy Beth Coulson[1], Kara-Louise Royle[1], Charlotte Pawlyn[2], David A Cairns[1], Anna Hockaday[1], Jennifer Bird[3], Stella Bowcock[4], Martin Kaiser[2,5], Ruth De Tute[6], Neil Rabin[7], Kevin Boyd[5], John Jones[15,16,17], Christopher Parrish[14], Hayley Gardner[9], David Meads[10], Bryony Dawkins[10], Catherine Olivier[1], Rowena Henderson[1], Phillip Best[1], Roger Owen[6], Matthew Jenner[11], Bhuvan Kishore[9], Mark Drayson[12], Graham Jackson[13] and Gordon Cook[1,8*]

1. Clinical Trials Research Unit, Leeds Institute of Clinical Trials Research, University of Leeds, Leeds, United Kingdom
2. The Institute of Cancer Research, London, United Kingdom
3. Department of Haematology, University Hospitals Bristol and Weston NHS Trust , Bristol, UK
4. Department of Haematology, King's College Hospital NHS Foundation Trust, Princess Royal Hospital, London, UK
5. The Department of Haemato-oncology, The Royal Marsden NHS Foundation Trust, London, UK
6. HMDS, St James's University Hospital, Leeds, UK
7. The Department of Haematology, University College London Hospitals NHS Trust, London
8. Leeds Cancer Centre, St James's University Hospital, Leeds, United Kingdom
9. Department of Haematology and Stem Cell Transplantation, University Hospitals Birmingham NHS Foundation Trust, Birmingham
10. Academic Unit of Health Economics, Leeds Institute of Health Sciences, University of Leeds, Leeds, UK
11. University Hospital Southampton NHS Foundation Trust
12. Institute of Immunology and Immunotherapy University of Birmingham Department of Haematology
13. Department of Haematology, Newcastle University, Newcastle-upon-Tyne, United Kingdom
14. Department of Haematology, Leeds Teaching Hospitals NHS Trust, Leeds, UK
15. Brighton and Sussex Medical School, Brighton, UK
16. King's College Hospital, London, UK.
17. Eastbourne District General Hospital, UK.

*Corresponding Author:

Gordon Cook, MB.ChB, PhD, FRCP(Glas), FRCPath

Leeds Cancer Centre

St James's University Hospital

Beckett Street, Leeds, UK, LS9 7TF

Tel: +44 113 206 7940 Fax: +44 113 206 8177

E-mail: g.cook@leeds.ac.uk

Word count: 6558

For peer review only - http://bmjopen.bmj.com/site/about/guidelines.xhtml

For peer review only

Abstract

Introduction

Multiple myeloma is a bone marrow cancer which predominantly affects older people. The incidence is increasing in an ageing population.

Over the last ten years patient outcomes have improved. However, this is less apparent in older, less fit patients, who are ineligible for stem cell transplant. Research is required in this patient group, taking into account frailty and aiming to improve: treatment tolerability, clinical outcomes and quality of life.

Methods and analysis

FiTNEss is a national, phase III, multicentre, randomised controlled trial comparing standard (reactive) and frailty-adjusted (adaptive) induction therapy delivery with ixazomib, lenalidomide and dexamethasone (IRD), and to compare maintenance lenalidomide to lenalidomide plus ixazomib, in patients with newly diagnosed multiple myeloma not suitable for stem cell transplant. Seven-Hundred and Forty participants will be registered into the trial to allow 720 and 478 to be randomised at induction and maintenance, respectively.

All participants will receive IRD induction with the dosing strategy randomised (1:1) at trial entry. Patients randomised to the standard, reactive arm will commence at the full dose followed by toxicity dependent reactive modifications. Patients randomised to the adaptive arm will commence at a dose level determined by their International Myeloma Working Group (IMWG) frailty score. Following 12 cycles of induction treatment participants alive and progression-free will undergo a second (double-blind) randomisation on a 1:1 basis to maintenance treatment with lenalidomide plus placebo versus lenalidomide plus ixazomib until disease progression or intolerance.

Ethics and dissemination

Ethical approval has been obtained from the North East - Tyne & Wear South Research Ethics Committee (19/NE/0125) and capacity and capability confirmed by local research and development departments for each participating centre prior to opening to recruitment. Participants are required to provide written informed consent prior to trial registration. Trial results will be disseminated by conference presentations and peer-reviewed publications.

Trial registration

ISRCTN17973108

ClinicalTrials.gov:NCT03720041.

Key Words: Multiple Myeloma, Transplant-ineligible, Frailty-adjusted, Randomised, Blinded

For peer review only - http://bmjopen.bmj.com/site/about/guidelines.xhtml

Article Summary

Strengths and limitations of this Study

- FiTNEss will provide the first prospective data investigating the use of the International Myeloma Working Group (IMWG) frailty score to define appropriate dose delivery strategies for older patients.
- FiTNEss will explore the impact of dual agent maintenance compared to the single agent standard of care using a gold standard placebo controlled design.
- The trial has the potential to meet a high unmet need in older myeloma patients for whom the impact of recent therapeutic have been less marked.
- Wide inclusion criteria have been designed to maximise the recruitment of older, more frail patients who may not previously have been included in clinical trials.
- Owing to the nature of assessments and dose adaptations blinding in the induction phase of the trial is infeasible.

For peer review only

For peer review only - http://bmjopen.bmj.com/site/about/guidelines.xhtml

Introduction

Multiple Myeloma

Multiple Myeloma is the second most common haematological malignancy with over 5500 patients diagnosed in the UK each year.[1] Myeloma is predominantly a disease of older people, with two thirds of patients aged over 70 years at diagnosis. The incidence is increasing as the population ages.

Over the last ten years the development of proteasome inhibitors (PI), immunomodulatory drugs (IMiD® agents) and improved supportive care, have ameliorated outcomes for myeloma patients such that the median overall survival is now more than 6 years for younger, fitter patients (Figure 1A).[2] However, the impact of these therapies has been less marked in the older or less fit population, particularly those over 75 years of age and/or ineligible for stem cell transplant (Figure 1B). Whilst outcomes in younger patients are largely driven by molecular risk factors present in the myeloma cell clone, there is no evidence that older myeloma patients have more biologically high-risk disease.[3] Differences in outcomes are likely to be accounted for by changes in patient physiology and/or increased treatment-related toxicity. Data from our previous trial, Myeloma XI (ISRCTN49407852) shows that as age increases the number of participants ceasing treatment due to choice or toxicity increases (Figure 2). This group therefore has a high unmet need for new, less toxic treatments and improved treatment delivery approaches.

Existing Evidence: Induction Therapy

Treatment

Lenalidomide is an immunomodulatory agent and thalidomide derivative available as an oral preparation, which is more potent *in vitro* and with a different adverse effect profile than thalidomide. For example, a major benefit of lenalidomide is the absence of associated neurotoxicity or sedation seen with thalidomide, making it more tolerable; however, there is a significant rate of myelosuppression (20%), which is not seen with thalidomide. Lenalidomide is licensed, in combination with dexamethasone, for use in newly diagnosed multiple myeloma patients who are not eligible for transplant (TNE).

With the caveats of cross-trial comparison, our recent analysis of the Myeloma XI trial suggests that the combination CRDa (cyclophosphamide, lenalidomide and attenuated dexamethasone) is not superior to Rd-continuous used in the FIRST study (NCT00689936) in terms of progression-free survival (PFS). In addition, Myeloma XI also showed that patients in the non-intensive pathway with only a minimal or partial response to induction therapy who were randomised to receive cyclophosphamide, bortezomib and dexamethasone (CVD) consolidation had better outcomes than those who did not (an increase in median PFS of 11 months (HR: 0.73; P=0.061)).[4] However, a significant proportion of patients (394/610; 64.6%) did not make it to this randomisation due to participant withdrawal (211; 53.6% of 394), death (96; 24.4%), progression (45; 11.4%) and other reasons (42; 10.7%). These data suggest that combining IMiD® agents and PI upfront may improve outcomes further, avoiding the early loss of patients and leading to improved responses, as we have seen with the carfilzomib, cyclophosphamide, lenalidomide and dexamethasone (KCRD) arm in the transplant-eligible pathway of the Myeloma XI study.[5 6]

Ixazomib (MLN9708) is an orally bioavailable, small molecule inhibitor of the 20S proteasome, and has shown single agent activity in phase I/II studies alongside combination therapy with dexamethasone and more recently IMiD agents and alkylating agents.[7-9] The oral formulation provides convenience

For peer review only - http://bmjopen.bmj.com/site/about/guidelines.xhtml

for patients, and the slower pharmacokinetic (PK) profile reduces the neurotoxicity seen with bortezomib, suggesting the use of this new PI in combination regimens might be better tolerated.[10] In the front-line setting the combination of ixazomib with lenalidomide and dexamethasone (IRD) has been reported in phase I/II studies demonstrating high response rates and good tolerability.[11]

With a favourable toxicity profile compared to either carfilzomib or bortezomib, and the benefits of oral dosing, the IRD combination represents a tolerable regimen to achieve the same combination of IMiD® agents and PI in TNE patients. The Tourmaline MM-1 study demonstrated excellent efficacy and tolerability of IRD in the relapsed setting in TNE patients, supporting this hypothesis.[12] The Tourmaline MM-02 study, a randomised, double blind, placebo controlled study evaluating IRd vs placebo Rd has recently been reported[13] and showed a clinically meaningful but non-significant 13.5 month improvement in PFS in the IRd group (35.3 vs 21.8 months; HR 0.83; P = 0.073). This trial used reactive dosing strategies.

Treatment Delivery

The International Myeloma Working Group (IMWG) proposed a scoring system for myeloma patient frailty that predicts survival, adverse events (AEs) and treatment tolerability,[14] which can help to account for the considerable heterogeneity in outcome for TNE patients. This score combines age and the outcomes of 3 patient assessment tools; the Katz Activity of Daily Living (ADL),[15] Lawton's Instrumental Activity of Daily Living (IADL),[16] and the Charlson Comorbidity Index (CCI)[17 18] to categorise patients into three groups: fit, unfit and frail. The IMWG frailty score was subsequently shown to be predictive of both PFS and toxicity. An increase in frailty score was associated with an increased risk of death, progression, non-haematological AEs and treatment discontinuation that was independent of classical definitions of risk, including ISS stage and cytogenetic risk, and also independent of treatment regimen. As such it was suggested to be useful in determining the feasibility of treatment regimens and appropriate dose reductions, but this remains to be prospectively validated.

Existing Evidence: Maintenance Therapy

Four published studies have demonstrated an important clinical benefit for the use of maintenance lenalidomide in newly diagnosed multiple myeloma in patients of all ages. Data from our previous study Myeloma XI contributes to this evidence base and in TNE patients, lenalidomide maintenance demonstrated a significant improvement in PFS compared to observation, of 24 months vs 11 months from maintenance randomisation.[19] This improvement was seen across all subgroups of multiple myeloma patients including all cytogenetic risk groups and at all ages. Overall survival data demonstrated a benefit for lenalidomide once the effect of subsequent therapies have been taken in to account.[20]

Overall, the data for maintenance lenalidomide until disease progression in patients not eligible for stem cell transplant suggest that there is a clear and significant improvement in PFS and a possible OS benefit. The crucial question to answer, going forward, is whether the results seen with lenalidomide as a single agent for maintenance can be enhanced further by the use of a combination regimen.

The use of ixazomib monotherapy in the maintenance setting demonstrated efficacy and tolerability in previously untreated patients [21] and this has recently been confirmed in the Phase III Tourmaline-MM4 trial in the non-transplant-eligible setting,[22] Adding ixazomib to lenalidomide maintenance has not been studied in a randomised phase III study.

For peer review only - http://bmjopen.bmj.com/site/about/guidelines.xhtml

Existing Evidence: Summary

The recently developed, less-toxic PI ixazomib [11] with novel IMiD® agents/PI combinations for induction and maintenance treatment needs to be evaluated in the context of the IMWG frailty score where frailty-adjusted dosing was recommended,[14] but emphasised the need for prospective validation of their approach.

Aims and Objectives

The FiTNEss trial aims to improve outcomes for TNE patients by investigating whether using prospective dose adjustments dependent on patient frailty will improve patients' ability to remain on therapy, reduce toxicity, and improve outcomes from randomisation 1 (R1). The trial also aims, from randomisation 2 (R2), to investigate whether doublet maintenance therapy improve outcomes compared to single agent lenalidomide without prohibitive toxicity.

Trial Design

The FiTNEss trial is a phase III, multi-centre, randomised, parallel group trial in newly diagnosed patients with MM, who are assessed to be TNE by their treating clinician. Following R2 the trial is also double-blind placebo-controlled with the participant and treating clinician blind to treatment allocation. The following report details the trial protocol and follows the structure of the SPIRIT statement.[23] The SPIRIT checklist[24] can be found within the supplementary material.

Methods

Setting

The trial will be conducted at 87 centres around the United Kingdom (See supplementary material), as identified via a feasibility assessment to determine the most appropriate to participate in the trial. The majority of potential participants will be identified by the research team at the time they are referred to the haematology outpatient department with suspected multiple myeloma. A smaller number of participants may be identified during inpatient admissions. Invitation to participate in the trial and provision of information will be made either during their first consultation, when routine diagnostic tests will be performed and potential treatment options discussed, or at the time they receive their diagnostic test results.

Eligibility criteria

Adults (18 years and older) with newly diagnosed MM, by IMWG 2014 diagnostic criteria, [25] who are TNE and who are capable of giving written informed consent will be assessed for eligibility. Eligibility will be confirmed prior to each randomisation by the principal investigator or authorised delegate and will be recorded in the participant's medical records and on the relevant case report form (CRF). The participant will be registered into the trial prior to undergoing procedures that are specifically for the purposes of the trial and are above NHS standard of care.

To be eligible for R1, participants must meet all the inclusion criteria and none of the exclusion criteria outlined in Table 1. Following 12 cycles of induction therapy, participants who achieve at least a minimal response (MR), according to IMWG uniform response criteria, and fulfill all the inclusion criteria and none of the exclusion criteria outlined in Table 2, will proceed to R2.

For peer review only - http://bmjopen.bmj.com/site/about/guidelines.xhtml

Table 1: Randomisation 1 Inclusion and Exclusion Criteria

**Inclusion Criteria:**
1. Newly diagnosed as having MM according to the updated IMWG diagnostic criteria 2014 requiring treatment.<br>2. Not eligible for stem cell transplant.<br>3. Aged at least 18 years.<br>4. Meet all of the following blood criteria within 14 days before R1:<br>Haematological:<br>a) Absolute neutrophil count (ANC) ≥ 1 x 109/L. Unless the participant has a known/suspected diagnosis of familial or racial neutropenia in which case an ANC ≥ 0.75 x 109/L is allowed. The use of growth factor support is permitted.<br>b) Platelet count ≥ 50 x 109/L, or, in the case of heavy bone marrow infiltration (≥ 50%) which in the opinion of the investigator is the cause of the thrombocytopenia and provided appropriate supportive measures and patient monitoring are in place, platelet count ≥ 30x 109/L is permitted. Please note: Platelet transfusions are not allowed ≤ 3 days prior to randomisation in order to meet these values.<br>c) Haemoglobin ≥ 80 g/L. The use of red blood cell transfusions is permitted.<br><br>Biochemical:<br>d) Total bilirubin ≤ 3 x upper limit of normal (ULN).<br>e) Alanine aminotransferase (ALT) and/or aspartate aminotransferase (AST) ⍰ 3 ⍰ ULN.<br>5. Meet the pregnancy prevention requirements:<br>Female participants who:<br>a) Are not of childbearing potential, OR<br>b) If they are of childbearing potential, agree to practice 2 effective methods of contraception, at the same time, from the time of signing the informed consent form until 90 days after the last dose of study drug, OR<br>c) Agree to practice true abstinence when this is in line with the preferred and usual lifestyle of the subject. (Periodic abstinence [e.g. calendar, ovulation, symptothermal, post-ovulation methods] and withdrawal are not acceptable methods of contraception.)<br><br>Male participants, even if surgically sterilised (i.e. status post-vasectomy), must agree to one of the following:<br>a) Agree to practice effective barrier contraception during the entire study treatment period and through 90 days after the last dose of study drug, OR<br>b) Agree to practice true abstinence when this is in line with the preferred and usual lifestyle of the subject. (Periodic abstinence (e.g. calendar, ovulation, symptothermal, post-ovulation methods] and withdrawal are not acceptable methods of contraception).<br><br>Contraception for female and male participants must be in accordance with (and participants must consent to) the Celgene-approved Pregnancy Prevention Programme.<br>If female and of childbearing potential, they must have a negative pregnancy test performed by a healthcare professional in accordance with the Celgene Pregnancy Prevention Programme.<br><br>6. Able to provide written informed consent.
**Exclusion Criteria:**
1. Smouldering MM, MGUS, solitary plasmacytoma of bone, or extramedullary plasmacytoma (without evidence of MM).<br>2. Received previous treatment for MM, with the exception of local radiotherapy to relieve bone pain or spinal cord compression, prior bisphosphonate treatment, or corticosteroids as long as the total dose does not exceed the equivalent of 160 mg dexamethasone.<br>3. Known resistance, intolerance or sensitivity to any component of the planned therapies.<br>4. Prior or concurrent invasive malignancies except the following:<br>- Adequately treated basal cell or squamous cell skin cancer;<br>- Incidental finding of low grade (Gleason 3+3 or less) prostate cancer requiring no intervention;<br>- Adequately treated carcinoma in situ of the breast or cervix no longer requiring medical or surgical intervention;<br>- Any cancer from which the subject has been disease-free for at least 3 years.<br>5. Pregnant, lactating or breastfeeding female participants.<br>6. Major surgery within 14 days before randomisation. This would include surgical intervention for relief of cord compression but does not include vertebroplasty or kyphoplasty.

For peer review only

For peer review only - http://bmjopen.bmj.com/site/about/guidelines.xhtml

7. Systemic treatment, within 14 days before the first dose of ixazomib with strong CYP3A inducers (e.g. rifampicin, rifabutin, carbamazepine, phenytoin, phenobarbital), or use of St. John's wort.

8. Any concomitant drug therapy which, in the opinion of the investigator, may lead to an unacceptable interaction with any of the agents ixazomib, lenalidomide, dexamethasone, and that cannot be safely stopped prior to trial entry. Full details of interactions can be found in the SPCs.

9. Known gastrointestinal (GI) disease or GI procedure that could interfere with the oral absorption or tolerance of trial treatment, including difficulty swallowing.

10. ≥ Grade 2 peripheral neuropathy.

11. Known HIV positive

12. Participant has current or prior hepatitis B surface antigen positive or hepatitis C antibody positive. Participants must have screening conducted within 14 days before R1.

13. Active systemic infection.

14. Any other medical or psychiatric condition which, in the opinion of the investigator, contraindicates the participant's participation in this study.

15. Receipt of live vaccination within 30 days prior to R1.

Table 2: Randomisation 2 Inclusion ad Exclusion Criteria

R2 Inclusion Criteria:

1. Randomised into the FiTNEss (Myeloma XIV) trial and received induction chemotherapy with ixazomib and lenalidomide continued for 12 cycles.

2. Achieved at least MR at the end of IRD induction according to the IMWG Uniform Response Criteria for Multiple Myeloma, with no evidence of progression prior to R2.

3. Meet all of the following blood criteria within 14 days before R2:
Haematological:
a) Absolute neutrophil count (ANC) ≥ 1 x 109/L. Unless the participant has a known/suspected diagnosis of familial or racial neutropenia in which case an ANC ≥ 0.75 x 109/L is allowed. The use of growth factor support is permitted.
b) Platelet count ≥ 50 x 109/L. Please note: Platelet transfusions are not allowed ≤ 3 days prior to randomisation in order to meet these values.
c) Haemoglobin ≥ 80 g/L. The use of red blood cell transfusions is permitted.

Biochemical:
d) Total bilirubin ≤ 3 x upper limit of normal (ULN).
e) Alanine aminotransferase (ALT) and/or aspartate aminotransferase (AST) ⍰ 3 ⍰ ULN.

R2 Exclusion Criteria:

1. Received any anti-myeloma therapy other than their randomised trial treatment, with the exception of local radiotherapy to relieve bone pain (in the absence of disease progression), or bisphosphonate treatment.

2. SD or disease progression according to the IMWG Uniform Response Criteria for Multiple Myeloma.

3. Known resistance, intolerance or sensitivity to ixazomib or lenalidomide that required cessation of either agent during induction.

4. Developed any malignancy since R1 except the following:

- Adequately treated basal cell or squamous cell skin cancer;
- Incidental finding of low grade (Gleason 3+3 or less) prostate cancer requiring no intervention;
- Adequately treated carcinoma in situ of the breast or cervix no longer requiring medical or surgical intervention.

5. Pregnant, lactating or breastfeeding female participants.

6. Major surgery within 14 days before randomisation. This does not include vertebroplasty or kyphoplasty.

7. Systemic treatment, within 14 days before the first dose of ixazomib with strong CYP3A inducers (e.g. rifampicin, rifabutin, carbamazepine, phenytoin, phenobarbital), or use of St. John's wort.

8. Known gastrointestinal (GI) disease or GI procedure that could interfere with the oral absorption or tolerance of trial treatment, including difficulty swallowing.

9. ≥ Grade 2 peripheral neuropathy, or grade 1 with pain.

10. Known HIV positive

For peer review only

For peer review only - http://bmjopen.bmj.com/site/about/guidelines.xhtml

11. Current or known hepatitis B surface antigen positive or hepatitis C antibody positive
12. Active systemic infection.
13. Any other medical or psychiatric condition which, in the opinion of the investigator, contraindicates the participant's continued participation in this study.
14. Receipt of live vaccination within 30 days prior to R1 or receipt of live vaccination at any point during the trial prior to R2.

Interventions and Dosing

Intervention Schedule

The control and experimental interventions for induction (R1) and maintenance (R2) therapy include: lenalidomide, ixazomib, placebo and dexamethasone. At R1, eligible patients will be allocated to one of two interventions with IRD induction; standard up-front dosing followed by toxicity dependent dose modification (reactive), or; frailty score-adjusted up-front dose reductions (adaptive). At R2, eligible patients will be randomised between lenalidomide + ixazomib (R+I) or lenalidomide and placebo maintenance therapy. Table 3 summarises the dosing schedules for R1 and R2. Information regarding dosing due to liver and renal function is provided in supplementary material.

Table 3: Dosing Schedule

Randomisation 1			
**Treatment**	**Induction – FIT + Induction – standard dosing**	**Induction- Unfit**	**Induction – Frail**
**Lenalidomide (Days = 1-21)**	25mg	15mg	10mg
**Ixazomib (Days = 1,8, 15) ***	4mg	4mg	4mg
**Dexamethasone **(Days = 1,8.15, 22)**	40mg in participants <= 75yrs<br>20 mg in participants >75yrs	20mg	10mg
**Randomisation 2**			
**Treatment**	**R+ Placebo Maintenance**	**R + I maintenance**	
**Lenalidomide (Days=1-21)**	10mg**	10mg**	
**Ixazomib (Days=1,8.15)***	N/A	4mg**	
**Placebo (Days=1,8, 15)**	4mg**	N/A	

* Ixazomib was not used in general multiple myeloma practice at the time of the EMN (European Myeloma Network) publication. The following licensed dose of Ixazomib will be at induction used for both randomisation arms: 4mg, at days 1, 8 and 15. This has been studied in TNE patients and was well tolerated. There have been no studies examining lower doses of Ixazomib so dose reductions are not permitted out of concern for loss of efficacy. The same dose is used irrespective of frailty.

**or final dose administrated at the end of induction treatment if lower

As part of their induction therapy, participants in the adaptive arm of R1 will have their dose adjusted according to changes in frailty category at the start of cycles 3, 5 and 7 (For the Myeloma Frailty index, participant's age is at the time of (Main) Trial registration, therefore, a patient's frailty will never change based on age only). Doses can also be escalated for suboptimal responders under certain criteria. If after cycle 2, a participant on the unfit or frail dosing strategy has not achieved at least an MR, or required a dose reduction due to toxicity, a request can be made to increase any of their doses to the next highest level at the start of cycle 4. A similar request can be made after cycle 4 for the start of cycle 6, but this requires at least a partial response (PR). This criteria applies for all participants irrespective of changes in frailty at cycles 3 and 5.

For peer review only - http://bmjopen.bmj.com/site/about/guidelines.xhtml

Intervention Adherence

Throughout the trial lenalidomide and ixazomib will be taken orally and swallowed whole at the same time on the scheduled days. Dexamethasone will be administrated in accordance with the relevant Summary of Product Characteristics (SPC). To monitor treatment adherence, participants will complete a daily medication diary which will be reviewed at trial visits. Unused capsules will be returned to pharmacy.

Dose Modification and Discontinuation

Both R1 and R2 treatment cycles will be 28 days in length. Response will be assessed at the end of each cycle according to the IMWG 2016 Uniform Response Criteria [26 27]. In the absence of progression or treatment intolerance, participants will receive a maximum of 12 cycles of induction therapy and continuous maintenance therapy. Those who receive 12 cycles of induction therapy will be assessed for R2 eligibility, with non-eligible participants being treated off trial.

Toxicity and hence treatment intolerance will be assessed throughout each treatment cycle, according to the National Cancer Institute (NCI) common terminology criteria for AE (CTCAE) Version 5.

For a new cycle of treatment to begin (induction and maintenance), the participant must meet the haematological and biochemical criteria (at day 1 or ≤3 days prior) outlined in the eligibility criteria (R1: Table 1, R2: Table 2). Non-haematological toxicities (except for alopecia) must have resolved to less than or equal to Grade 1 or to the participants baseline condition in order for treatment to resume. If the participant does not meet this criteria their dose will be delayed for one week before they are re-assessed. This will continue for a maximum of three weeks before treatment discontinuation or eight weeks at the discretion of the Chief Investigator.

In the event that a dose is reduced due to toxicity, as per the lenalidomide SPC the dose may be reintroduced to the next higher dose level upon improvement. Ixazomib, once reduced cannot be re-escalated.

Concomitant medication

Concomitant medication, disease and other malignancies will be recorded at eligibility.

All participants may receive additional care during the treatment period as deemed appropriate by the treating clinician. Local support care protocols, including anti-emetic schedules, tumor lysis syndrome prevention, VTE (Venous thromboembolism) prophylaxis and prophylactic antimicrobial therapy, will be followed for both randomisations. Permitted and excluded concomitant medications and procedures can be found in the supplementary material.

Outcomes

Primary outcome

For R1 the primary outcome is early treatment cessation (defined at a binary endpoint) for reactive versus adaptive dosing in participants defined to be “unfit” or “frail” at baseline. Participants will be defined to have experienced an event if they die, progress, or are withdrawn from treatment (by the treating clinician) or withdraw consent for treatment, within 60 days of R1.

For R2 the primary outcome is PFS for R+Placebo versus R+I, and is defined as the time from R2 to the time of first documented disease progression or death from any cause. Individuals lost to follow up or

For peer review only - http://bmjopen.bmj.com/site/about/guidelines.xhtml

progression-free at time of analysis will be censored at their last known alive and progression-free date.

Secondary outcomes

The secondary outcomes of this trial are to assess PFS for reactive versus adaptive dosing, time to progression, time to 2nd PFS event (PFS2), overall survival (OS), survival after progression, deaths within 12 months of R1, overall response rate (ORR), attainment of ≥VGPR, attainment of MRD negativity (Flow MRD will be assessed only. Additional detail on the testing of MRD and time points is presented in the 'Central Lab analysis' section of the supplementary material), duration of response, time to improved response, time to next treatment, treatment compliance and total amount of therapy delivered, toxicity and safety including the incidence of Second Primary Malignancies (SPMs), Quality of Life (QoL), cost-effectiveness of reactive versus adaptive dosing of IRD and cost-effectiveness of R+I versus R.

Exploratory outcomes

Exploratory outcomes are to prospectively validate the UK-MRA Myeloma Risk Profile, to assess the usefulness of the Karnofsky performance status (PS), and consider the association of molecular subgroups with response, PFS and OS.

Participants Timelines

The full trial schema can be seen in Figure 3. The schedule of assessments at each time point are presented in Figure 4.

Trial Entry

Participants will enter the trial at one of two points in their patient pathway, this will either at bone marrow registration or main trial registration.

Trial consent

Participants who enter the trial at bone marrow registration will provide consent to having bone marrow and blood samples taken and sent to central laboratories for analysis. If the participant is diagnosed with a plasma cell dyscrasia, other than myeloma, or they have myeloma, but decide not to take part in the FiTNEss trial, they will also have the option of consenting to their samples being used in future research.

All participants, will provide written informed consent for the trial prior to trial registration. Optional consent for QoL resources and the use of samples for future research will also be obtained.

Trial Registration

Following trial consent participants will be registered onto the main trial and assessed for eligibility. Consenting patients will complete the baseline QoL and Healthcare resource use questionnaires. Trial samples for blood and urine will be taken for all participants and bone marrow samples will be taken for those who did not enter the trial through bone marrow registration.

Trial Treatment

Following trial registration participants will be randomised into R1 and treated as described in the intervention schedule on a monthly basis. In absence of disease progression or intolerance, those participants with at least an MR following 12 cycles of maintenance and whom meet all of the R2 eligibility criteria will proceed to R2 maintenance treatment.

For peer review only - http://bmjopen.bmj.com/site/about/guidelines.xhtml

Participants will be followed up monthly (at each cycle) whilst receiving maintenance treatment, until death or until the final analysis of the trial (whichever happens sooner).

Trial Follow-up

Participants who discontinue treatment during induction and before the point of R2 will be followed up to the point of assessment for eligibility for R2, unless they withdraw consent for this. Thereafter, participants will continue to be followed-up for data pertaining to safety, progression (including second progression), and survival. Frailty scores will be completed for all participants at 2, 4, 6 and 12 months post-R1, irrespective of whether they have discontinued treatment. Similarly, QoL and Healthcare resources use questionnaires (if the participant has consented to these) will continue to be completed at 2, 6 and 12 months post-R1.

If treatment has been stopped without progression following R2, e.g. due to toxicity, then participants will be followed up two monthly until disease progression. Follow-up will include local investigations, central investigations, frailty index at 6 and 12 months post-R2 and QoL and Healthcare resource use at 6 and 12 months post-R2 (if consented).

Sample Size

In total 740 participants will be enrolled into the trial at R1 to ensure that at least 478 participants remain on trial and are randomised to R2. It is assumed that 65% of those randomised at R1 will be progression-free and, therefore, eligible for R2, hence 740 participants are required to be enrolled.

Based on data from the Myeloma XI non-intensive pathway we hypothesise the frail and unfit patients in R1 will be similar to the older patients in Myeloma XI (>75 years) who have an early treatment cessation rate (within 60 days of R1) of 20%. This hypothesis is based on the expectation that the frailty score is heavily driven by patient age. Younger patients (≤75 years) in the Myeloma XI non-intensive pathway have an early treatment cessation rate of 9% and it is our hypothesis that our frailty-based dosing schedule has the potential to reduce the rate amongst the unfit and frail patients to the proportion observed in fit patients [28 29] .

To demonstrate a decrease of 11 percent points in the proportion of early treatment cessation from 20% in the standard dosing schedule arm to 9% in the frailty score-adjusted dose arm amongst those patients scored at baseline to be unfit or frail would require the recruitment of 324 patients with an allocation ratio of 1:1. These calculations are based on a Pearson's chi-squared test without continuity correction, assume a 2-sided 5% level of significance, 80% power, and allow for a 1% dropout prior to 60 days post-randomisation. Given that we anticipate that 45% of patients will be scored as unfit or frail, by assuming this trial will have a similar underlying population as in Myeloma XI non-intensive pathway and the age distributions in the IMWG report proposing the frailty score [14], we would anticipate that we will require 720 patients to enter the trial at R1 to have sufficient unfit and frail patients available to answer this question.

For R2, in the non-intensive pathway of Myeloma XI, the median PFS for patients on R following CRDa induction was approximately 33 months [19], where approximately 65% of individuals were progression-free 12 months post-randomisation. As R2 is approximately 12 months following R1 in FiTNEss we assume that the median PFS for patients receiving R maintenance therapy will be 21 months from R2. Tourmaline-MM1 [12] demonstrated a hazard ratio for PFS of 0.74 when comparing IRD and RD in patients with relapsed and refractory multiple myeloma. Thus a similar hazard ratio would be the minimum clinically relevant difference for our comparison in patients with newly diagnosed multiple myeloma. Assuming a median PFS of 29 months for those in the R+I maintenance group in addition to the assumption of 21 months in the R group equates to a hazard ratio of 0.72.

For peer review only - http://bmjopen.bmj.com/site/about/guidelines.xhtml

The above assumptions require the recruitment of 478 participants over a 30 month recruitment period with a further 24 months of follow-up. These calculations also assume a 2-sided 5% significance level, 80% power and allow for a 3% dropout rate prior to a PFS event being experienced. Note that 80% power is attained when 302 events have been observed. A total of 740 participants should be allocated to R1 to ensure that 478 participants are available at the second randomisation.

Overall survival is considered to be a key secondary endpoint for R2, 180 events with a minimum of 2 years follow up for all participants are required for 80% power.

Recruitment

It is planned that 740 participants will be recruited over a 30 month recruitment period from 87 UK centres. Once all centres are open, the recruitment target is 30 participants a month. The trial opened to recruitment on the 4th August 2020. As of May 2021 85 sites are open to recruitment and 137 participants have been randomised to the first stage of the trial.

In order to ensure the trial will meet the target sample size within the recruitment period, site set-up was prioritised whilst the trial was preparing to open to recruitment. In addition, the trial team are actively engaging with Principal Investigators at sites who support the trial to ensure that those sites open quickly and are maintaining regular communication with open sites to ensure that they continue to recruit to the trial. Finally, whilst the trial was originally delayed due to the COVID-19 pandemic, efforts were made to ensure that it opened as soon as possible when research restarted. One factor being the positive risk to benefit ratio of the number of hospital visits required for the trial interventions as compared to standard of care.

Assignment of treatment allocations

Each of the registration and randomisation procedures will be conducted centrally using the Leeds CTRU (Clinical Trial Research Unit) automated 24 hour web based and telephone system.

Registrations

If a patient is suspected to have myeloma they will enter the trial at the time of routine diagnostic tests, through bone marrow registration. Once diagnosis is confirmed locally and the research team consider the patient potentially eligible for the trial, patients will be provided with full verbal explanation of the trial and the full participant information sheet and informed consent documents to consider. Once the participant has provided informed consent, they will be registered onto the trial.

Other participants who have myeloma confirmed prior to entering trial, will enter at trial registration.

Randomisations

Following trial registration patients will be assessed for R1 eligibility. Eligible participants will be randomised on a 1:1 basis into R1, using the stratification factors; centre, IMWG frailty category, Beta-2 microglobulin concentration (<3.5, 3.5-<5.5, $\geq$5.5 mg/L), Haemaglobulin concentration (<100, $\geq 100g/L$), serum crestinine concentration (<175, $\geq$ 175 µmol/L), corrected serum calcium concentration (<2.75, $\geq$ 2.75mmol/L), platelets (<150, $\geq$ 150 x $10^9/L$).

Following R1, and as soon as the end of induction response is known, R2 eligible participants will be randomised on a 1:1 basis into R2, using the stratification factors; centre, allocated induction arm (reactive, adaptive); final response to induction treatment (<VGPR, $\geq$ VGPR).

For peer review only - http://bmjopen.bmj.com/site/about/guidelines.xhtml

For both R1 and R2, a computer generated minimization program that incorporates a random element will be used to ensure treatment groups are well balanced for the specified stratification factors.

Blinding Methods

For R2 treatment allocation will be concealed from participants, treatment provider and the trial team. The placebo and ixazomib capsules will be identical in colour, size, packaging and labelling.

To maintain the overall integrity of the trial design, unblinding will only be permitted in exceptional circumstances; for example valid medical or safety reasons where assuming that the patient is receiving active treatment and/ or stopping the blinded medication is insufficient.

Unblinding will be conducted automatically using an online system accessed by an authorised member of the site research team.

If unblinding is performed at any stage during the trial, decisions around further trial treatment will be the responsibility of the principal investigator or delegate. In either case unblinded participants will be followed up as per the protocol.

At the completion of the trial and after final analysis, participants will be given the opportunity to be informed of their allocation by their research site.

Data Collection

Clinical data will be collected both electronically and on paper by staff at research site completing CRFs provided by CTRU. QoL and Healthcare Resource Use questionnaires will be completed on paper CRFs by the participant. All paper CRFs will be sent to CTRU, by the research site, usually via standard post and entered onto an electronic database. This data along with the data entered electronically by staff at each research sites will be validated and monitored for completeness and quality by the CTRU.

Missing data will be chased until it is received, confirmed as not available or the trial is at analysis. Missing QoL data items will not be chased from participants, although missing questionnaires may be chased from sites.

Data management

Validation checks will be incorporated into the trial database to verify the data, and discrepancy reports will be generated for resolution by the trial site. Priority validations will be incorporated to ensure that any discrepancies related to participant rights, or the safety of participants, are expedited to sites for resolution. The CTRU/Sponsor will reserve the right to intermittently conduct source data verification exercises on a sample of participants, which will be carried out by staff from the CTRU/Sponsor. Source data verification will involve direct access to participant notes at the participating hospital sites and the ongoing central collection of copies of consent forms and other relevant investigation reports.

Statistical methods

Statistical analysis is the responsibility of the CTRU statisticians, with the exception of the analysis for cost-effectiveness of delivery of IRD and R/R+I, which will be undertaken by Health Economists at the University of Leeds. A full statistical analysis plan (SAP) and Health Economics Analysis Plan (HEAP) will be written and approved before any analysis is undertaken.

All analyses will be conducted on the intention-to-treat (ITT) population, where participants will be included according to their randomisation allocation regardless of eligibility, whether they

For peer review only - http://bmjopen.bmj.com/site/about/guidelines.xhtml

prematurely discontinued treatment, or did not comply with the regimen. A per-protocol (PP) analysis, where participants will be included if they received their allocated intervention according to the protocol, will be considered for the primary endpoints if there are a considerable number of major protocol violators. The safety population will consist of all participants who received at least one dose of the trial treatment and participants will be summarised as per their treatment received rather than their allocation.

An overall two-sided 5% significance level will be used for all efficacy endpoint comparisons. For the primary endpoints, this will be adjusted to account for the formal interim analyses.

Primary Endpoint Analysis

Randomisation 1

For R1, the number and proportion of participants, categorised as unfit or frail at baseline, experiencing an early treatment cessation event will be summarised by randomisation allocation and exact 95% confidence intervals will be calculated.

A logistic regression model will regress early treatment cessation on randomisation allocation (reactive / adaptive dosing) adjusting for the stratification factors of the trial. A statistically significant induction treatment effect will be suggested if the p-value for the resulting odds ratio is <0.047. Parameter estimates, odds ratios and corresponding 95% confidence intervals, degrees of freedom, test statistics and p-values will be presented for each variable in the model. Residuals and predicted values produced from the models will be examined to assess the assumptions of the statistical models.

Randomisation 2

For R2, PFS between the two maintenance therapies (R+Placebo / R+I) will be compared using a Cox regression model adjusting for the stratification factors of the trial. A statistically significant maintenance treatment effect will be suggested if the p-value for the hazard ratio corresponding to randomisation allocation is <0.047. Parameter estimates, hazard ratios and corresponding 95% confidence intervals, degrees of freedom, test statistics and p-values will be presented for each variable in the model.

The proportional hazards assumptions will be assessed by plotting the hazards over time (i.e. the log cumulative hazard plot) for each treatment arm and using appropriate statistical tests. If evidence is found to support the violation of the proportional hazards assumption then alternative appropriate analysis methods will be investigated.

No imputation strategy is planned for the primary endpoints.

Secondary Endpoint Analysis

Secondary endpoint analysis of OS and other time-to-event endpoints will be analysed using similar methods to those described for PFS.

MRD negativity and other binary endpoints will be analysed using similar methods to those described for the early treatment cessation primary endpoint. The number and proportion of participants in each response category (sCR, CR, VGPR, etc.) will be summarised by allocated treatment and exact

For peer review only - http://bmjopen.bmj.com/site/about/guidelines.xhtml

95% confidence intervals will be calculated. The difference in proportions for each response category will be presented with corresponding 95% confidence intervals.

The domains of the QoL questionnaires will be summarised using mean scores adjusted for baseline and 95% CIs at each assessment time-point. Similar summaries will be produced for quality-adjusted life years (QALYs) derived using the EQ-5D-3L questionnaire.

Exploratory and subgroup analyses

An overview of the planned exploratory and subgroup analysis can be found in the supplementary material. These include genetic and molecular analysis of patient samples conducted by the respective central laboratories.

Health economics

A full HEAP will be written and approved before any analysis is undertaken.

Economic evaluations will be conducted at R1 and R2, using within-trial and decision-model based analyses. The analysis will be guided by the NICE reference case, applying the cost-utility framework from the perspective of the health and social care provider over a life-time horizon. Base case QALYs will be based on EQ-5D-3L responses, and costs on patient completed resource use forms and hospital records. Results will be presented in terms of incremental cost-effectiveness ratios, cost-effectiveness acceptability frontiers and net benefit.

Trial Oversight

The trial management group (TMG), comprises of the Chief and Co-Chief Investigators, CTRU team and co-investigators and are responsible for the clinical set up, ongoing management, promotion of the trial and for the interpretation of the results. The trial steering committee (TSC), consisting of independent clinicians and statisticians, along with a patient representative, will provide overall supervision on the trial, including trial progress, adherence to protocol, participant safety and consideration of new information.

Data monitoring

An independent data monitoring and ethics committee (DMEC) will review the safety and ethics of the trial by reviewing unblinded interim data prepared by the CTRU in strict confidence at approximately yearly intervals. Unblinded safety updates are also prepared at 6 monthly intervals. After each annual review of safety data, the DMEC will make their recommendations to the TSC about the continuation of the trial who will make their recommendations known to the TMG.

Interim Analyses

Two formal interim analyses will be undertaken for early efficacy, one for each of the randomisations. The first will occur when 50% of required participants (370 participants) have reached 60 days post-R1. The second will occur when 50% of required PFS events have been observed (151 events) following R2. In order to maintain an overall two-sided 5% significance level for the primary endpoint analysis the O'Brien and Fleming alpha spending function [30] will be used. This results in a 0.05% significance level for the interim analysis. The analysis itself will reflect that detailed in the statistical analysis section. For the second interim analysis, only the DMEC, safety statistician and supervising statistician will see the unblinded results, as is standard procedure for double blind trials.

For peer review only - http://bmjopen.bmj.com/site/about/guidelines.xhtml

No other formal analysis of the trial is planned before the participants have attained the primary endpoints.

The DMEC, in the light of the interim data, and any advice or evidence they wish to request, will advise the TSC if there is proof beyond reasonable doubt that one treatment is better and recommend appropriate changes to the trial protocol.

Harms

Adverse Events

Adverse Events (AEs) are any untoward medical occurrence in a patient or clinical trial subject administrated a medicinal product and which does not necessarily have a causal relationship with this treatment. AEs can be defined as any unfavourable and unintended sign (including an abnormal laboratory finding, for example), symptom or disease. Due to the nature of myeloma and its treatment, participants are likely to experience several AEs throughout the course of the disease.

All AEs, both related and unrelated to myeloma treatment will be collected on the relevant CRF from R1 until 60 days after the last dose of protocol treatment and will be evaluated and summarised in accordance with the NCI-CTCAE V5.

Serious Adverse Events

Serious Adverse Events (SAEs) are defined as any untoward medical occurrences or effects that at any dose result in death; or are life threatening (at the time of event); or require in patient hospitalisation or prolongation of existing hospitalisation; or result in persistent or significant disability of incapacity; or result in a congenital abnormality or birth defect; or jeopardise the participant or may require an intervention to prevent one of the above outcomes/consequences (other important medical event). SAEs will be reported from R1 until 60 days post the last dose of trial drug.

Serious Adverse Reactions (SARs) are SAEs that are deemed to be possibly related to any dose administrated of any trial treatment. Suspected unexpected serious adverse reactions (SUSARs) are SARs, of which the nature and severity is not consistent with the applicable reference safety information. SUSARs and SARs will be reported from the date of the first trial drug for the duration of the trial.

Presenting Safety Data

Safety analyses will summarise all SUSARs, SARs, SAEs, ARs, AEs and treatment-related mortality rates. Safety data will be presented by treatment group for the safety population in addition to suspected relationship to trial treatment.

Secondary Primary Malignancies

All new secondary primary malignancies (SPMs) or suspected malignancies will be recorded from R1 for the duration of the trial and will be summarised and reviewed by an appointed member of the TMG, who will determine whether trial treatment should continue.

Pregnancies

For peer review only - http://bmjopen.bmj.com/site/about/guidelines.xhtml

The Celgene approved pregnancy programme will be followed as per usual clinical practice. Pregnancies in participants on trial treatment will be prevented as effectively as possible. Pregnancies and suspected pregnancies in a female or male participant's partner occurring at any time until 90 days post cessation of trial treatment will be reported.

Auditing

The CTRU and the trial Sponsor have procedures in place to ensure that serious breaches of GCP or the trial protocol are identified and reported. A triggered monitoring plan will ensure that sites at risk are monitored accordingly.

Patient and public involvement

FiTNEss has been developed following extensive discussion within the UK myeloma community, including with the NCRI Myeloma Subgroup (the UK Myeloma Research Alliance (UK-MRA)) and the NCRI Haematological Oncology Group. Both groups include patient and public representatives who work with clinical members of the group. To develop studies which address key questions for induction and maintenance.The protocol was reviewed in depth by a patient representative in order to ensure that the interventions and proposed schedule of assessments would be acceptable to patients. In addition, the trial consent and participant information document was reviewed for clarity by the same patient representative. Furthermore, to ensure that the patient perspective is considered throughout the trial, a patient representative sits on the TSC.

Ethics and Dissemination

Ethics approval statement

Ethical approval has been obtained from the North East - Tyne & Wear South Research Ethics Committee (reference 19/NE/0125). In addition approval was granted by the appropriate local research and development department for each participating centre prior to opening to recruitment. Participants will be required to provide written informed consent before joining the trial.

Protocol Amendments

The trial opened to recruitment on 4th August 2020 using protocol version 2, dated 10th October 2019. An amendment to protocol version 3 is anticipated in June 2021, which will include the addition of the secondary endpoint Event-Free Survival, clarification on the requirements around when face-to-face assessments should be conducted, and adding in the recommendation of 5 mg once daily of lenalidomide for participants with severe renal impairment, as opposed to 15mg every other day.

Consent

The Principal Investigator retains overall responsibility for the informed consent of participants at their site and must ensure that any medically qualified person delegated responsibility to participate in the informed consent process is duly authorized, trained and competent to participate according to ethical approved protocol, principles of Good Clinical Practice (GCP) and Declaration of Helsinki. Written consent will be obtained and signed by a medically qualified member of the site research team. A record of the consent process for both bone marrow and blood sample consent and full trial consent, including the date of consent and all those present, will be kept in the participant's notes. At any stage, participants can withdraw consent without repercussion.

Confidentiality

For peer review only - http://bmjopen.bmj.com/site/about/guidelines.xhtml

All information collected during the course of the trial will be kept strictly confidential. Information will be held securely on paper at Leeds CTRU. In addition, the CTRU will hold electronic information on all trial participants. The CTRU will have access to the entire database for monitoring, coordination, and analysis purposes.

Access to data

Data will not be made available until the end of the study, The CTRU will control the final trial datasets, and any requests for data will be reviewed by the trial management group in the first instance. Only requests that have a methodologically sound proposal and whose proposed use of the data has been approved by the independent trial steering committee, based on the principles of a controlled access approach, and subject to existing contractual agreements with the funder, will be considered. Proposals should be directed to Leeds Clinical Trials unit *(*CTRU-DataAccess@leeds.ac.uk) in the first instance; to gain access, data requestors will need to sign a data access agreement.

Ancillary and post-trial care

Participants who stop trial treatment due to progression or any point prior to the end of trial will be treated off-trial at the discretion of their treating clinician. Following disease progression, participants will be followed up annually until death, or until the end of the trial for post-progression endpoints.

Dissemination policy

Authorship of clinical and translational outputs will be in keeping with the UK-MRA Publication Policy and due acknowledgement to participants, local investigators, funders and NCRI Haematological-Oncology Group support made. The success of the trial depends upon the collaboration of all trial members. For this reason, credit for the main results will be given to all those who have collaborated in the trial, through authorship and contributorship. Uniform requirements for authorship for manuscripts submitted to medical journals will guide authorship decisions alongside the guidance of the UK-MRA.

To maintain the scientific integrity of the trial, data will not be released prior to the end of the trial or a primary endpoint being reached, either for trial publication or oral presentation purposes, without the permission of the Trial Steering Committee and the (Co-)Chief Investigators. In addition, individual collaborators must not publish data concerning their participants that is directly relevant to the questions posed in the trial until the main results of the trial have been published and following written consent from the Sponsor.

Appendices

Informed consent material

The consent forms which are to be completed by the participant at Bone Marrow Registration and/or Trial Registration are included in supplementary material.

Biological specimens

The collection of central samples for laboratory analysis is summarised in Figure 4. The analysis to be conducted for trial purposes is stated in the supplementary material. Additional analysis may be carried out by each central laboratory provided the appropriate consent for sample use in future research has been provided by the participant at trial entry.

<u>**Author Contributions**</u>

For peer review only - http://bmjopen.bmj.com/site/about/guidelines.xhtml

The FiTNEss trial was conceived by GC and GJ and designed by them in collaboration with CPaw, DAC, AH, JB, SB, MK, RO, MJ, BK, and MD. All authors (ABC, KLR, CPaw, DAC, AH, JB, SB, MK, RT, NR, KB, JJ, CP, HG, DM, BD, CO, RH, PB, RO, MJ, BK, MD, GJ and GC) inputted into the development of the protocol and patient information sheet. The first draft of the manuscript was written by ABC and KLR. All authors (ABC, KLR, CPaw, DAC, AH, JB, SB, MK, RT, NR, KB, JJ, CP, HG, DM, BD, CO, RH, PB, RO, MJ, BK, MD, GJ and GC) reviewed and approved the final manuscript.

Funding

This trial is funded by Cancer Research UK (C37712/A21282) with further unrestricted funding from Millennium: The Takeda Oncology Company and Celgene: A Bristol Myers Squibb Company. This work was also supported by Core Clinical Trials Unit Infrastructure from Cancer Research UK (C7852/A25447). The funders had no role in the design, collection, analysis or collection of data; in writing the manuscript; or in the decision to submit the manuscript for publication.

The trial Sponsor is responsible for the overall conduct of the trial as defined by Directive 2001/20/EC is University of Leeds, UoL/LTHT Joint Sponsor QA office (CTIMPs); Research & Innovation Centre/Faculty of Medicine & Health; Leeds Teaching Hospitals NHS Trust/University of Leeds; St James University Hospital; Leeds LS9 7TF.

Competing Interest Statement

ABC, KLR, DAC, AH, CO, RH, PB report grants and non-financial support from BMS/Celgene, grants and non-financial support from Merck Sharpe & Dohme, grants and non-financial support from Amgen, grants and non-financial support from Takeda, during the conduct of the trial. DAC also reports travel support from Celgene Corporation. Cpaw reports receiving honoraria and/or travel support from Amgen, BMS/Celgene, Janssen, Sanofi and Takeda. MK consultancy: AbbVie, Amgen, BMS/Celgene, GSK, Janssen, Karyopharm, Seattle Genetics, Takeda; honoraria: BMS/Celgene, Janssen, Takeda; Research funding: BMS/Celgene; Travel/educational support: BMS/Celgene, Janssen, Takeda. SB reports receiving research funding from Takeda. KB – Advisory Boards Janssen: BMS/Celgene, Takeda, Novartis. Speaker Honoraria: Janssen, BMS/Celgene, Sanofi, Takeda. Support to attend educational meetings: Janssen, BMS/Celgene, Takeda, GSK.GJ reports research funding from Takeda, Onyx, MSD & BMS/Celgene with consultancy from Janssen, Takeda, Sanofi, Oncopeptides, Karyopharm, Pfizer, Roche & BMS/Celgene. GC reports research funding from Janssen, Takeda, Amgen & BMS/Celgene with consultancy from Janssen, Takeda, Sanofi, Oncopeptides, Karyopharm, Pfizer, Roche & BMS/Celgene .MD reports Stock held in Abingdon Health LtdJB, RDT, NR, JJ, CPar, HG, DM, BD, RO, MJ and BK have no declared competing interests.

For peer review only - http://bmjopen.bmj.com/site/about/guidelines.xhtml

References

1. CANCER RESEARCH UK. Myeloma Incidence Statistics [online]: CANCER RESEARCH UK; 2015 [Available from: http://www.cancerresearchuk.org/health-professional/cancer-statistics/statistics-by-cancer-type/myeloma/incidence accessed June.
2. Morgan GJ, Davies FE, Gregory WM, et al. Long-term Follow-up of MRC Myeloma IX Trial: Survival Outcomes with Bisphosphonate and Thalidomide Treatment. *Clinical cancer research* 2013;19(21):6030-38. doi: 10.1158/1078-0432.CCR-12-3211
3. Pawlyn C, Cairns D, Kaiser M, et al. The relative importance of factors predicting outcome for myeloma patients at different ages: results from 3894 patients in the Myeloma XI trial. 2020
4. Jackson GH, Davies FE, Pawlyn C, et al. Response-adapted intensification with cyclophosphamide, bortezomib, and dexamethasone versus no intensification in patients with newly diagnosed multiple myeloma (Myeloma XI): a multicentre, open-label, randomised, phase 3 trial. *The Lancet Haematology* 2019;6(12):e616-e29. doi: 10.1016/S2352-3026(19)30167-X
5. Pawlyn C, Davies FE, Cairns DA, et al. Quadruplet Vs Sequential Triplet Induction Therapy Approaches to Maximise Response for Newly Diagnosed, Transplant Eligible, Myeloma Patients. *Blood* 2015;126(189)
6. Jackson GH, Pawlyn C, Cairns DA, et al. Carfilzomib, lenalidomide, dexamethasone, and cyclophosphamide (KRdc) as induction therapy for transplant-eligible, newly diagnosed multiple myeloma patients (Myeloma XI+): Interim analysis of an open-label randomised controlled trial. *PLoS medicine* 2021;18(1):e1003454-e54. doi: 10.1371/journal.pmed.1003454
7. Richardson PG, Baz R, Wang M, et al. Phase 1 study of twice-weekly ixazomib, an oral proteasome inhibitor, in relapsed/refractory multiple myeloma patients. *Blood* 2014;124(7):1038-46. doi: 10.1182/blood-2014-01-548826
8. Offidani M, Corvatta L, Caraffa P, et al. An evidence-based review of ixazomib citrate and its potential in the treatment of newly diagnosed multiple myeloma. *OncoTargets and therapy* 2014;7:1793-800. doi: 10.2147/OTT.S49187
9. Assouline SE, Chang J, Cheson BD, et al. Phase 1 dose-escalation study of IV ixazomib, an investigational proteasome inhibitor, in patients with relapsed/refractory lymphoma. *Blood cancer journal (New York)* 2014;4(10):e251-e51. doi: 10.1038/bcj.2014.71
10. Gupta N, Zhao Y, Hui AM, et al. Switching from body surface area-based to fixed dosing for the investigational proteasome inhibitor ixazomib: a population pharmacokinetic analysis. *British journal of clinical pharmacology* 2015;79(5):789-800. doi: 10.1111/bcp.12542
11. Kumar SK, Berdeja JG, Niesvizky R, et al. Safety and tolerability of ixazomib, an oral proteasome inhibitor, in combination with lenalidomide and dexamethasone in patients with previously untreated multiple myeloma: an open-label phase 1/2 study. *The Lancet Oncology* 2014;15(13):1503-12.
12. Moreau P, Masszi T, Grzasko N, et al. Ixazomib, an investigational oral proteasome inhibitor (PI), in combination with lenalidomide and dexamethasone (IRd), significantly extends progression-free survival (PFS) for patients (Pts) with relapsed and/or refractory multiple myeloma (RRMM): the phase 3 Tourmaline-MM1 study (NCT01564537): Am Soc Hematology, 2015.
13. Facon T, Venner CP, Bahlis NJ, et al. MM-347: Ixazomib Plus Lenalidomide-Dexamethasone (IRd) vs. Placebo-Rd for Newly Diagnosed Multiple Myeloma (NDMM) Patients Not Eligible for Autologous Stem Cell Transplant: The Double-Blind, Placebo-Controlled, Phase 3 TOURMALINE-MM2 Trial. *Clinical lymphoma, myeloma and leukemia* 2020;20:S307-S08. doi: 10.1016/S2152-2650(20)30955-1
14. Palumbo A, Bringhen S, Mateos M-V, et al. Geriatric assessment predicts survival and toxicities in elderly myeloma patients: an International Myeloma Working Group report. *Blood* 2015;125(13):2068-74.

For peer review only - http://bmjopen.bmj.com/site/about/guidelines.xhtml

15. Katz S, Ford AB, Moskowitz RW, et al. Studies of illness in the aged: The index of adl: a standardized measure of biological and psychosocial function. *JAMA* 1963;185(12):914-19. doi: 10.1001/jama.1963.03060120024016
16. Lawton MP, Brody EM. Assessment of older people: self-maintaining and instrumental activities of daily living. *The gerontologist* 1969;9(3_Part_1):179-86.
17. Charlson ME, Pompei P, Ales KL, et al. A new method of classifying prognostic comorbidity in longitudinal studies: development and validation. *Journal of chronic diseases* 1987;40(5):373-83.
18. Delforge M, Minuk L, Eisenmann J-C, et al. Health-related quality-of-life in patients with newly diagnosed multiple myeloma in the FIRST trial: lenalidomide plus low-dose dexamethasone versus melphalan, prednisone, thalidomide. *Haematologica* 2015;100(6):826-33. doi: 10.3324/haematol.2014.120121
19. Jackson GH, Davies FE, Pawlyn C, et al. Lenalidomide Is a Highly Effective Maintenance Therapy in Myeloma Patients of All Ages; Results of the Phase III Myeloma XI Study. *Blood* 2016;128(22):1143-43.
20. Jackson G, Davies FE, Pawlyn C, et al. Lenalidomide Maintenance Significantly Improves Outcomes Compared to Observation Irrespective of Cytogenetic Risk: Results of the Myeloma XI Trial. *Blood* 2017;130(Supplement 1):436-36. doi: 10.1182/blood.V130.Suppl_1.436.436 %J Blood
21. Kumar S, Berdeja JG, Niesvizky R, et al. Long-Term Ixazomib Maintenance Is Tolerable and Improves Depth of Response Following Ixazomib-Lenalidomide-Dexamethasone Induction in Patients (Pts) with Previously Untreated Multiple Myeloma (MM): Phase 2 Study Results. *Blood* 2014;124(21):82-82.
22. Dimopoulos MA, Spicka I, Quach H, et al. Ixazomib as Postinduction Maintenance for Patients With Newly Diagnosed Multiple Myeloma Not Undergoing Autologous Stem Cell Transplantation: The Phase III TOURMALINE-MM4 Trial. *Journal of clinical oncology* 2020;38(34):4030-41. doi: 10.1200/JCO.20.02060
23. Chan A-W, Tetzlaff JM, Altman DG, et al. SPIRIT 2013 Statement: Defining Standard Protocol Items for Clinical Trials. *Annals of internal medicine* 2013;158(3):200-07. doi: 10.7326/0003-4819-158-3-201302050-00583
24. Chan A-W, Tetzlaff JM, Gøtzsche PC, et al. SPIRIT 2013 explanation and elaboration: guidance for protocols of clinical trials. *BMJ (Online)* 2013;346(jan08 15):907-e7586. doi: 10.1136/bmj.e7586
25. The International Myeloma Working Group (IMWG). updated criteria for the diagnosis of multiple myeloma. *The Lancet 2014*;15 (12):538-48 (43).
26. Rajkumar SV, Harousseau JL, Durie B, et al. Consensus recommendations for the uniform reporting of clinical trials: report of the International Myeloma Workshop Consensus Panel 1. *Blood* 2011;117(18):4691-5. doi: 10.1182/blood-2010-10-299487
27. Kumar S, Paiva B, Anderson KC, et al. International Myeloma Working Group consensus criteria for response and minimal residual disease assessment in multiple myeloma. *The Lancet Oncology* 2016;17(8):e328-46. doi: 10.1016/S1470-2045(16)30206-6
28. Pawlyn C, Davies, F.E., Cairns, D., Striha, A., Best, P., Sigsworth, R., Jones, J.R., Kishore, B., Garg, M., Williams, C. and Karunanithi, K.,. Continuous Treatment with Lenalidomide Improves Outcomes in Newly Diagnosed Myeloma Patients Not Eligible for Autologous Stem Cell Transplant: Results of the Myeloma XI Trial. *ASH 2017* 2017
29. Jackson GH, Pawlyn C, Cairns DA, et al. Optimising the value of immunomodulatory drugs during induction and maintenance in transplant ineligible patients with newly diagnosed multiple myeloma: results from Myeloma XI, a multicentre, open-label, randomised, Phase III trial. *Br J Haematol* 2020 doi: 10.1111/bjh.16945 [published Online First: 2020/07/14]
30. O'Brien PC, Fleming TR. Multiple Testing Procedure for Clinical-Trials. *Biometrics* 1979;35(3):549-56. doi: Doi 10.2307/2530245

For peer review only - http://bmjopen.bmj.com/site/about/guidelines.xhtml

Figure Legends/ Captions

Figure 1A & Figure 1B: Overall survival in MRC-IX and NCRI-XI TE pathway (Figure 1A). Overall survival in MRC-IX and NCRI-XI TNE pathway (Figure 1B)

Figure 2: Reasons for ceasing induction treatment in NCRF=XI (n=928)

Figure 3: Flow Diagram of Myeloma XIV (FiTNEss) Trial

Figure 4: Summary of Investigations (Local and Central)

For peer review only

For peer review only - http://bmjopen.bmj.com/site/about/guidelines.xhtml

(A)

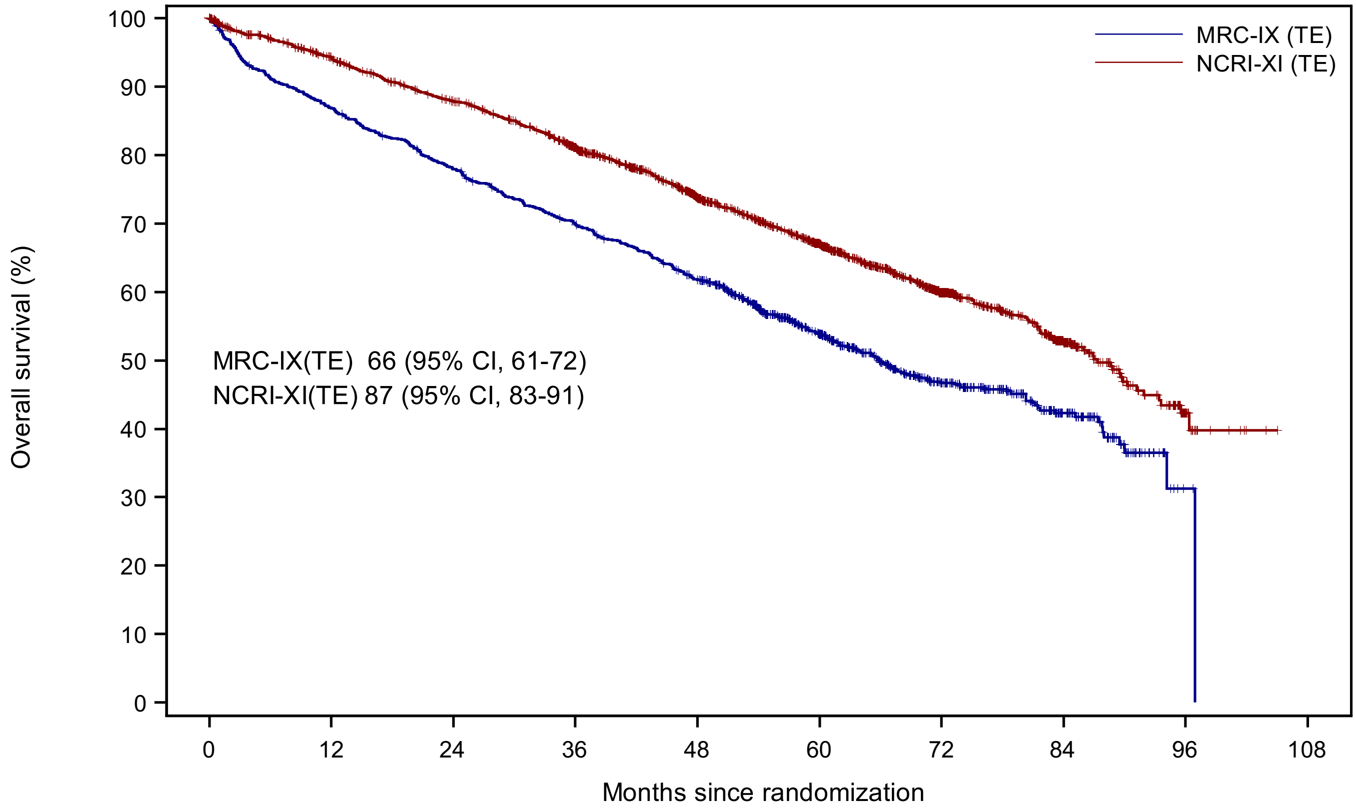

Number at risk (number censored)

	0	12	24	36	48	60	72	84	96	108
MRC-IX (TE)	1111 (0)	961 (4)	860 (7)	768 (12)	670 (21)	436 (178)	224 (345)	89 (466)	2 (545)	0 (546)
NCRI-XI (TE)	2042 (7)	1848 (80)	1689 (114)	1478 (197)	1168 (383)	868 (584)	484 (891)	196 (1E3)	24 (1E3)	0 (1E3)

(B)

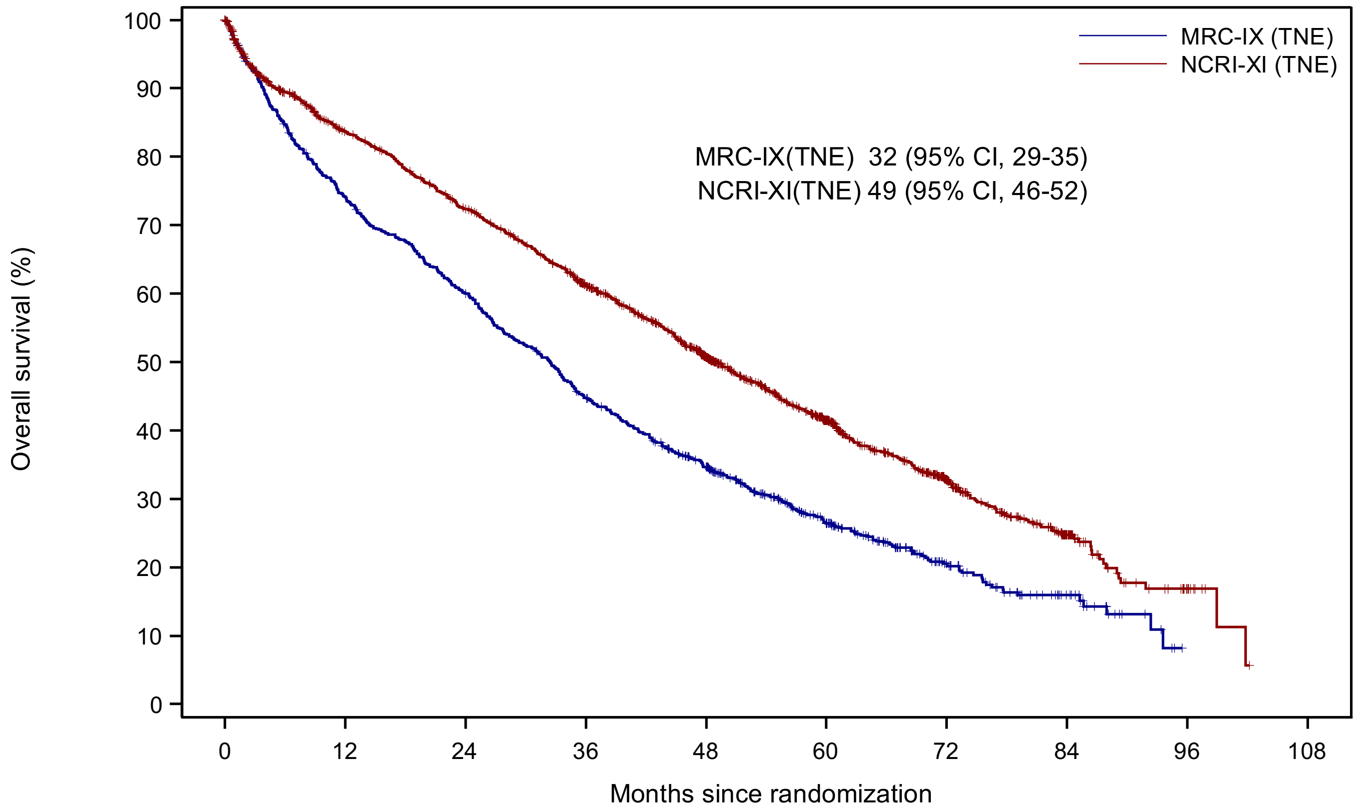

Number at risk (number censored)

	0	12	24	36	48	60	72	84	96	108
MRC-IX (TNE)	849 (1)	621 (10)	502 (11)	372 (14)	270 (34)	151 (99)	66 (158)	25 (186)	0 (206)	
NCRI-XI (TNE)	1852 (7)	1452 (108)	1244 (121)	1011 (163)	708 (304)	409 (489)	197 (631)	60 (731)	10 (769)	0 (777)

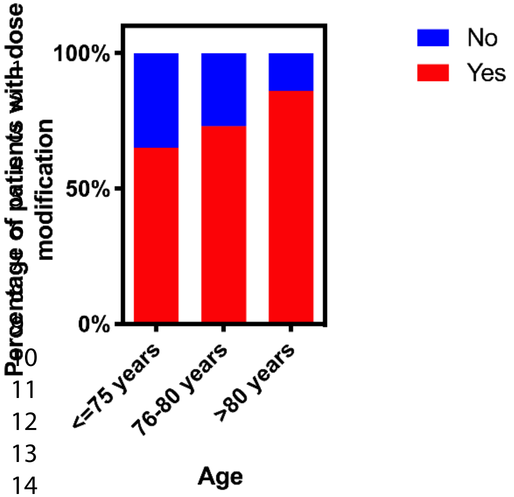
Percentage of patients with dose modification
100%
50%
0%
<=75 years
76-80 years
>80 years
Age
No
Yes

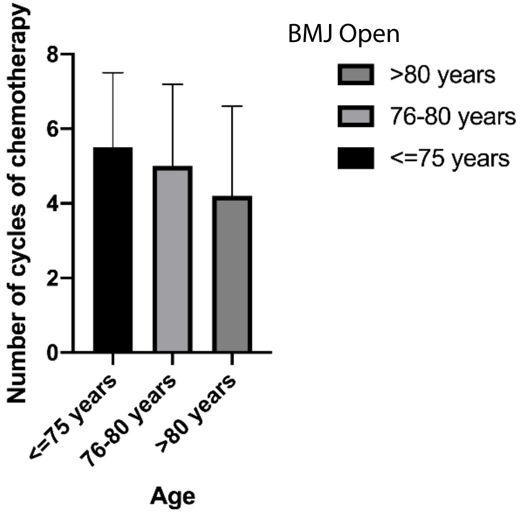
Number of cycles of chemotherapy
8
6
4
2
0
<=75 years
76-80 years
>80 years
Age
>80 years
76-80 years
<=75 years

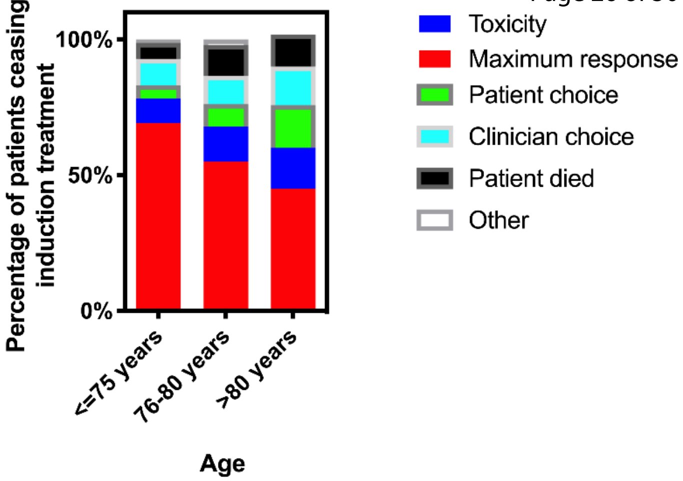
Percentage of patients ceasing induction treatment
100%
50%
0%
<=75 years
76-80 years
>80 years
Age
Toxicity
Maximum response
Patient choice
Clinician choice
Patient died
Other

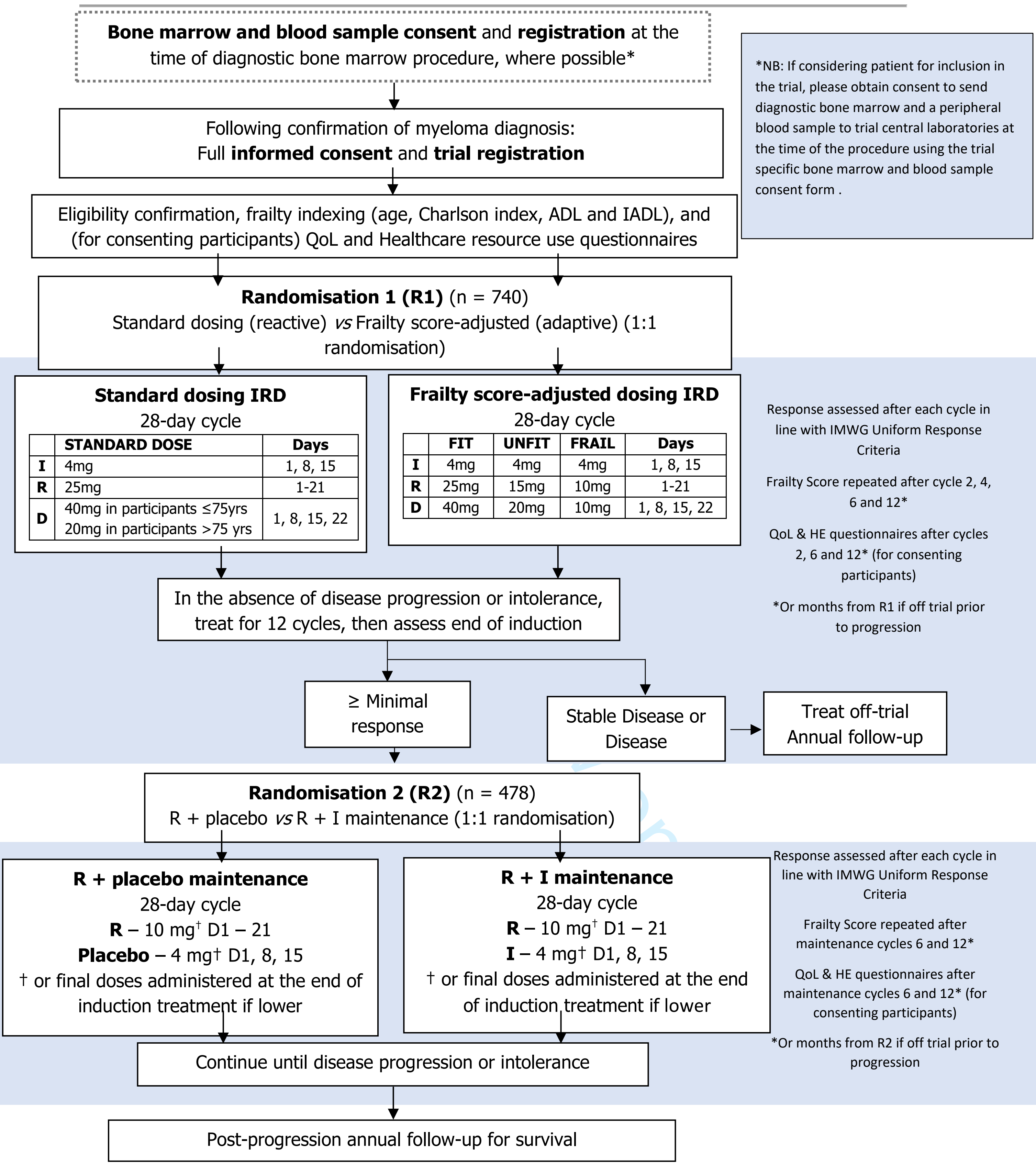

Figure 3: Flow Diagram of the trial

Figure 4: Schedule of assessments

Local Investigations	Consent & trial registration	Pre-R1 (assessments for eligibility within 14 days prior to R1, unless otherwise stated)	Day 1 (or ≤3 days prior) of each induction treatment cycle	End of 12 cycles of IRD induction treatment	Pre-Randomisation 2 (R2) (assessments for eligibility within 14 days prior to R2, do not need repeating if end of IRD assessments are within this timeframe)	Day 1 (or ≤3 days prior) of each maintenance treatment cycle	Disease Progression
Pre-trial Consent for Bone Marrow (at time of diagnostic bone marrow) and bone marrow registration	✓						
Full written informed consent and (main) trial registration	✓						
Medical history (including comorbidities, concomitant medications and previous malignancies)		✓					
Assessment of cardiac and thyroid function (as part of standard care)		✓	Monitor throughout treatment				
Physical examination (including height/weight, BP, performance status [f], vital signs [baseline only])		✓	✓	✓	✓	✓	✓
Pregnancy test (if female of child bearing potential, see Appendix 8)		✓	✓	✓	✓	✓[b]	
FBC, U&Es, calcium, creatinine, LFTs (including bilirubin, and AST and/or ALT), albumin, LDH, calculated creatinine clearance, Urinary protein:creatinine ratio* **only requested at baseline*		✓	✓[a]	✓	✓	✓	✓
CRP, β2M		✓	✓ After cycle 2, 4, and 6 [d]	✓		✓ After cycles 6 and 12 [c]	✓
Serum paraprotein, serum free light chains, serum total (class-specific) immunoglobulins and urinary light chain detection (quantification where available)		✓	✓ (To assess response to previous cycle, not applicable for C1)	✓	✓	✓ (To assess response to previous cycle)	✓
Bone marrow aspirate and (if available) trephine		✓ (Within 6 weeks prior to R1) [g]	If at any point a first occurrence of CR or sCR is suspected then bone marrow aspirate should be sent for local review as well as to HMDS, as detailed below. (s)CR cannot be confirmed without bone marrow.				✓
Serology of hepatitis B and C		✓					
Imaging		✓ (Within 3 months prior to randomisation)	Imaging of lytic and/or focal bone and extramedullary lesions if clinically indicated, in accordance with IMWG recommendations and local practice [e]				
IMWG Frailty Index (Charlson Comorbidity Score, IADL, ADL)		✓	✓ After cycle 2, 4, and 6 [d]	✓		✓ After cycles 6 and 12 [c]	
Quality of Life and Healthcare resource use questionnaires (EORTC QLQ-C30, QLQ-MY20, EQ-5D (3 Level)) **Completed by consenting participants in clinic*		✓ Before participant is informed of allocated dosing strategy	✓ After cycle 2 and 6 [d]	✓[d]		✓ After cycles 6 and 12 [c]	
Adverse Events			Monitor throughout study and report on relevant eCRFs (From randomisation until 60 days post last treatment dose)				
SAEs / SUSARs / SPMs			Monitor throughout study: all SUSARs & SAEs/SPMs must be reported to CTRU within 24 hours of the Site becoming aware of the event: refer to Section 14: (SAEs from randomisation until 60 days post last treatment dose & SPMs/SARs/SUSARs from first treatment dose until the end of trial)				

Central Analysis Investigations: Central Samples (all Participants – core consent)

Sample	Send to	Investigation	Bone marrow and blood sample consent samples <u>only</u>	Pre-randomisation 1 (pre-treatment) - Post Main Consent	During induction therapy	At the end of 12 cycles of IRD induction treatment (or at end of the final IRD cycle if sooner)	During maintenance therapy	Disease Progression
20 mL whole clotted blood or 10 mL serum	Birmingham	To confirm disease response & progression through investigation of paraprotein, immunoglobulins, serum free light chain, urinary free light chain and β2M		✓	✓ (After 2 cycles of IRD)	✓	✓ (At 2 monthly intervals)	✓
10 mL random urine				✓	✓ (After 2 cycles of IRD)	✓	✓ (At 2 monthly intervals)	✓
2 mL bone marrow aspirate in EDTA	HMDS, Leeds	To determine MRD	✓(Within 8 weeks prior to R1)	✓¥	✓ (After 6 cycles of IRD)	✓	✓ (At 12 months post-maintenance randomisation (R2))	✓
					If at any point a first occurrence of CR or sCR is suspected then bone marrow aspirate should be sent to HMDS.			
3 mL bone marrow aspirate in EDTA	ICR, London	Cytogenetic/Molecular research (including MLPA / FISH)	✓(Within 8 weeks prior to R1)	✓*¥				✓
6 mL peripheral blood in EDTA			✓(Within 8 weeks prior to R1)	✓¥				✓
20 mL peripheral blood in EDTA	LIMR, Leeds	Frailty biomarker studies		✓	✓ (After cycle 2, 4 & 6)	✓		✓
10 mL clotted blood				✓	✓ (After cycle 2, 4 & 6)	✓		✓
2 mL bone marrow aspirate in EDTA						✓ **	✓ (At 12 months post-maintenance randomisation (R2))	✓

a. The FBC should be repeated mid-cycle 1 (Day 14 +/-3 days) or more frequently and during subsequent cycles if there is a concern about cytopenias.
b. Pregnancy test must also be performed at 4 weeks after the end of study treatment.
c. Or at 6 and 12 months post-R2 if treatment is stopped prior to this for reasons other than disease progression.
d. Or at 2, 6 and 12 months post-R1 if treatment is stopped prior to this for reasons other than disease progression.
e. The imaging at baseline/pre-registration is mandatory. Subsequent imaging will be as per local protocols/standard of care - imaging will only need to be repeated if extramedullary disease was detected at baseline, or in the event of new symptoms suggestive of new extramedullary disease, cord compression, new fracture, etc., or to investigate new hypercalcaemia. In the rare event of participants with extramedullary disease at baseline, imaging should be performed at the end of IRD induction to confirm the end of induction response, and at any other time the disease parameters suggest that the participant has achieved a complete response (if not already achieved at the end of induction) to confirm that the extramedullary disease has resolved.
f. Performance status should be recorded at the same timepoints as the frailty index (i.e. prior to R1, after cycles 2, 4, 6 and 12 of induction, and after cycles 6 and 12 of maintenance). Performance status should also be recorded at the time of disease progression. Or at 2, 6 and 12 months post-R1 if treatment is stopped prior to this for reasons other than disease progression.
g. If the participant did not consent to the pre-trial bone marrow registration part of Myeloma XIV, the bone marrow biopsy will need to be taken after full informed consent.
¥ If not sent at bone marrow and blood sample consent.
* At pre-randomisation 1 a CD138 negative portion of the bone marrow aspirate will be sent to LIMR after CD138 positive selection at ICR.
** At the end of induction bone marrow aspirate will be sent to ICR after CD138 positive selection at LIMR.

Myeloma XIV Supplementary Material to Protocol Paper
EudraCT number: **2018-003590-10**

Supplementary Material:

Contents

Glossary of Terms ... 2
List of Sites in the Fitness Trial ... 4
Spirit Checklist : Reporting checklist for protocol of a clinical trial. ... 5
Renal and Liver Function ... 11
Concomitant Medication ... 12
Exploratory analysis ... 13
Subgroup analysis ... 13
Informed Consent Material ... 15
Bone marrow and Blood sample consent form ... 15
Main Trial consent ... 18
Central Sample Analysis ... 21
References ... 23

For peer review only

For peer review only - http://bmjopen.bmj.com/site/about/guidelines.xhtml

Myeloma XIV Supplementary Material to Protocol Paper
EudraCT number: **2018-003590-10**

Glossary of Terms

AE	Adverse event
ADL	Katz Activity of Daily Living
ALT	Alanine aminotransferase
AR	Adverse reaction
ANC	Absolute Neutrophil Count
AST	Aspartate aminotransferase
β2M	Beta2-microglobulin
CCI	Charlson Comorbidity Index
CI	Chief Investigator
CR	Complete response
CRF	Case report form
CTCAE	Common Terminology Criteria for Adverse Events
CTRU	Clinical Trials Research Unit
DMEC	Data Monitoring and Ethics Committee
eCRF	Electronic Case Report Form
GI	Gastrointestinal
HEAP	Health Economics Analysis Plan
HMDS	Haematological Malignancy Diagnostic Service
IADL	Lawton Instrumental Activity of Daily Living
IMiD	Immunomodulatory Drug
IMP	Investigational medicinal product
IMWG	International Myeloma Working Group
ITT	Intention to treat
mg	Milligram
MM	Multiple myeloma
MRD	Minimal Residual Disease
MR	Minimal response
NCI	National Cancer Institute
ORR	Overall response rate
OS	Overall survival
PD	Progressive disease
PFS	Progression-free survival
PFS2	Time from randomisation to second progression
PK	Pharmacokinetic(s)
PI	Proteasome Inhibitor
PR	Partial response
PS	Performance status
QoL	Quality of life
R1	Randomisation 1
R2	Randomisation 2
REC	Research Ethics Committee
SD	Stable disease
SAE	Serious adverse event
SAR	Serious adverse reaction
SPM	Secondary Primary Malignancy
SmPC	Summary of product characteristics

For peer review only - http://bmjopen.bmj.com/site/about/guidelines.xhtml
For peer review only

Myeloma XIV Supplementary Material to Protocol Paper
EudraCT number: **2018-003590-10**

SUSAR	Suspected unexpected serious adverse reaction
TMG	Trial Management Group
TNE	Transplant non-eligible
TSC	Trial Steering Committee
UK-MRA	United Kingdom Myeloma Research Alliance
ULN	Upper Limit of normal
VGPR	Very good partial response
VTE	Venous thromboembolism

For peer review only

For peer review only - http://bmjopen.bmj.com/site/about/guidelines.xhtml

Myeloma XIV Supplementary Material to Protocol Paper
EudraCT number: **2018-003590-10**

List of Sites in the Fitness Trial

Sites		
Aberdeen Royal Infirmary	Kettering general	Salford Royal Hospital
Blackpool Victoria	Kidderminster Hospital	Salisbury District Hospital
Bristol Haematology & Oncology Centre	Kings College Hospital, Denmark Hill	South Tyneside Hospital
Calderdale Royal Hospital	Leicester Royal Infirmary	Southampton Hospital
Castle Hill Hospital	Lincoln County Hospital	Southmead Hospital
Cheltenham Hospital	Maidstone Hospital	St Bartholomew Hospital
Clatterbridge Cancer Centre	Manchester Royal Infirmary	St George's Hospital, Tooting
Colchester District	New Cross Hospital	St Helens Hospital
Countess of Chester	Ninewells	St Helier Hospital
County Hospital	Norfolk & Norwich University Hospital	St Richard's Hospital
Croydon University Hospital	Nottingham City Hospital	St. James's University Hospital
Derriford Hospital	Peterborough City Hospital	St. John's. Hospital, Howden
Dewsbury and District Hospital	Pilgrim Hospital	Sunderland Royal Hospital
Dewsbury and District Hospital	Pontefract Hospital	The County Hospital, Wye Valley
Epsom General Hospital	Princess Royal	Torbay Hospital
Freeman Hospital	Queen Alexandra Hospital	TRAFFORD GENERAL HOSPITAL
Friarage Hospital	Queen Elizabeth Hospital the Queen Mother	University College London Hospital
Glan Clywd	Queen Elizabeth Hospital, Birmingham	Western General
Gloucestershire Royal	Queen Elizabeth Hospital, King's Lynn	Whiston Hospital
Goodhope	Queens Hospital, Romford	William Harvey
Grantham & District	Royal Albert Edward Infirmary	Worcestershire Royal Hospital
Guys Hospital	Royal Berkshire	Worcestershire Royal Hospital
Harrogate District Hospital	Royal Bournemouth General	Worthing Hospital
Heartlands	Royal Derby	Wrexham Maelor Hospital
Hillingdon Hospital	Royal Devon & Exeter Hospital	Wythenshaw Hospital
Hinchingbrooke Hospital	Royal Oldham	YSBYTY GWYNEDD
Huddersfield Royal Infirmary	Royal Stoke University Hospital	North Middlesex University Hospital
James Cook University Hospital	Royal United Hospital, Bath	Royal Hallamshire
Kent & Canterbury Hospital	Russells Hall Hospital	

*In total the trial will be conducted in 87 centres around the UK

For peer review only - http://bmjopen.bmj.com/site/about/guidelines.xhtml

Myeloma XIV Supplementary Material to Protocol Paper
EudraCT number: **2018-003590-10**

Spirit Checklist : Reporting checklist for protocol of a clinical trial.

Based on the SPIRIT guidelines.

Chan A-W, Tetzlaff JM, Gøtzsche PC, Altman DG, Mann H, Berlin J, Dickersin K, Hróbjartsson A, Schulz KF, Parulekar WR, Krleža-Jerić K, Laupacis A, Moher D. SPIRIT 2013 Explanation and Elaboration: Guidance for protocols of clinical trials. BMJ. 2013;346:e7586

		Reporting Item	Page Number
**Administrative information**			
Title	#1	Descriptive title identifying the study design, population, interventions, and, if applicable, trial acronym	1
Trial registration	#2a	Trial identifier and registry name. If not yet registered, name of intended registry	1,2
Trial registration: data set	#2b	All items from the World Health Organization Trial Registration Data Set	NA
Protocol version	#3	Date and version identifier	18
Funding	#4	Sources and types of financial, material, and other support	20
Roles and responsibilities: contributorship	#5a	Names, affiliations, and roles of protocol contributors	1, 20
Roles and responsibilities: sponsor contact information	#5b	Name and contact information for the trial sponsor	20
Roles and responsibilities: sponsor and funder	#5c	Role of study sponsor and funders, if any, in study design; collection, management, analysis, and interpretation of data; writing of the report; and the decision to submit the report for publication, including whether they will have ultimate authority over any of these activities	20,21
Roles and responsibilities: committees	#5d	Composition, roles, and responsibilities of the coordinating centre, steering committee, endpoint adjudication committee, data management team, and other individuals or groups overseeing the trial, if applicable (see Item 21a for data monitoring committee)	16
**Introduction**			

For peer review only

For peer review only - http://bmjopen.bmj.com/site/about/guidelines.xhtml

Myeloma XIV Supplementary Material to Protocol Paper
EudraCT number: **2018-003590-10**

Background and rationale	#6a	Description of research question and justification for undertaking the trial, including summary of relevant studies (published and unpublished) examining benefits and harms for each intervention	4,5
Background and rationale: choice of comparators	#6b	Explanation for choice of comparators	4,5,6
Objectives	#7	Specific objectives or hypotheses	6
Trial design	#8	Description of trial design including type of trial (eg, parallel group, crossover, factorial, single group), allocation ratio, and framework (eg, superiority, equivalence, non-inferiority, exploratory)	6
**Methods: Participants, interventions, and outcomes**			
Study setting	#9	Description of study settings (eg, community clinic, academic hospital) and list of countries where data will be collected. Reference to where list of study sites can be obtained	6
Eligibility criteria	#10	Inclusion and exclusion criteria for participants. If applicable, eligibility criteria for study centres and individuals who will perform the interventions (eg, surgeons, psychotherapists)	6
Interventions: description	#11a	Interventions for each group with sufficient detail to allow replication, including how and when they will be administered	9
Interventions: modifications	#11b	Criteria for discontinuing or modifying allocated interventions for a given trial participant (eg, drug dose change in response to harms, participant request, or improving / worsening disease)	9,10
Interventions: adherance	#11c	Strategies to improve adherence to intervention protocols, and any procedures for monitoring adherence (eg, drug tablet return; laboratory tests)	10
Interventions: concomitant care	#11d	Relevant concomitant care and interventions that are permitted or prohibited during the trial	10

For peer review only - http://bmjopen.bmj.com/site/about/guidelines.xhtml

Myeloma XIV Supplementary Material to Protocol Paper
EudraCT number: **2018-003590-10**

Outcomes	#12	Primary, secondary, and other outcomes, including the specific measurement variable (eg, systolic blood pressure), analysis metric (eg, change from baseline, final value, time to event), method of aggregation (eg, median, proportion), and time point for each outcome. Explanation of the clinical relevance of chosen efficacy and harm outcomes is strongly recommended	11
Participant timeline	#13	Time schedule of enrolment, interventions (including any run-ins and washouts), assessments, and visits for participants. A schematic diagram is highly recommended (see Figure)	11,12
Sample size	#14	Estimated number of participants needed to achieve study objectives and how it was determined, including clinical and statistical assumptions supporting any sample size calculations	12,13
Recruitment	#15	Strategies for achieving adequate participant enrolment to reach target sample size	13
**Methods: Assignment of interventions (for controlled trials)**			
Allocation: sequence generation	#16a	Method of generating the allocation sequence (eg, computer-generated random numbers), and list of any factors for stratification. To reduce predictability of a random sequence, details of any planned restriction (eg, blocking) should be provided in a separate document that is unavailable to those who enrol participants or assign interventions	13,14
Allocation concealment mechanism	#16b	Mechanism of implementing the allocation sequence (eg, central telephone; sequentially numbered, opaque, sealed envelopes), describing any steps to conceal the sequence until interventions are assigned	13
Allocation: implementation	#16c	Who will generate the allocation sequence, who will enrol participants, and who will assign participants to interventions	14
Blinding (masking)	#17a	Who will be blinded after assignment to interventions (eg, trial participants, care	14

For peer review only - http://bmjopen.bmj.com/site/about/guidelines.xhtml

For peer review only

		providers, outcome assessors, data analysts), and how	
Blinding (masking): emergency unblinding	#17b	If blinded, circumstances under which unblinding is permissible, and procedure for revealing a participant's allocated intervention during the trial	14
**Methods: Data collection, management, and analysis**			
Data collection plan	#18a	Plans for assessment and collection of outcome, baseline, and other trial data, including any related processes to promote data quality (eg, duplicate measurements, training of assessors) and a description of study instruments (eg, questionnaires, laboratory tests) along with their reliability and validity, if known. Reference to where data collection forms can be found, if not in the protocol	14
Data collection plan: retention	#18b	Plans to promote participant retention and complete follow-up, including list of any outcome data to be collected for participants who discontinue or deviate from intervention protocols	14
Data management	#19	Plans for data entry, coding, security, and storage, including any related processes to promote data quality (eg, double data entry; range checks for data values). Reference to where details of data management procedures can be found, if not in the protocol	14,15
Statistics: outcomes	#20a	Statistical methods for analysing primary and secondary outcomes. Reference to where other details of the statistical analysis plan can be found, if not in the protocol	15,16
Statistics: additional analyses	#20b	Methods for any additional analyses (eg, subgroup and adjusted analyses)	16
Statistics: analysis population and missing data	#20c	Definition of analysis population relating to protocol non-adherence (eg, as randomised analysis), and any statistical methods to handle missing data (eg, multiple imputation)	16,17
**Methods: Monitoring**			

For peer review only - http://bmjopen.bmj.com/site/about/guidelines.xhtml

Myeloma XIV Supplementary Material to Protocol Paper
EudraCT number: **2018-003590-10**

Data monitoring: formal committee	#21a	Composition of data monitoring committee (DMC); summary of its role and reporting structure; statement of whether it is independent from the sponsor and competing interests; and reference to where further details about its charter can be found, if not in the protocol. Alternatively, an explanation of why a DMC is not needed	16,17
Data monitoring: interim analysis	#21b	Description of any interim analyses and stopping guidelines, including who will have access to these interim results and make the final decision to terminate the trial	16,17
Harms	#22	Plans for collecting, assessing, reporting, and managing solicited and spontaneously reported adverse events and other unintended effects of trial interventions or trial conduct	17,18
Auditing	#23	Frequency and procedures for auditing trial conduct, if any, and whether the process will be independent from investigators and the sponsor	18
**Ethics and dissemination**			
Research ethics approval	#24	Plans for seeking research ethics committee / institutional review board (REC / IRB) approval	18
Protocol amendments	#25	Plans for communicating important protocol modifications (eg, changes to eligibility criteria, outcomes, analyses) to relevant parties (eg, investigators, REC / IRBs, trial participants, trial registries, journals, regulators)	18
Consent or assent	#26a	Who will obtain informed consent or assent from potential trial participants or authorised surrogates, and how (see Item 32)	19
Consent or assent: ancillary studies	#26b	Additional consent provisions for collection and use of participant data and biological specimens in ancillary studies, if applicable	19
Confidentiality	#27	How personal information about potential and enrolled participants will be collected, shared, and maintained in order to protect confidentiality before, during, and after the trial	19
Declaration of interests	#28	Financial and other competing interests for principal investigators for the overall trial and each study site	20,21

For peer review only - http://bmjopen.bmj.com/site/about/guidelines.xhtml

Myeloma XIV Supplementary Material to Protocol Paper
EudraCT number: **2018-003590-10**

Data access	#29	Statement of who will have access to the final trial dataset, and disclosure of contractual agreements that limit such access for investigators	19
Ancillary and post trial care	#30	Provisions, if any, for ancillary and post-trial care, and for compensation to those who suffer harm from trial participation	19
Dissemination policy: trial results	#31a	Plans for investigators and sponsor to communicate trial results to participants, healthcare professionals, the public, and other relevant groups (eg, via publication, reporting in results databases, or other data sharing arrangements), including any publication restrictions	19
Dissemination policy: authorship	#31b	Authorship eligibility guidelines and any intended use of professional writers	19
Dissemination policy: reproducible research	#31c	Plans, if any, for granting public access to the full protocol, participant-level dataset, and statistical code	19
**Appendices**			
Informed consent materials	#32	Model consent form and other related documentation given to participants and authorised surrogates	15 (Supplementary Material)
Biological specimens	#33	Plans for collection, laboratory evaluation, and storage of biological specimens for genetic or molecular analysis in the current trial and for future use in ancillary studies, if applicable	21 (Supplementary Material)

None The SPIRIT Explanation and Elaboration paper is distributed under the terms of the Creative Commons Attribution License CC-BY-NC. This checklist can be completed online using https://www.goodreports.org/, a tool made by the EQUATOR Network in collaboration with Penelope.ai

For peer review only - http://bmjopen.bmj.com/site/about/guidelines.xhtml

Myeloma XIV Supplementary Material to Protocol Paper
EudraCT number: **2018-003590-10**

Renal and Liver Function

As per standard of care, doses of lenalidomide and ixazomib should be adjusted at baseline according to liver and renal function. Regardless of which dosing arm has been assigned the doses indicated in this section should not be exceeded on cycle 1 day 1. Doses of lenalidomide reduced (based on renal function) can only be escalated, if tolerated, to the maximum dose indicated by the participant's assigned dosing arm/strategy after cycle 1 as per the lenalidomide SPC. Ixazomib doses may **not** be escalated, whether reduced based on renal or liver function.

Renal function:

Creatinine clearance should be calculated using the Cockcroft-Gault formula as the estimated GFR produced in most hospitals is not accurate in older patients. For moderate renal impairment, ixazomib should be given 4mg daily and should be reduced be reduced to 3mg in patients with severe renal impairment and end of stage renal disease. For moderate renal impairment, 10mg of Lenalidomide once daily should be given, 15 mg every other day for severe renal impairment and 5mg once daily at end stage renal disease.

	**Calculated creatinine Clearance (using Cockcroft-Gault formula)**	**Lenalidomide dose** (Days 1-21)	**Ixazomib dose** (Day 1, 8, 15)
Moderate renal impairment	≥ 30 to < 50 ml/min	10mg once daily	4mg once daily
Severe renal impairment	< 30 ml/min; not requiring dialysis	15mg every other day	3mg once daily
End stage renal disease	< 30 ml/min; requiring dialysis	5mg once daily (on dialysis days should be administered following dialysis)	3mg once daily

Liver function:

The reduced ixazomib dose of 3 mg is recommended in patients with moderate liver dysfunction characterised by total bilirubin > 1.5-3 x ULN (patients with bilirubin > 3 x ULN are not eligible for the study). No dose reduction based on AST/ALT is required.

Lenalidomide has not formally been studied in patients with impaired hepatic function and there are no specific dose recommendations

For peer review only - http://bmjopen.bmj.com/site/about/guidelines.xhtml

Myeloma XIV Supplementary Material to Protocol Paper
EudraCT number: **2018-003590-10**

Concomitant Medication

**Permitted Concomitant Medication:**
1) Antiemetics, including 5-HT3 serotonin receptor antagonists, may be used at the discretion of the investigator (however, note that dexamethasone additional to that given as part of the trial treatment schedule is **not** permitted).<br>2) Loperamide or other anti-diarrhoeal should be used for symptomatic non-infective diarrhoea at the discretion of the investigator. The dose and regimen will be according to institutional guidelines. Intravenous fluids should be given to prevent volume depletion.<br>3) Growth factors (e.g. granulocyte colony stimulating factor [G-CSF], thrombopoeitin receptor agonists (TPO) and recombinant erythropoietin) are permitted at the discretion of the PI and/or institutional practice. Due consideration of the increased risk of VTE with TPO agonists and erythropoietin should be given and prophylaxis adjusted accordingly.<br>4) Participants should be transfused with red cells and platelets as clinically indicated and according to institutional guidelines. Platelet transfusions are not allowed within 3 days prior to study drug dosing for any dosing day in order to meet pre-treatment criteria, to ensure that the platelet count is at a safe level to commence further treatment.<br>5) Patients known to be Hep B core antibody positive should receive prophylaxis with an appropriate anti-viral as per local protocols and after consultation with a gastroenterology/hepatology specialist.<br>6) Other supportive measures consistent with optimal participant care may be given throughout the study.
**Excluded concomitant Medication:**
1) Any antineoplastic treatment with activity against MM, other than study drugs and localised radiotherapy for pain relief.<br>2) Platelet transfusions within 3 days prior to study drug dosing for any dosing day in order to meet pre-treatment criteria, or prior to randomisation in order to meet eligibility criteria.<br>3) Additional steroid therapy (not as trial IMP) (except: before commencing trial treatment up to a maximum of 160 mg dexamethasone or equivalent based on glucocorticoid activity per inclusion criteria, or for the management of rash in which case topical, IC or oral steroid at a dose not exceeding prednisolone 10mg per day or equivalent is permitted)<br>4) Radiotherapy for the treatment of symptoms related to disease progression (in the event of disease progression participant should stop trial treatment and further treatment will be at the discretion of the treating clinician).<br>5) Excluded foods and dietary supplements include St. John's Wort.<br>6) Live vaccines are prohibited 30 days prior to R1 and for the duration of the study. They are also prohibited for 3 months after the last dose of study drug.<br>The following treatments should be avoided, unless deemed necessary by the treating clinician:<br>1) Systemic treatment with any of the following metabolising enzyme inducers should be avoided, unless there is no appropriate alternative medication for the participant's use (Rationale: If there were to be a drug-drug interaction with an inducer, ixazomib exposure would be decreased):<br>2) Strong CYP3A inducers: rifampicin, rifabutin, carbamazepine, phenytoin, & phenobarbital<br>3) Non-steroidal anti-inflammatory drugs (NSAIDs) should be avoided especially in those with abnormal renal function unless the clinical benefit outweighs the risk to deteriorating renal impairment.

For peer review only - http://bmjopen.bmj.com/site/about/guidelines.xhtml

Myeloma XIV Supplementary Material to Protocol Paper
EudraCT number: **2018-003590-10**

Exploratory analysis

Exploratory analysis will consider; the association of molecular subgroups with outcome through the investigation of frailty biomarkers, differences between the Karnofsky Performance Status (PS) and ECOG PS, and the prospective validation the UK-MRA MRP (United Kingdom-Myeloma Research Alliance Myeloma Risk Profile). All exploratory analysis to be conducted by the trial statisticians will be pre-specified in the statistical analysis plan.

Subgroup analysis

A series of subgroup analyses will be conducted to determine whether a selection of patient characteristics, haematology / local serological results, biochemistry results and cytogenetic/molecular results as well as response assessments are prognostic of the endpoints; Progression-Free survival, Progression-free survival 2, overall survival, attainment of ≥VGPR (Very Good Partial Response) and attainment of Minimal Residual Disease (MRD) negativity, as defined for both randomisations. Note that the subgroups MRD response to IRD (Ixazomib, Lenalidomide and Dexamethasone) induction and MRD response at 12m maintenance will not be analysed for the attainment of MRD negativity endpoint.

Patient Characteristics

- Age at R1 baseline (<70,70-80,>80)
- Sex (Male / Female)
- ECOG performance status at R1 baseline (0,1, ≥2, missing)
- Karnofsky performance status at R1 baseline (<80, 80-90,>90, missing)
- ISS at R1 baseline (Stage I, Stage II, Stage III, missing)
- R-ISS at R1 baseline (Stage I, Stage II, Stage III, missing)
- IMWG frailty category at R1 baseline (Fit, Unfit, Frail)
- MRP frailty category at R1 baseline (Fit, Intermediate-Fitness, Frail, missing)

Hematology / Local Serological Results

- Haemaglobin concentration at R1 baseline (<100, ≥100 g/L)
- Platelets at R1 baseline (<150, ≥150 x109/L)
- White blood cells at R1 baseline (<LLN, ≥LLN, missing)
- Neutrophil count at R1 baseline (<LLN, ≥LLN)
- Lymphocyte count at R1 baseline (<LLN, ≥LLN, missing)
- Plasma cells (%) in bone marrow at R1 baseline (<60%, ≥60%)

Biochemistry Results

- β-2 microglobulin concentration at R1 baseline (<3.5, 3.5–<5.5, ≥5.5mg/L)
- Serum creatinine concentration at R1 baseline (<175, ≥175 μmol/L)
- Corrected serum calcium concentration at R1 baseline (<2.75, ≥2.75 mmol/L)
- Serum albumin at R1 baseline (<ULN, ≥ULN, missing)
- Lactate dehydrogenase at R1 baseline (<ULN, ≥ULN, missing)
- C-Reactive protein at R1 baseline (<ULN, ≥ULN, missing)
- Total bilirubin at R1 baseline (<ULN, ≥ULN)
- ALT/AST at R1 baseline (<ULN, ≥ULN)

For peer review only - http://bmjopen.bmj.com/site/about/guidelines.xhtml

Myeloma XIV Supplementary Material to Protocol Paper
EudraCT number: **2018-003590-10**

MLPA Cytogenetic Results

- t(4,14) at R1 baseline (detected, not detected)
- t(14,16) at R1 baseline (detected, not detected)
- t(14,20) at R1 baseline (detected, not detected)
- del(17p) at R1 baseline (detected, not detected)
- del(13q) at R1 baseline (detected, not detected)
- del(1p) at R1 baseline (detected, not detected)
- gain(1q) at R1 baseline (detected, not detected)
- UK definition risk group at R1 baseline (Standard, high risk, ultra-high risk)
- IMWG definition risk group at R1 baseline (Adverse, Standard)

Response

- Response to IRD induction (<VGPR, ≥ VGPR)
- Response to 12m maintenance (<VGPR, ≥ VGPR)
- MRD response to IRD induction (MRD positive, MRD Negative)
- MRD response to 12m maintenance (MRD positive, MRD Negative)

Further subgroup analyses may be undertaken, as appropriate and all subgroup analysis will be fully documented in the statistical analysis plan. Subgroup and exploratory analyses may, by chance, generate false negative or positive results. Those carried out will be interpreted with caution.

For peer review only

For peer review only - http://bmjopen.bmj.com/site/about/guidelines.xhtml

Myeloma XIV Supplementary Material to Protocol Paper
EudraCT number: **2018-003590-10**

Informed Consent Material

Bone marrow and Blood sample consent form

Delete this line, then print on Trust/Hospital headed paper

Participant ID:	Initials:
Date of Birth:	NHS/Hospital Number:
EudraCT Number:	Principal Investigator:

FiTNEss
UK-MRA Myeloma XIV

PARTICIPANT CONSENT FORM FOR BONE MARROW AND BLOOD SAMPLE

Please initial each box

1. I confirm that I have read and understand the information sheet for the bone marrow and blood sample request and have had the opportunity to ask questions.

2. I understand that my providing bone marrow and blood samples is voluntary and that I am free to withdraw at any time without my medical care or legal rights being affected.

3. I understand that the samples will be sent along with information about me, which will include NHS number and may include my name.

4. I agree to these samples being stored and used for research investigations that form part of the FiTNEss (UK-MRA Myeloma XIV) study, and understand that this will include genetic research.

5. I understand that a unique reference number will be allocated to the samples which may allow them to be linked back to me in future for research purposes.

6. I understand that my data may be shared on a collaborative basis with researchers in the UK and potentially, centres abroad, including outside the European Economic Area.

For peer review only - http://bmjopen.bmj.com/site/about/guidelines.xhtml

7. I agree to allow any information provided to be used for healthcare and/or further medical research upon the understanding that my identity will remain anonymous wherever possible. ☐

8. I understand that results of my laboratory tests may be shared with other research teams if I am taking part in another clinical study being run by the University of Leeds. ☐

9. I agree to a copy of this Consent Form being sent to the Clinical Trials Research Unit. ☐

10. I agree for my details (which will include date of birth and NHS number) to be submitted to NHS Digital so that information about my health, and mortality should this become available, may be obtained by the CTRU if necessary. ☐

11. I agree to provide bone marrow and blood samples. ☐

Please indicate your wishes in the below scenarios:

Please tick or initial yes or no:

Please tick ✓ or initial

	Yes	No
A) If I am diagnosed with a plasma cell dyscrasia, other than myeloma, I give permission for the samples sent to the central laboratories to be stored and used in future research that receives ethical approval. I understand that the samples and data collected from them may be shared with researchers, possibly outside the EEA.	☐	☐
B) If I am diagnosed with myeloma but do not take part in the FiTNEss (UK-MRA Myeloma XIV) study, I give permission for the samples sent to the central laboratories to be stored and used in future research that receives ethical approval. I understand that the samples and data collected from them may be shared with researchers, possibly outside the EEA.	☐	☐

For peer review only - http://bmjopen.bmj.com/site/about/guidelines.xhtml

Myeloma XIV Supplementary Material to Protocol Paper
EudraCT number: **2018-003590-10**

Patient (to be completed by the patient):

Signature………..

Name (block capitals)………………………………………………………………………………………………..

Date……..

Investigator:

I have explained the request to the above named patient and he/she has indicated his/her willingness for samples of bone marrow and blood to be sent to the FiTNEss central laboratories.

Signature………

Name (block capitals)………………………………………………………………………………………………..

Date……

(If used)Translator:

Signature………

Name (block capitals)………………………………………………………………………………………………..

Date……

(1 copy for patient; 1 for the CTRU; 1 held in patient notes, original stored in Investigator Site File)

.

For peer review only

For peer review only - http://bmjopen.bmj.com/site/about/guidelines.xhtml

Myeloma XIV Supplementary Material to Protocol Paper
EudraCT number: **2018-003590-10**

Main Trial consent

Delete this line, then print on Trust/Hospital headed paper

Participant ID:	Initials:
Date of Birth:	NHS/Hospital Number:
EudraCT Number:	Principal Investigator:

FiTNEss
UK-MRA Myeloma XIV

PARTICIPANT CONSENT FORM

Please initial each box

1. I confirm that I have read and understand the information sheet for the above study and have had the opportunity to ask questions.

2. I understand that my participation in this study is voluntary and that I am free to withdraw at any time without my medical care or legal rights being affected. I understand that even if I withdraw from the above study, the data and samples collected from me will be used in analysing the results of the study, and in some cases further information about any unwanted effects of my treatment may need to be collected by the study team.

3. I understand that my healthcare records may be looked at by authorised individuals from the study team, regulatory bodies or Sponsor in order to check that the study is being carried out correctly.

4. I understand that I will be asked questions about how independent my lifestyle is. I understand that this information will be used to generate a score of frailty and may affect the doses of treatment that I receive.

5. I am aware that I am required to have samples taken and sent to a central laboratory and I understand that this will include genetic research. I understand that the samples will be sent along with

For peer review only - http://bmjopen.bmj.com/site/about/guidelines.xhtml

information about me, which will include NHS number and may include my name.

6. I understand that results or reports from imaging scans, blood tests or other investigations (including for additional cancers) may be sent to the Clinical Trials Research Unit for central review by the research team.

7. I agree to allow any information or results arising from this study to be used for healthcare and/or further medical research upon the understanding that my identity will remain anonymous wherever possible.

8. I understand that my data and results of my laboratory tests (blood, urine and bone marrow sample collections) may be shared with other research teams if I am taking part in another clinical study being run by the University of Leeds.

9. I agree for my details (which will include date of birth and NHS number) to be submitted to NHS Digital so that information about my health, and mortality should this become available, may be obtained by the CTRU if necessary.

10. I understand that my GP, or any other doctor treating me, will be notified of my participation in this study.

11. I understand that some of my data may be passed to other organisations, such as the pharmaceutical companies Takeda and Celgene, (possibly in other countries outside the EEA where the data protection standards and laws may be different from the UK) to monitor the safety of the treatment(s) that I am receiving. I understand that my identity will remain anonymous.

12. I agree to a copy of this Consent Form being sent to the Clinical Trials Research Unit

13. I agree to take part in the study.

For peer review only - http://bmjopen.bmj.com/site/about/guidelines.xhtml

Myeloma XIV Supplementary Material to Protocol Paper
EudraCT number: **2018-003590-10**

The following parts of the study are OPTIONAL (see part 3 of the Participant Information Sheet). Even if you agree to take part in the FiTNEss study, you do not have to agree to these optional parts. Please tick or initial yes or no.

Please tick ✓ or initial

	Yes	No
A) I agree to take part in the Quality of Life and Healthcare Resource Use studies by completing questionnaires at time points throughout the study.	☐	☐
B) I give permission for samples that I provide to be used in future research that receives ethical approval, and I understand that this includes the blood and bone marrow samples provided at the time of my diagnosis (if consented separately to the bone marrow and blood sample for this study). I understand that my samples and data collected from them may be shared on a collaborative basis with researchers in the UK and potentially, centres abroad, including outside the European Economic Area.	☐	☐

Patient (to be completed by the patient):

Signature………………………………………………………………………………

Name (block capitals)……………………………………………………………………

Date……………………………………………………………………………………

Investigator:

I have explained the study to the above named patient and he/she has indicated his/her willingness to participate.

Signature………………………………………………………………………………

Name (block capitals)……………………………………………………………………

Date……………………………………………………………………………………

(If used)Translator:

Signature………………………………………………………………………………

Name (block capitals)……………………………………………………………………

Date……………………………………………………………………………………

For peer review only - http://bmjopen.bmj.com/site/about/guidelines.xhtml

Myeloma XIV Supplementary Material to Protocol Paper
EudraCT number: **2018-003590-10**

Central Sample Analysis

The trial has four central laboratories, each receiving samples for trial purposes. The time points and the reason for analysis for each central sample can be found in Figure 4. Details on the analysis of each sample is below. Note that participants can consent for their samples to be used in future research and hence each laboratory can store their trial samples for use in future studies. The storage details are also included below.

Birmingham

Samples are sent in approved packaging via the UK post office service and received in a UKAS accredited clinical laboratory for laboratory assessment and storage under the direction of the TMG. Assessment of prognostic markers, markers of disease activity and M-protein levels. Analyses will include serum levels of beta-2 microglobulin, immunoglobulins IgG, IgA and IgM, kappa and lambda free immunoglobulin light chains. Serum electrophoresis and immunofixation, M-protein quantitation including total protein and densitometry. Urine immunofixation and free light chain quantitation will be performed where appropriate.

HMDS

The presence of minimal residual disease will be assessed in bone marrow aspirates using a validated flow cytometry assay (sensitivity ≤0.004%) performed at a single central laboratory (HMDS, Leeds Teaching Hospitals Trust). A minimum of 500,000 cells will be evaluated with six- or eight-colour antibody combinations including CD138, CD38, CD45, CD19, CD56, CD27, CD81 and CD117.

Sustained MRD-negativity, as defined by the IMWG [1], will not be assessed as it requires the participant to be imaging MRD-negative at the same timepoints that they are flow MRD-negative and imaging is only mandated at baseline and to investigate new systems not at regular intervals for all participants.

Flow MRD will be assessed at the following time points: Baseline (pre-randomisation 1), after 6 cycles of induction therapy, at the end of 12 cycles of induction treatment (or at the end of the final cycle of IRD treatment if sooner), at 12 months post maintenance randomisation 2. Flow MRD will also be assessed at any point a first occurrence of CR, or SCR is suspected.

During final analysis, we will determine the conversion rate of patients who are MRD Positive at the end of induction to MRD Negative at 12 months post Randomisation 2. The analysis to this is specified within our Statistical Analysis Plan (SAP).

Samples will be used for trial purposes only and will not be stored for future research.

ICR

Myeloma tumor material is purified from fresh bone marrow material using CD138 immunomagnetic separation. Tumor DNA and RNA are extracted and recurrent immunoglobulin translocations t(4;14), t(14;16), t(14;20) and copy number aberrations del(1p32), gain(1q21), del(17p) investigated as previously published using qRT-PCR and multiplex ligation-dependent amplification (MLPA), respectively[2]. Excess material is stored, if patient gave written consent for use in future research, at -80C standard DNA and RNA storage conditions.

LIMR

For peer review only - http://bmjopen.bmj.com/site/about/guidelines.xhtml

Myeloma XIV Supplementary Material to Protocol Paper
EudraCT number: **2018-003590-10**

Peripheral blood samples for immunosenescent and Senescence-associated secretory proteome will be draw and managed timepoints in the clinical pathway, processed in a central laboratory and stored as aliquots of mononuclear cells and serum for subsequent planned use. Full QA is performed on sample receipt to ensure only viable and valuable samples are processed and stored for research use.

For peer review only

Myeloma XIV Supplementary Material to Protocol Paper
EudraCT number: **2018-003590-10**

References

1. Kumar, S.P., et al., *International Myeloma Working Group consensus criteria for response and minimal residual disease assessment in multiple myeloma.* The lancet oncology, 2016. **17**(8): p. e328-e346.
2. Shah, V., et al., *Prediction of outcome in newly diagnosed myeloma: a meta-analysis of the molecular profiles of 1905 trial patients.* Leukemia, 2018. **32**(1): p. 102-110.

For peer review only
